# Supplementary figures and images for: Physiological febrile heat stress increases cytoadhesion through increased protein trafficking of Plasmodium falciparum surface proteins into the red blood cell
Source: eLife. 2026 May 13;14:RP107860. doi: 10.7554/eLife.107860 (PMC13171106; doi:10.7554/eLife.107860)

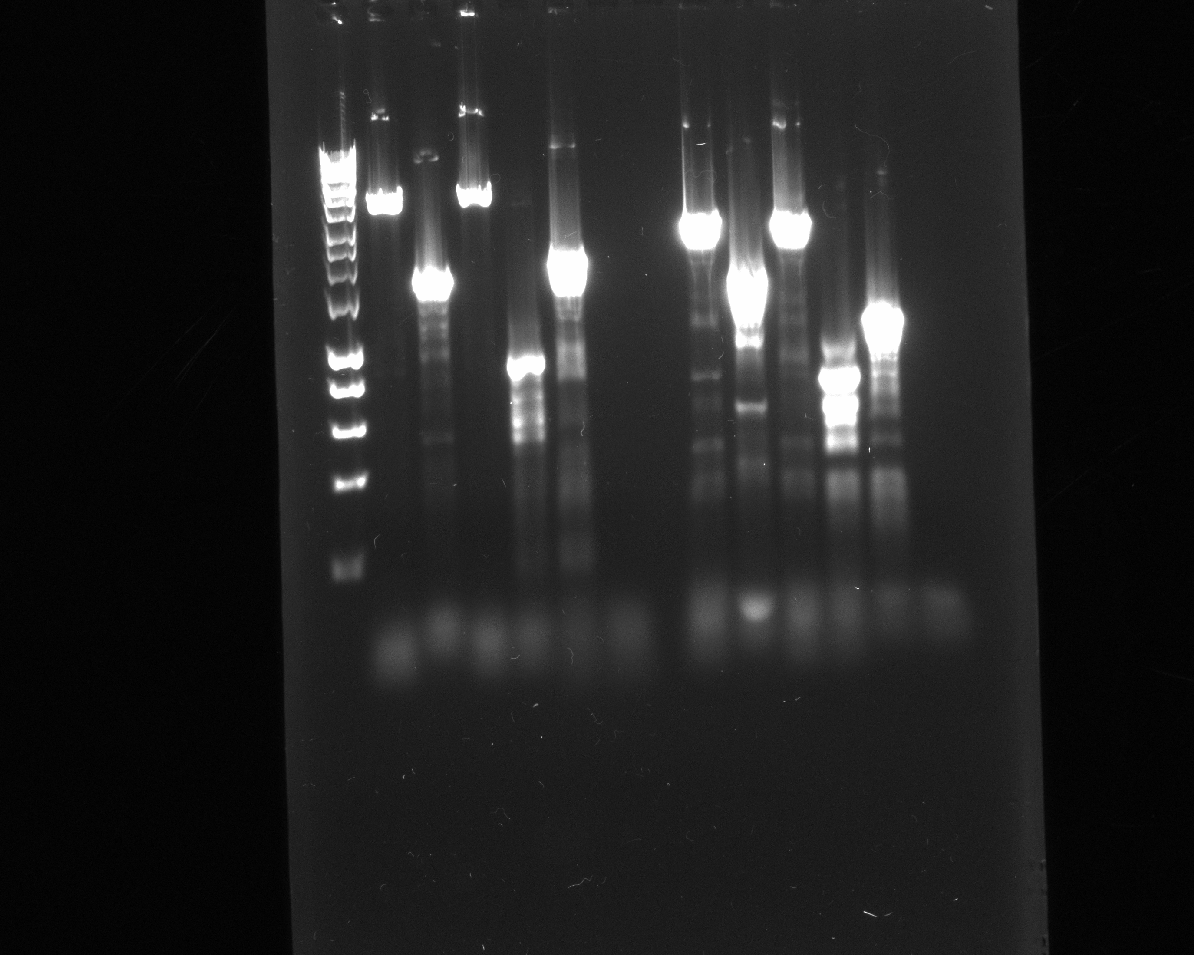

Supplement: Figure 3—figure supplement 3—source data 1. [file elife-107860-fig3-figsupp3-data1.zip › Supplement 3 - Source Data 1 - Raw Images/Figure 3 - Supplement 3 - Source Data 1 - Raw High Contrast Image File.tif]

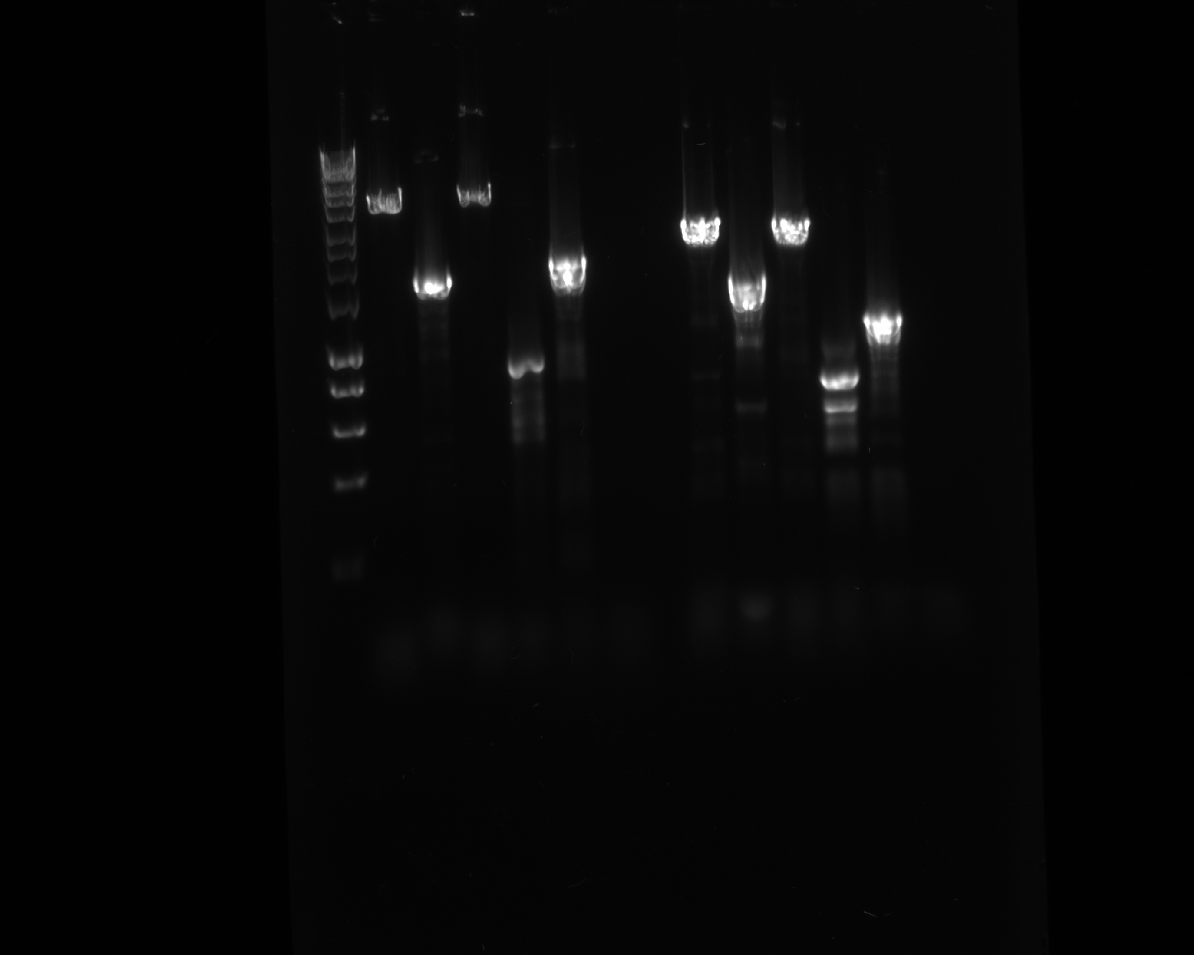

Supplement: Figure 3—figure supplement 3—source data 1. [file elife-107860-fig3-figsupp3-data1.zip › Supplement 3 - Source Data 1 - Raw Images/Figure 3 - Supplement 3 - Source Data 1 - Raw Image File.tif]

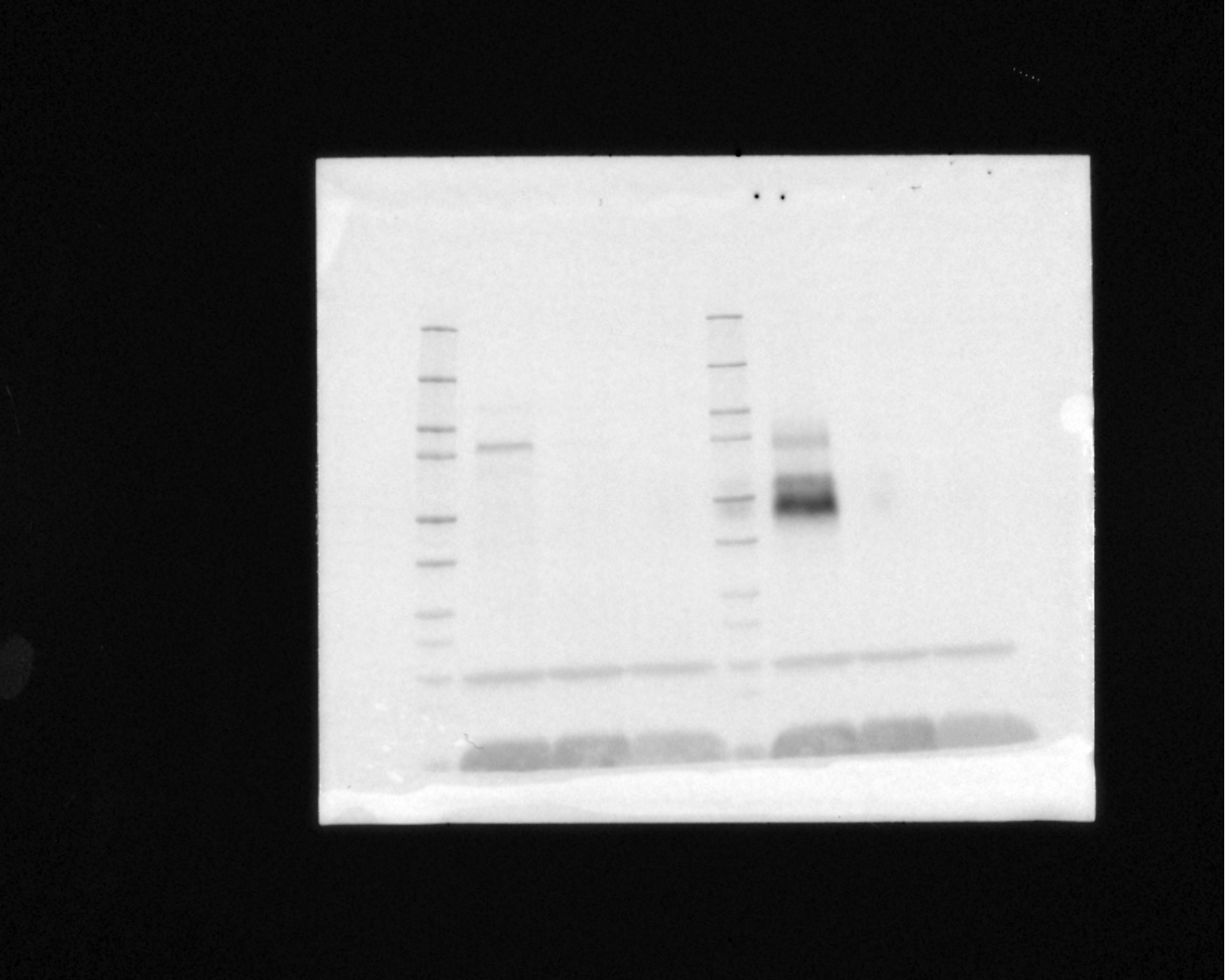

Supplement: Figure 3—figure supplement 3—source data 2. [file elife-107860-fig3-figsupp3-data2.zip › Figure 3 - Supplement 3 - Source Data 2 - Raw Images/Figure 3 - Supplement 3 - Source Data 2 - Raw Image a-HA.tif]

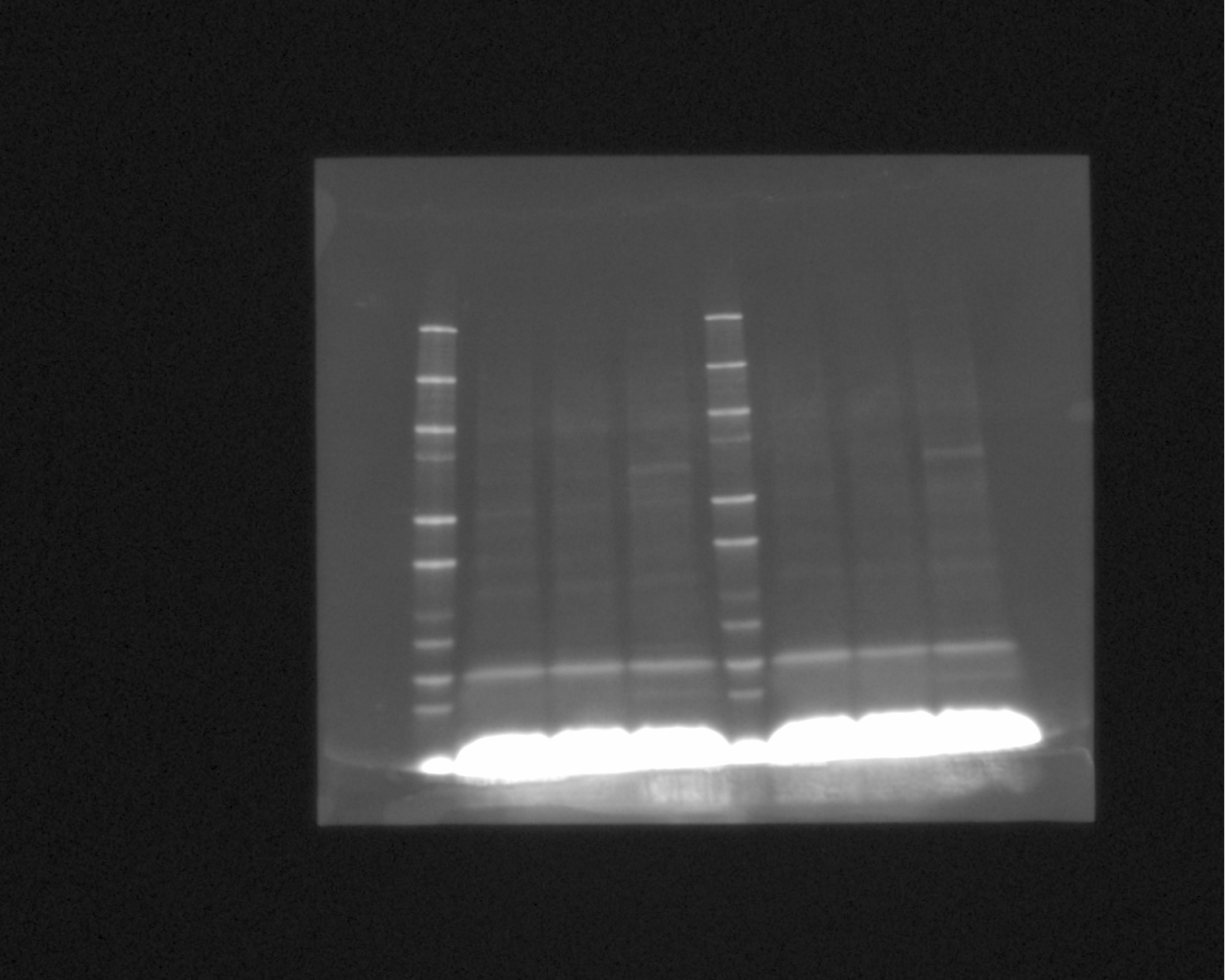

Supplement: Figure 3—figure supplement 3—source data 3. [file elife-107860-fig3-figsupp3-data3.zip › Supplement 3 - Source Data 3 - Raw Image/Figure 3 - Supplement 3 - Source Data 3 - Raw Image Total Protein.tif]

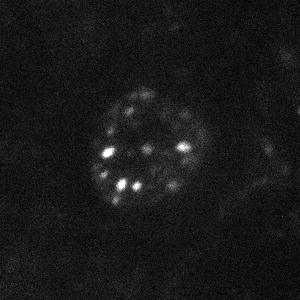

Supplement: Figure 3—figure supplement 4—source data 1. — Raw microscopy images of transgenic HSP70x-3xHA parasites probed with α-HA and α-SBP1. [file elife-107860-fig3-figsupp4-data1.zip › Figure 3 - Supplement 4 - Source Data 1 Raw Images/Cropped/DMSO_HA_SBP1003.nd2 - T=0-1.tif]

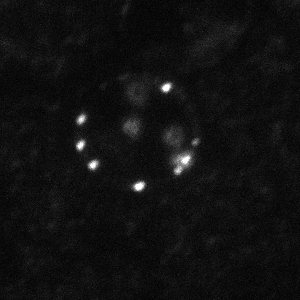

Supplement: Figure 3—figure supplement 4—source data 1. — Raw microscopy images of transgenic HSP70x-3xHA parasites probed with α-HA and α-SBP1. [file elife-107860-fig3-figsupp4-data1.zip › Figure 3 - Supplement 4 - Source Data 1 Raw Images/Cropped/DMSO_HA_SBP1006.nd2 - T=0-1.tif]

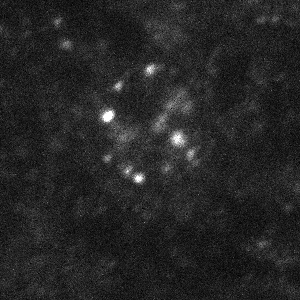

Supplement: Figure 3—figure supplement 4—source data 1. — Raw microscopy images of transgenic HSP70x-3xHA parasites probed with α-HA and α-SBP1. [file elife-107860-fig3-figsupp4-data1.zip › Figure 3 - Supplement 4 - Source Data 1 Raw Images/Cropped/DMSO_HA_SBP1008.nd2 - T=0-1.tif]

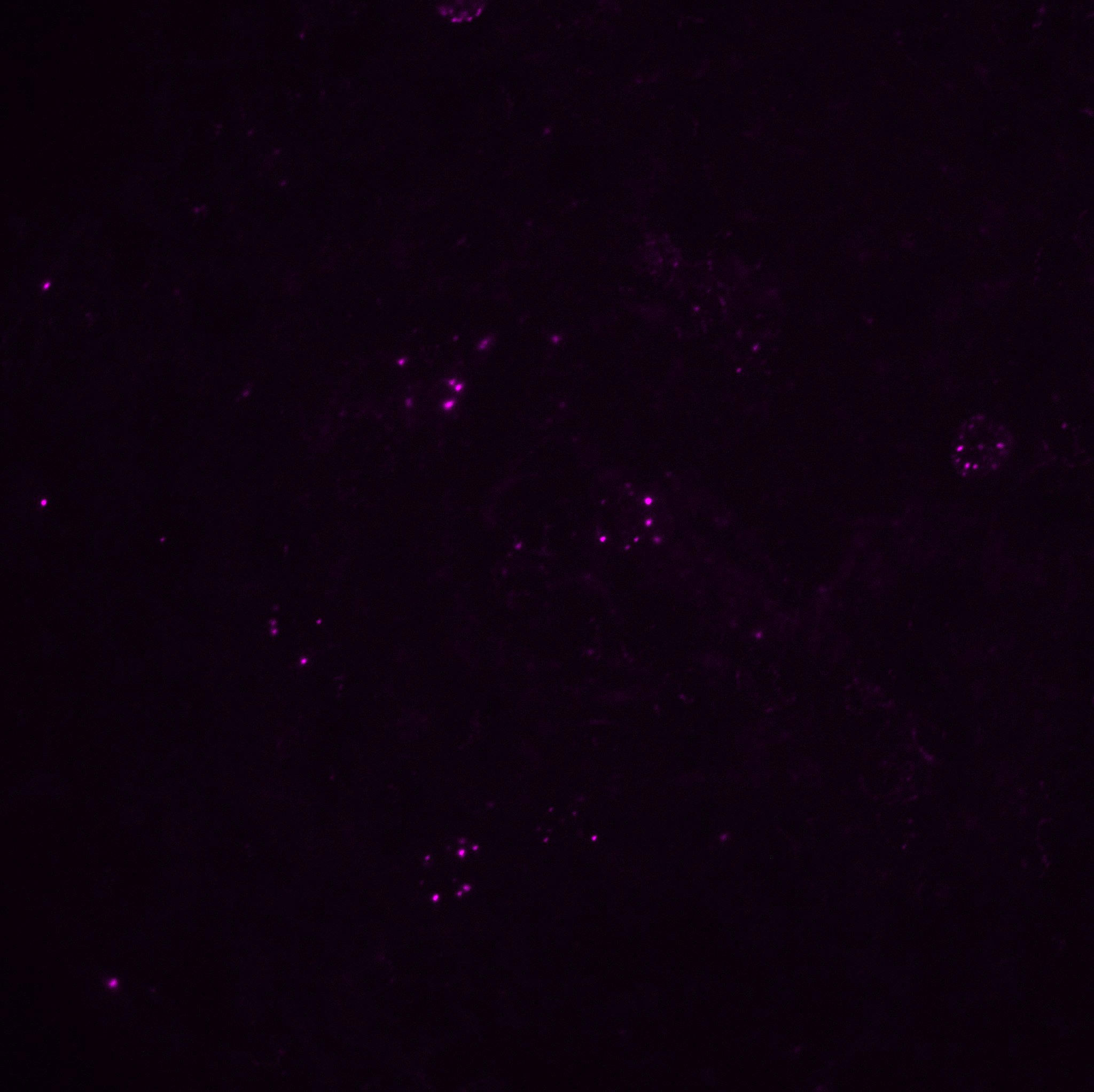

Supplement: Figure 3—figure supplement 4—source data 1. — Raw microscopy images of transgenic HSP70x-3xHA parasites probed with α-HA and α-SBP1. [file elife-107860-fig3-figsupp4-data1.zip › Figure 3 - Supplement 4 - Source Data 1 Raw Images/DMSO_HA_SBP1003-0001.jpg]

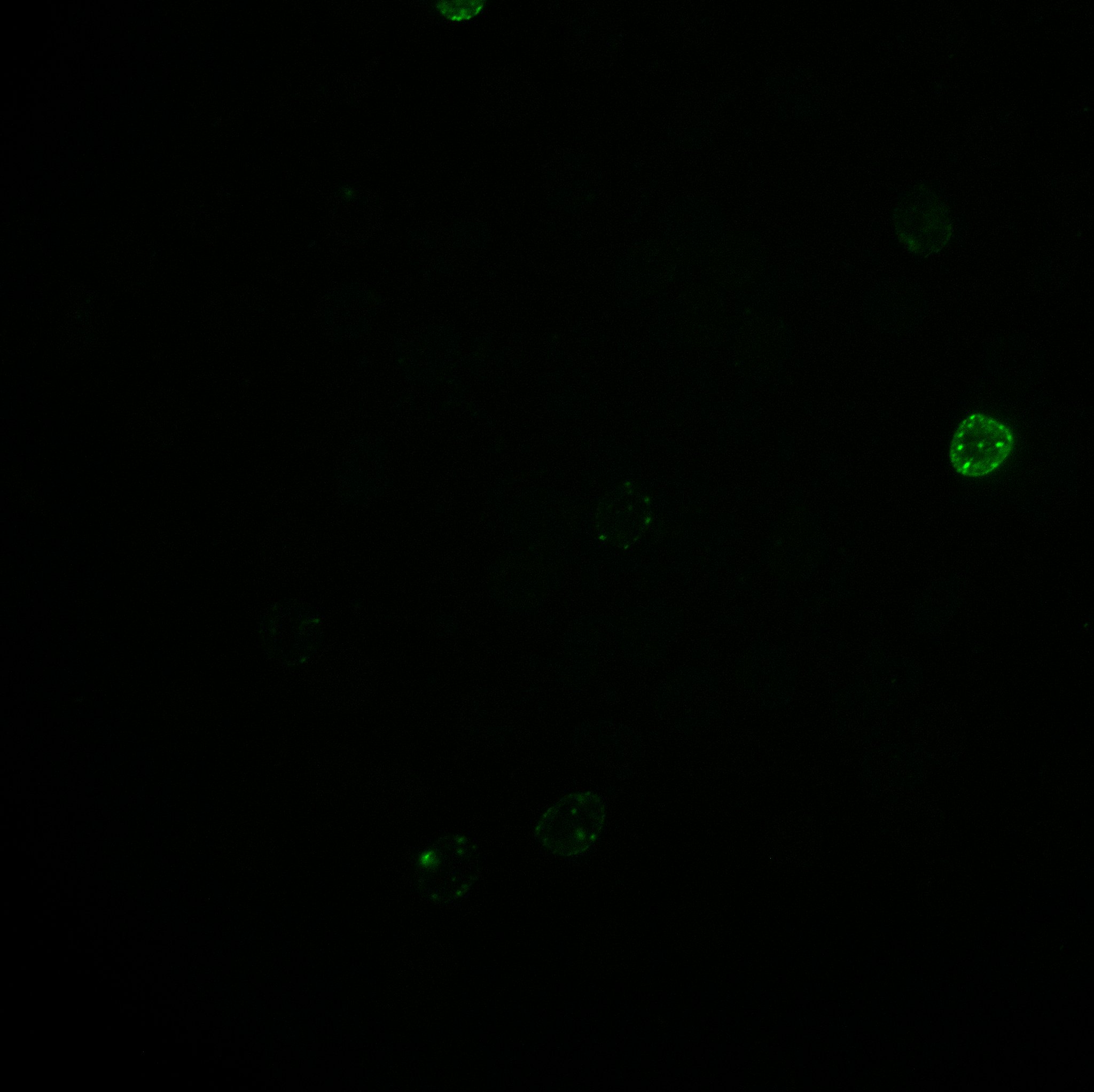

Supplement: Figure 3—figure supplement 4—source data 1. — Raw microscopy images of transgenic HSP70x-3xHA parasites probed with α-HA and α-SBP1. [file elife-107860-fig3-figsupp4-data1.zip › Figure 3 - Supplement 4 - Source Data 1 Raw Images/DMSO_HA_SBP1003-0002.jpg]

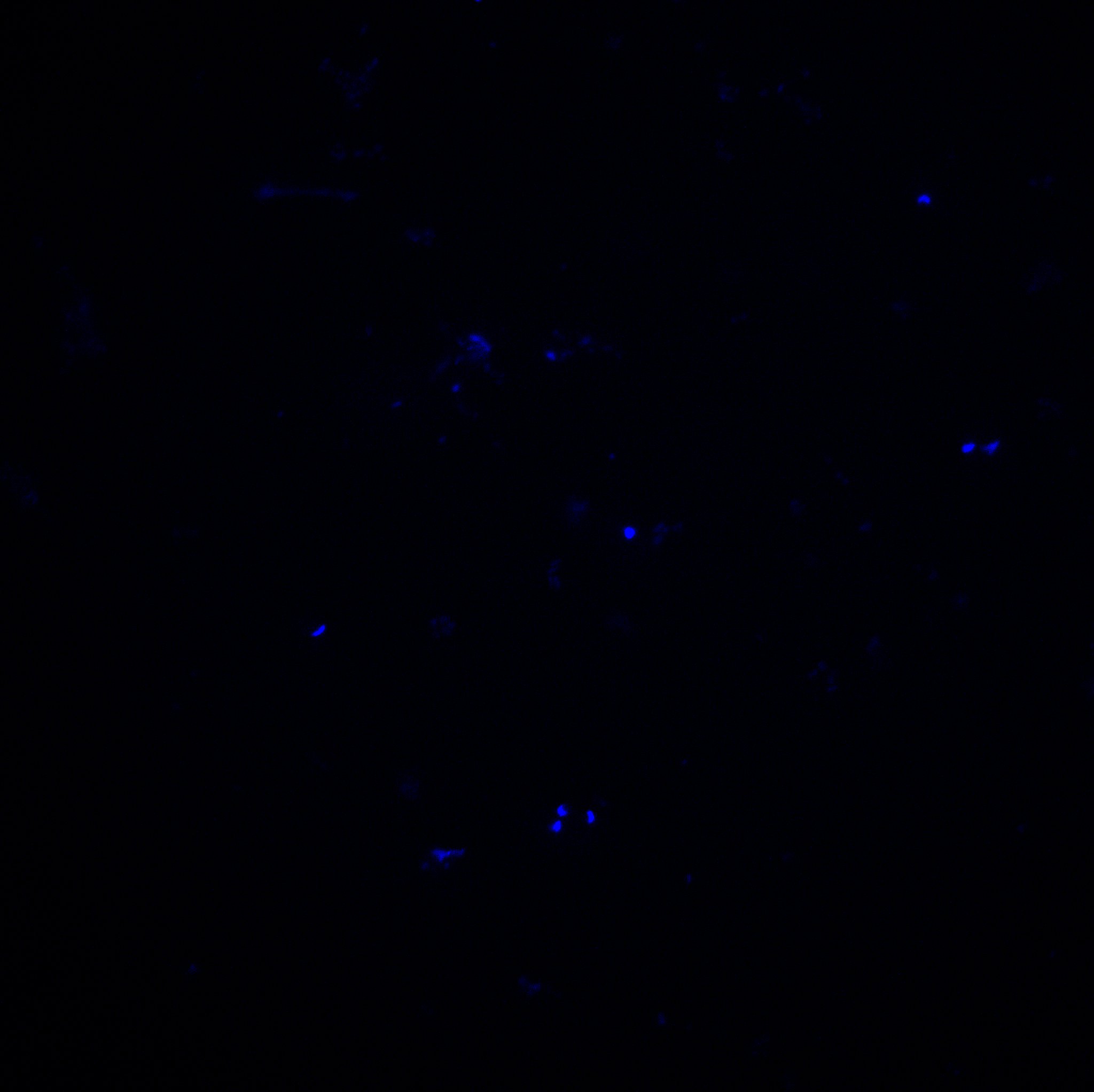

Supplement: Figure 3—figure supplement 4—source data 1. — Raw microscopy images of transgenic HSP70x-3xHA parasites probed with α-HA and α-SBP1. [file elife-107860-fig3-figsupp4-data1.zip › Figure 3 - Supplement 4 - Source Data 1 Raw Images/DMSO_HA_SBP1003-0003.jpg]

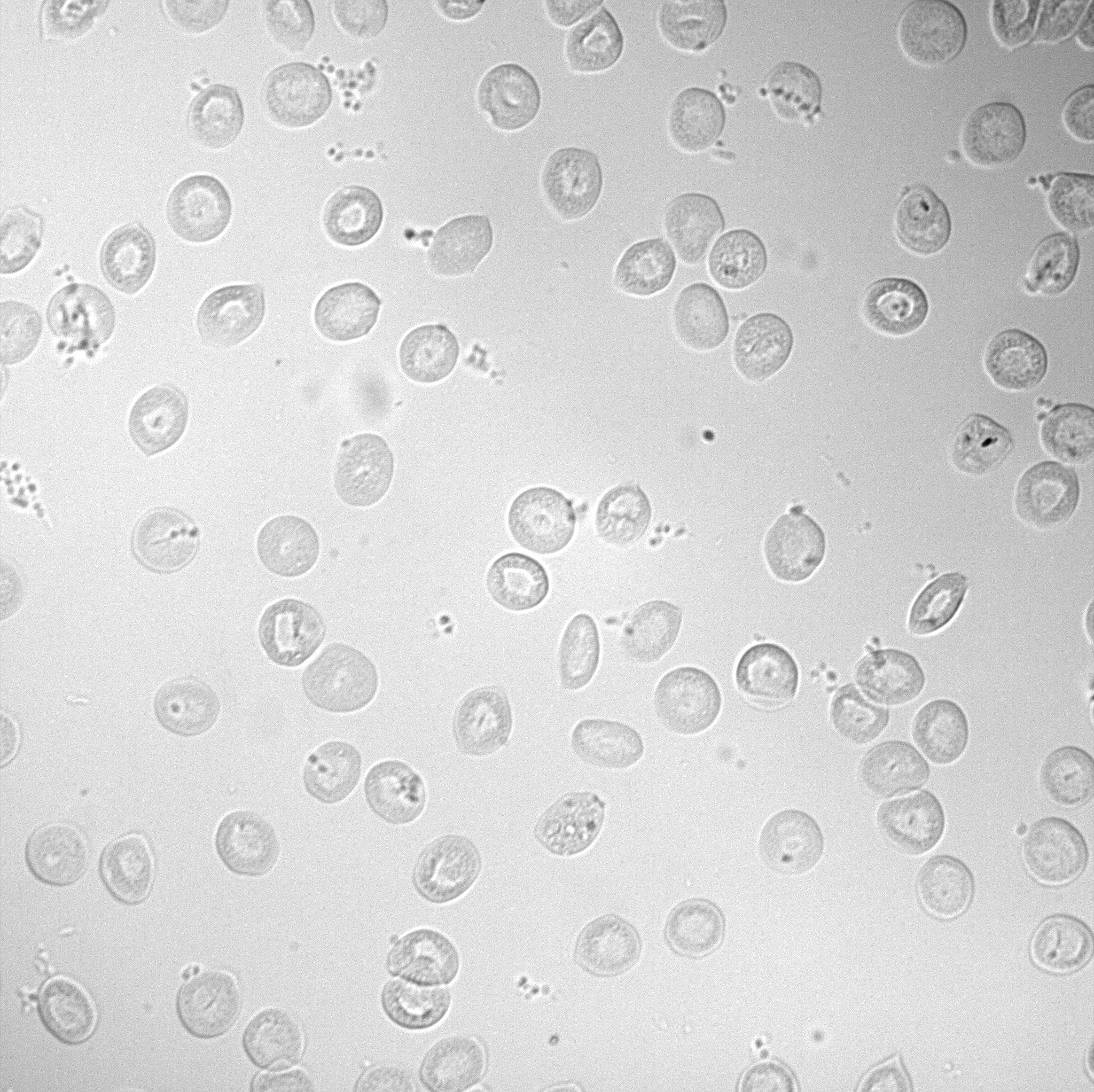

Supplement: Figure 3—figure supplement 4—source data 1. — Raw microscopy images of transgenic HSP70x-3xHA parasites probed with α-HA and α-SBP1. [file elife-107860-fig3-figsupp4-data1.zip › Figure 3 - Supplement 4 - Source Data 1 Raw Images/DMSO_HA_SBP1003-0004.jpg]

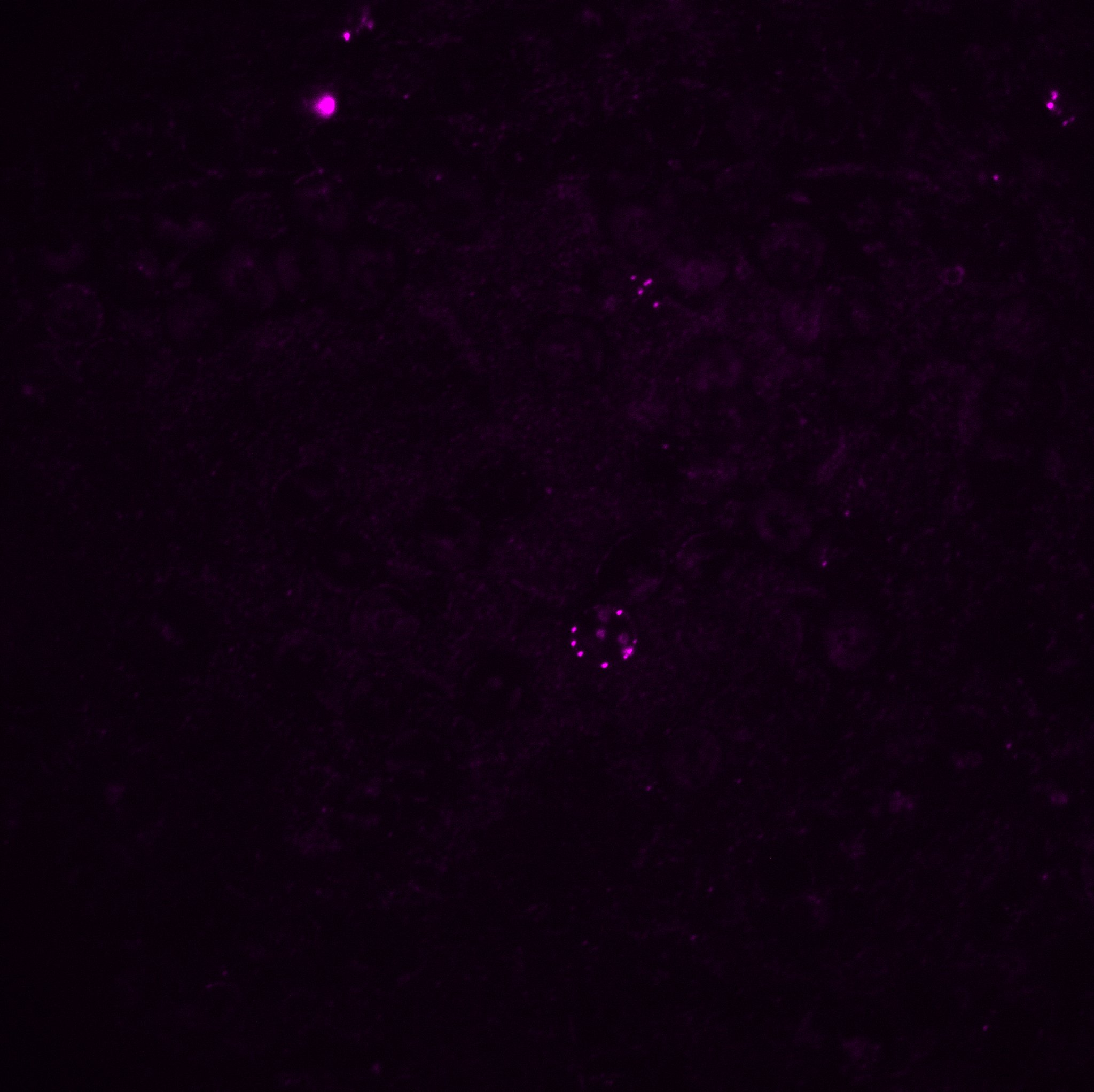

Supplement: Figure 3—figure supplement 4—source data 1. — Raw microscopy images of transgenic HSP70x-3xHA parasites probed with α-HA and α-SBP1. [file elife-107860-fig3-figsupp4-data1.zip › Figure 3 - Supplement 4 - Source Data 1 Raw Images/DMSO_HA_SBP1006-0001.jpg]

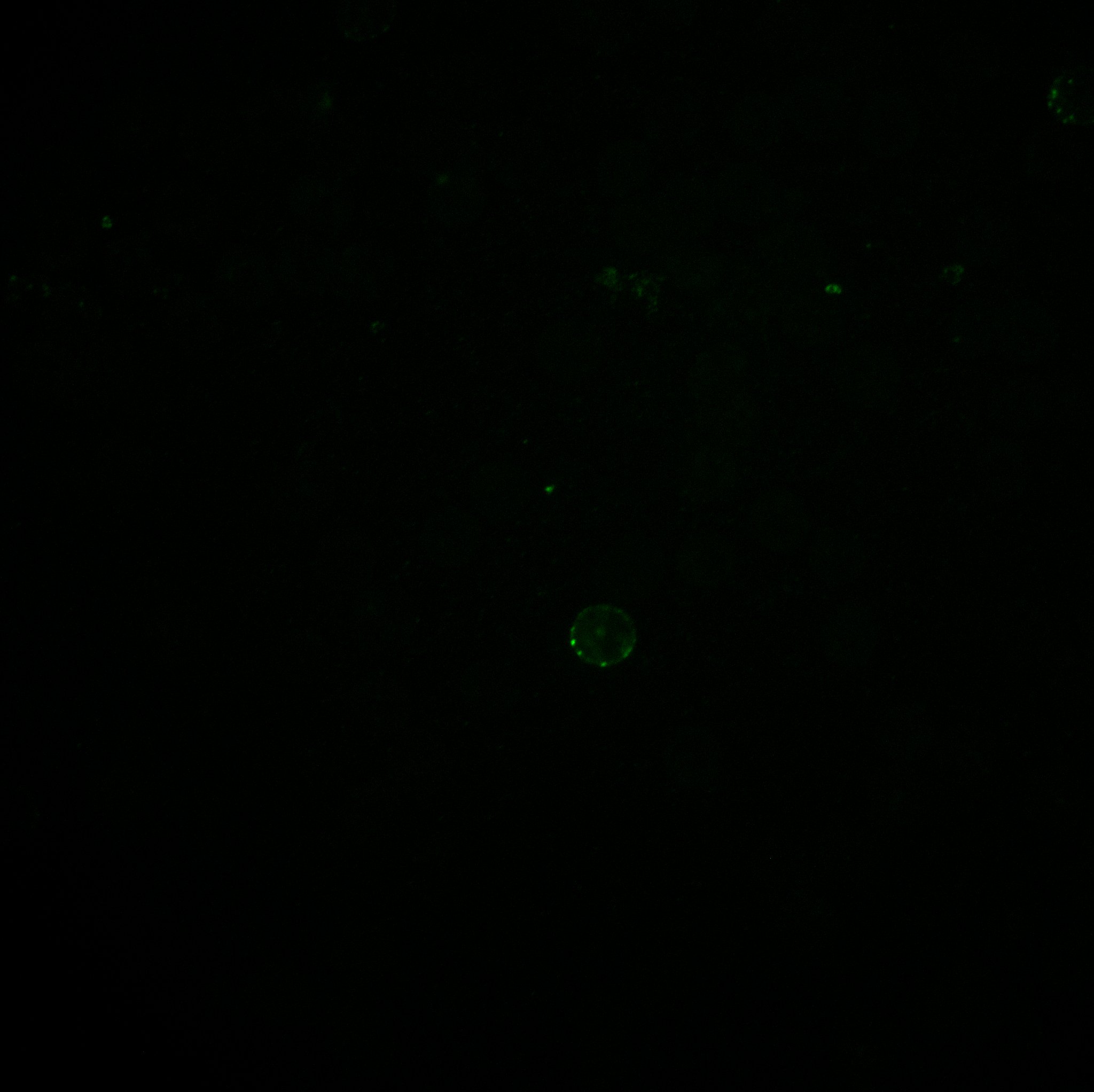

Supplement: Figure 3—figure supplement 4—source data 1. — Raw microscopy images of transgenic HSP70x-3xHA parasites probed with α-HA and α-SBP1. [file elife-107860-fig3-figsupp4-data1.zip › Figure 3 - Supplement 4 - Source Data 1 Raw Images/DMSO_HA_SBP1006-0002.jpg]

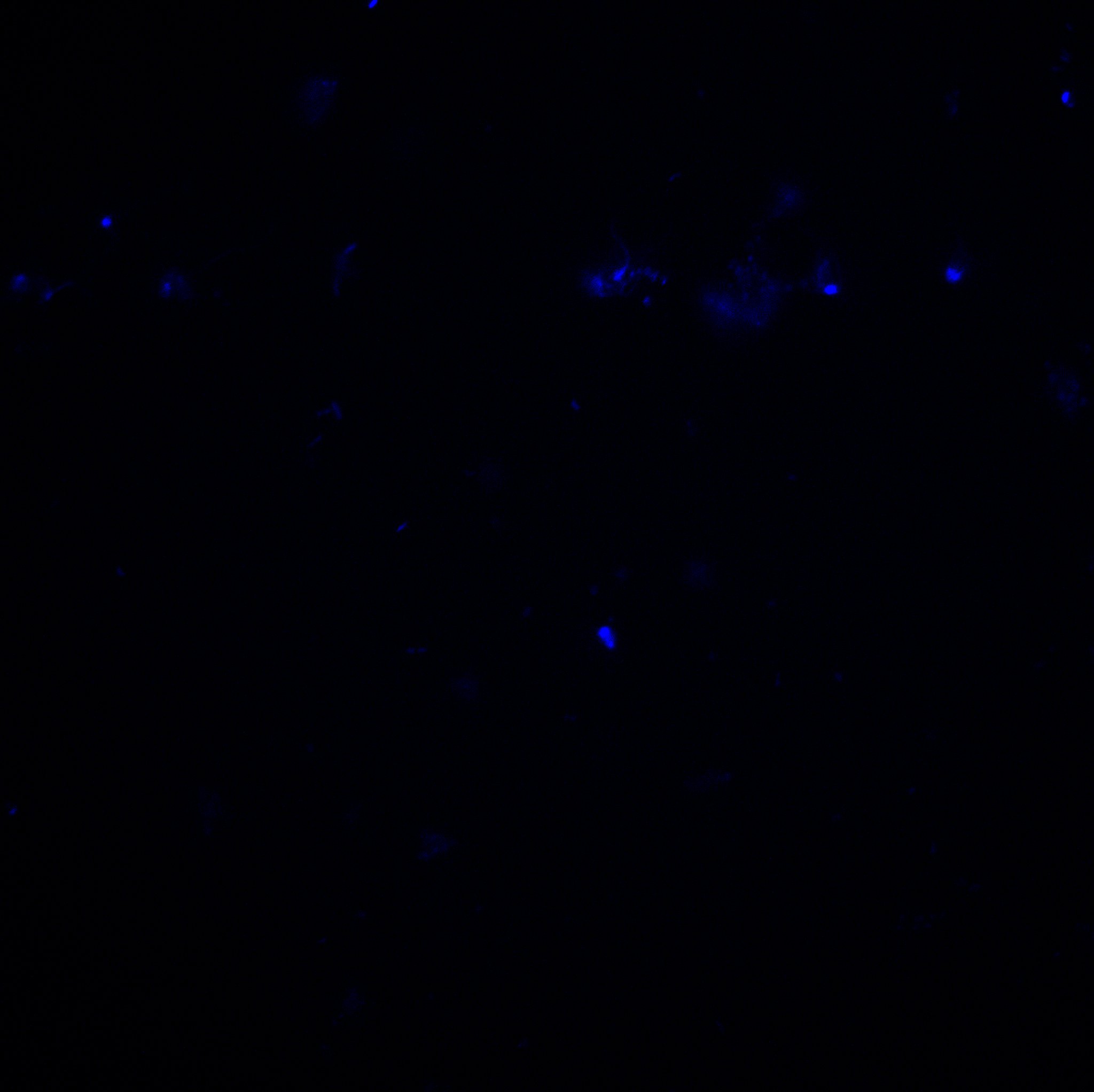

Supplement: Figure 3—figure supplement 4—source data 1. — Raw microscopy images of transgenic HSP70x-3xHA parasites probed with α-HA and α-SBP1. [file elife-107860-fig3-figsupp4-data1.zip › Figure 3 - Supplement 4 - Source Data 1 Raw Images/DMSO_HA_SBP1006-0003.jpg]

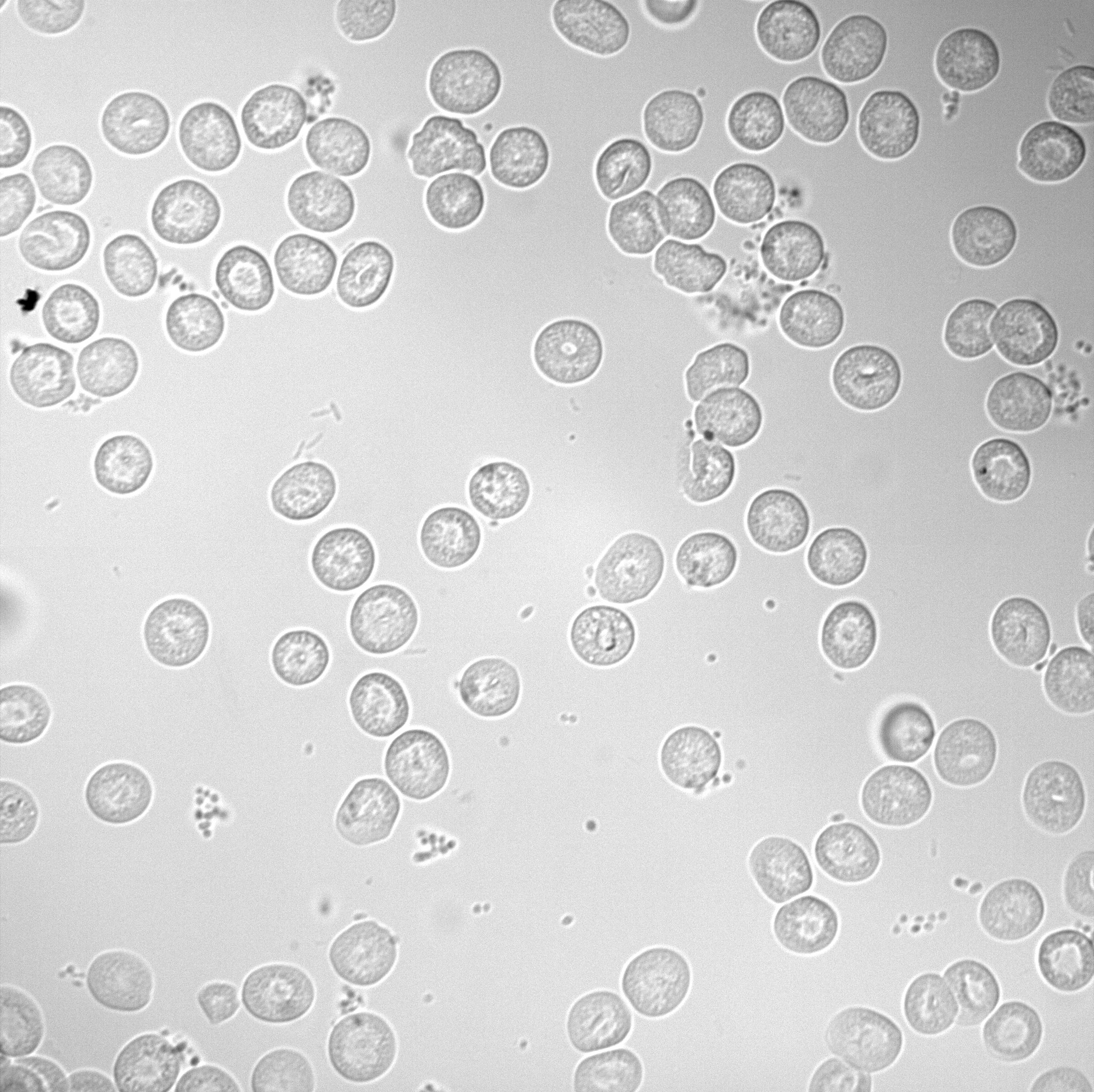

Supplement: Figure 3—figure supplement 4—source data 1. — Raw microscopy images of transgenic HSP70x-3xHA parasites probed with α-HA and α-SBP1. [file elife-107860-fig3-figsupp4-data1.zip › Figure 3 - Supplement 4 - Source Data 1 Raw Images/DMSO_HA_SBP1006-0004.jpg]

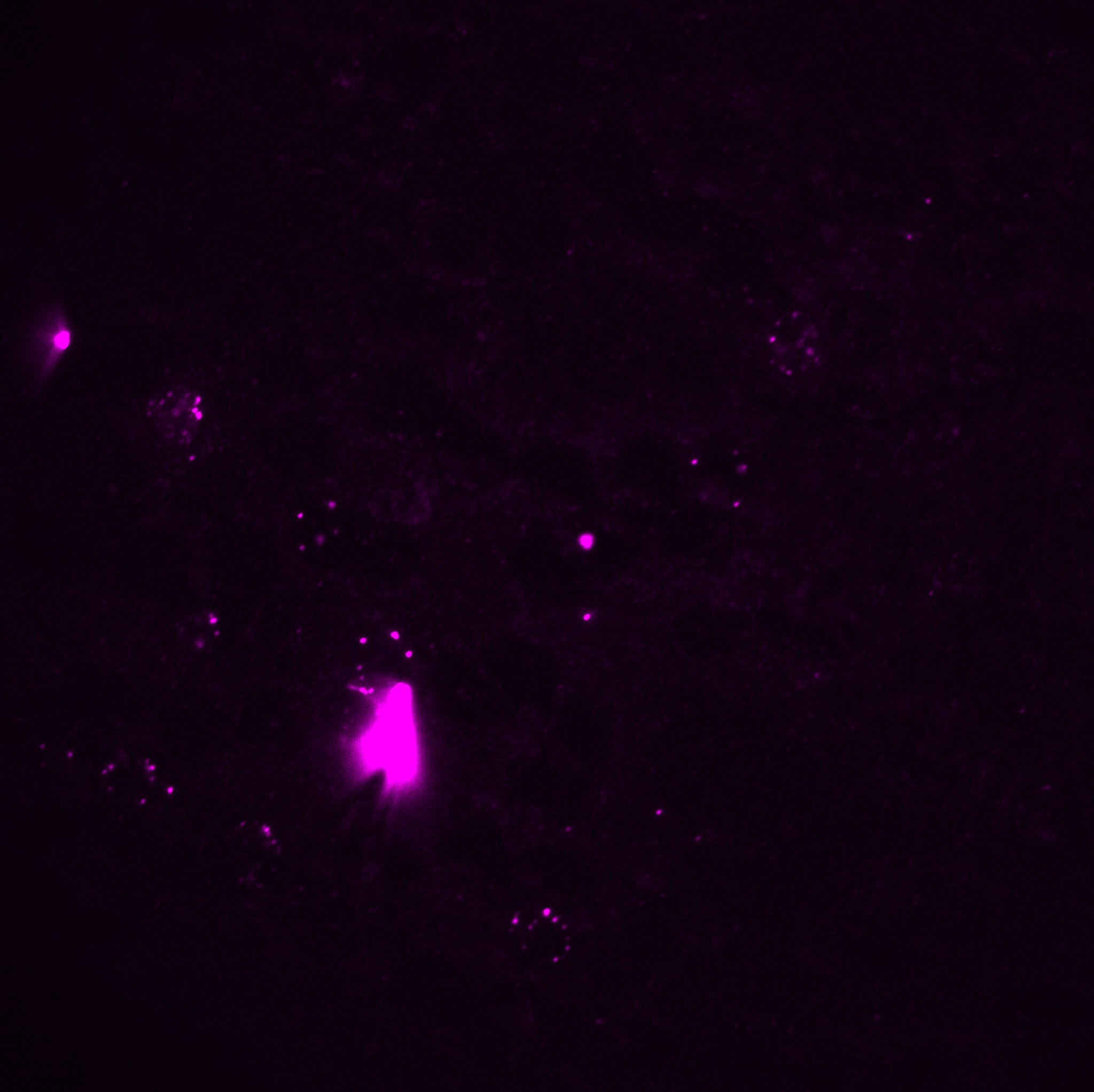

Supplement: Figure 3—figure supplement 4—source data 1. — Raw microscopy images of transgenic HSP70x-3xHA parasites probed with α-HA and α-SBP1. [file elife-107860-fig3-figsupp4-data1.zip › Figure 3 - Supplement 4 - Source Data 1 Raw Images/DMSO_HA_SBP1008-0001.jpg]

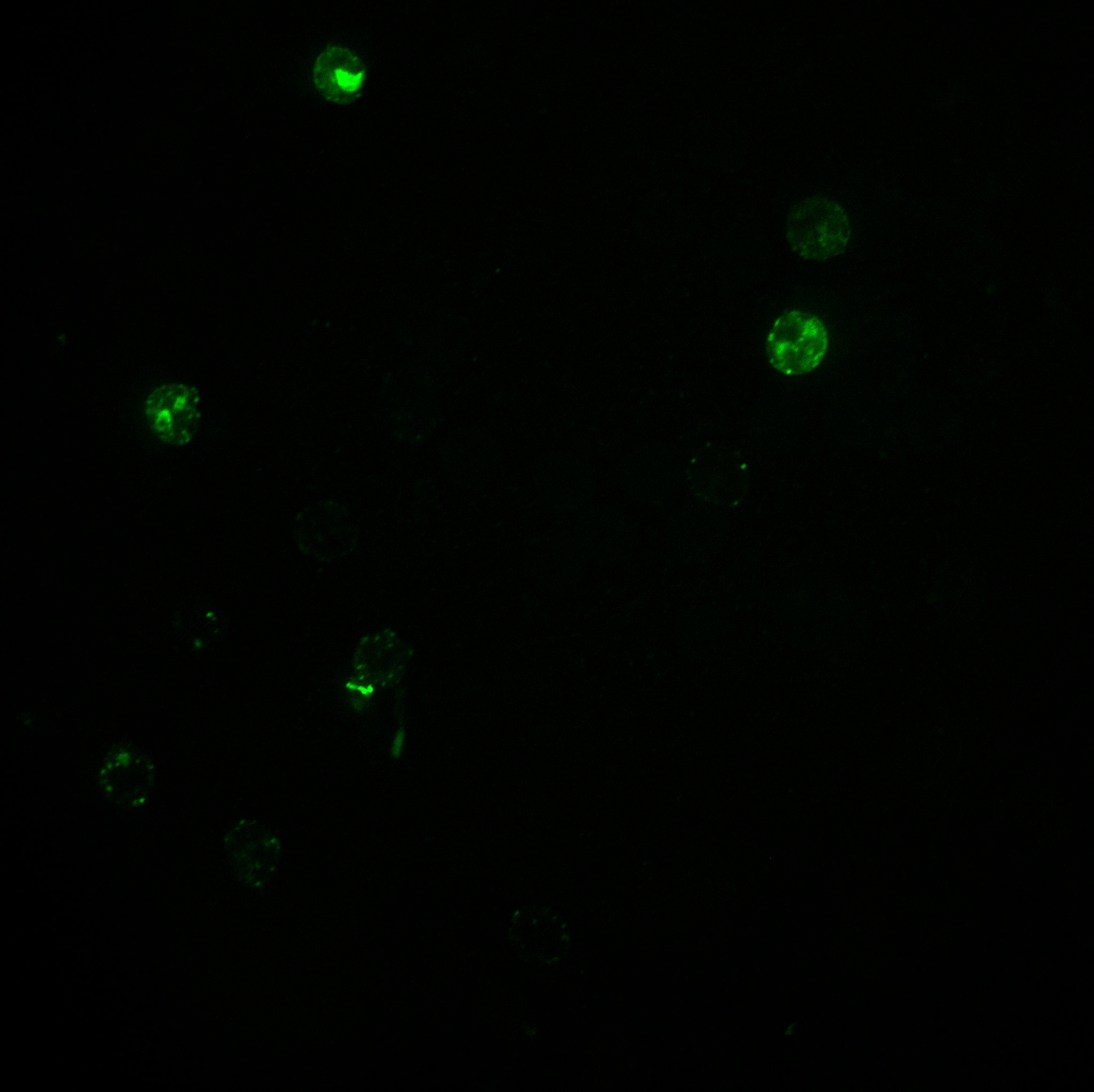

Supplement: Figure 3—figure supplement 4—source data 1. — Raw microscopy images of transgenic HSP70x-3xHA parasites probed with α-HA and α-SBP1. [file elife-107860-fig3-figsupp4-data1.zip › Figure 3 - Supplement 4 - Source Data 1 Raw Images/DMSO_HA_SBP1008-0002.jpg]

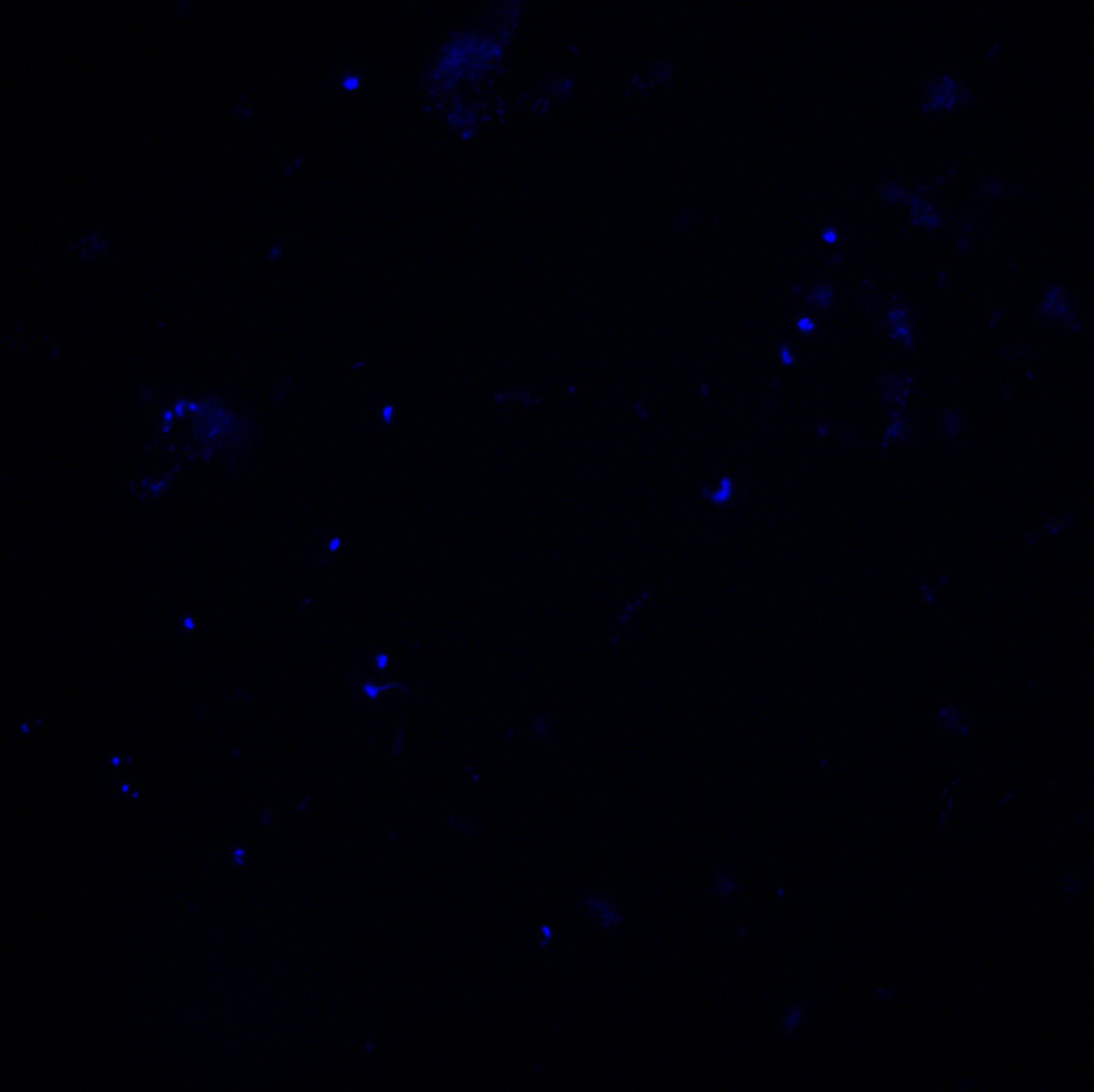

Supplement: Figure 3—figure supplement 4—source data 1. — Raw microscopy images of transgenic HSP70x-3xHA parasites probed with α-HA and α-SBP1. [file elife-107860-fig3-figsupp4-data1.zip › Figure 3 - Supplement 4 - Source Data 1 Raw Images/DMSO_HA_SBP1008-0003.jpg]

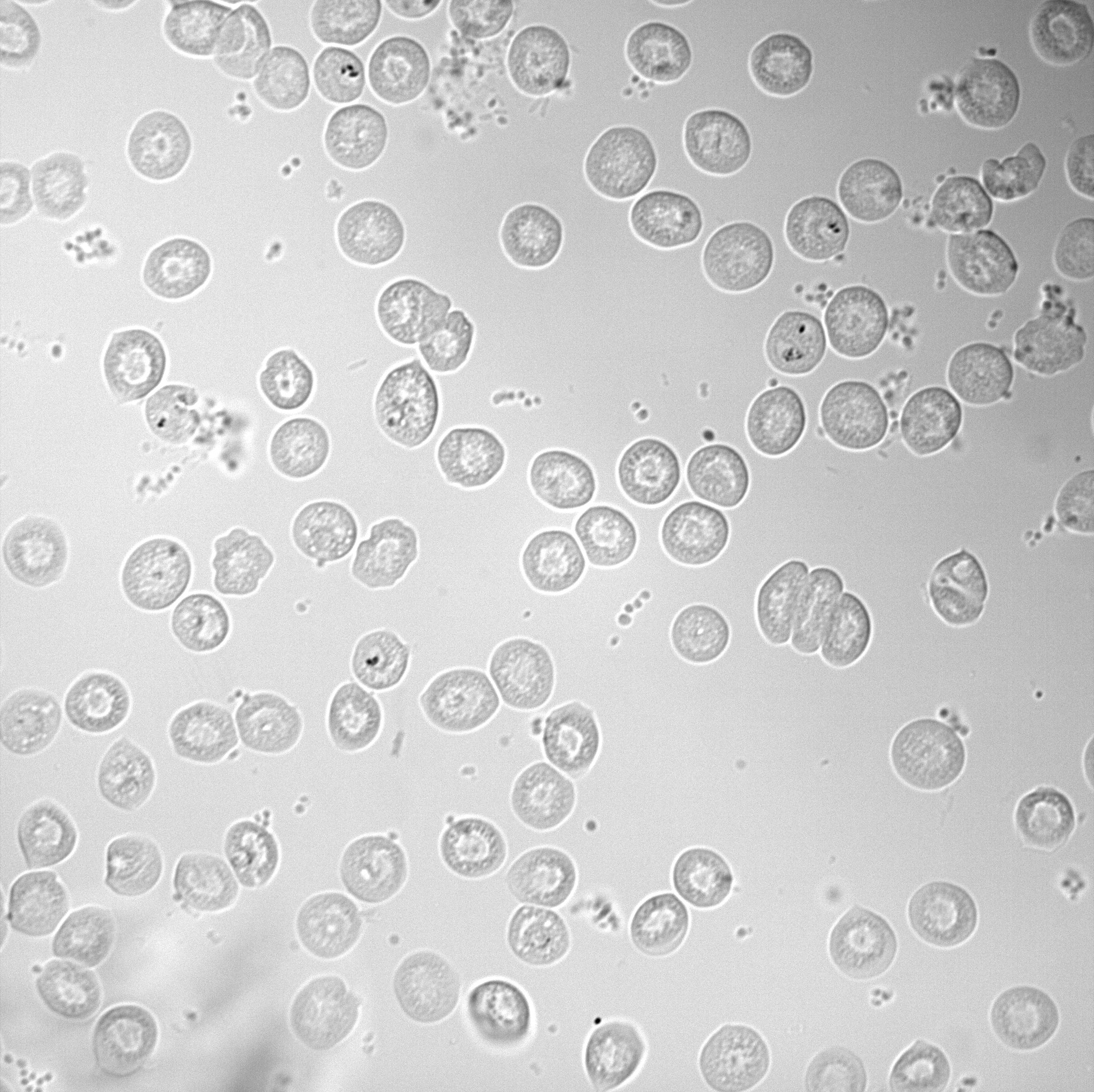

Supplement: Figure 3—figure supplement 4—source data 1. — Raw microscopy images of transgenic HSP70x-3xHA parasites probed with α-HA and α-SBP1. [file elife-107860-fig3-figsupp4-data1.zip › Figure 3 - Supplement 4 - Source Data 1 Raw Images/DMSO_HA_SBP1008-0004.jpg]

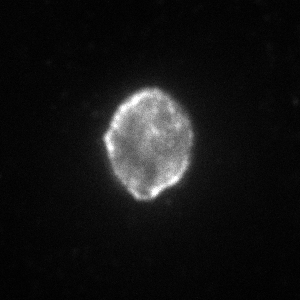

Supplement: Figure 3—figure supplement 4—source data 2. — Raw microscopy images of transgenic HSP70x-3xHA parasites probed with α-HA and α-KAHRP. [file elife-107860-fig3-figsupp4-data2.zip › Figure 3 - Supplement 4 - Source Data 2 Raw Images/Cropped/DMSO_HA_KAHRP007.nd2 - T=0-1.tif]

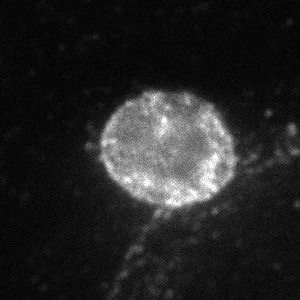

Supplement: Figure 3—figure supplement 4—source data 2. — Raw microscopy images of transgenic HSP70x-3xHA parasites probed with α-HA and α-KAHRP. [file elife-107860-fig3-figsupp4-data2.zip › Figure 3 - Supplement 4 - Source Data 2 Raw Images/Cropped/DMSO_HA_KAHRP008.nd2 - T=0-1.tif]

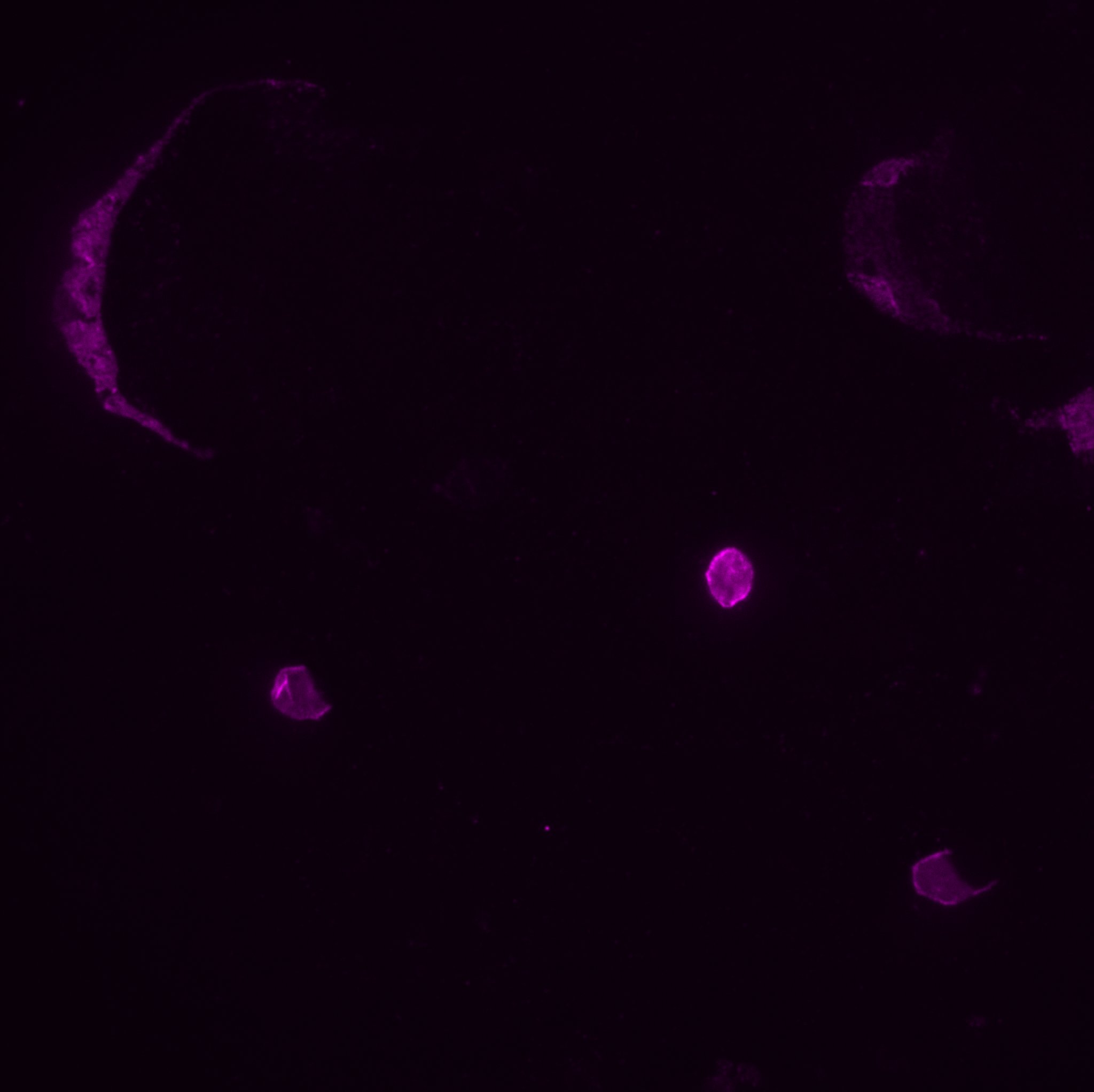

Supplement: Figure 3—figure supplement 4—source data 2. — Raw microscopy images of transgenic HSP70x-3xHA parasites probed with α-HA and α-KAHRP. [file elife-107860-fig3-figsupp4-data2.zip › Figure 3 - Supplement 4 - Source Data 2 Raw Images/DMSO_HA_KAHRP007-0001.jpg]

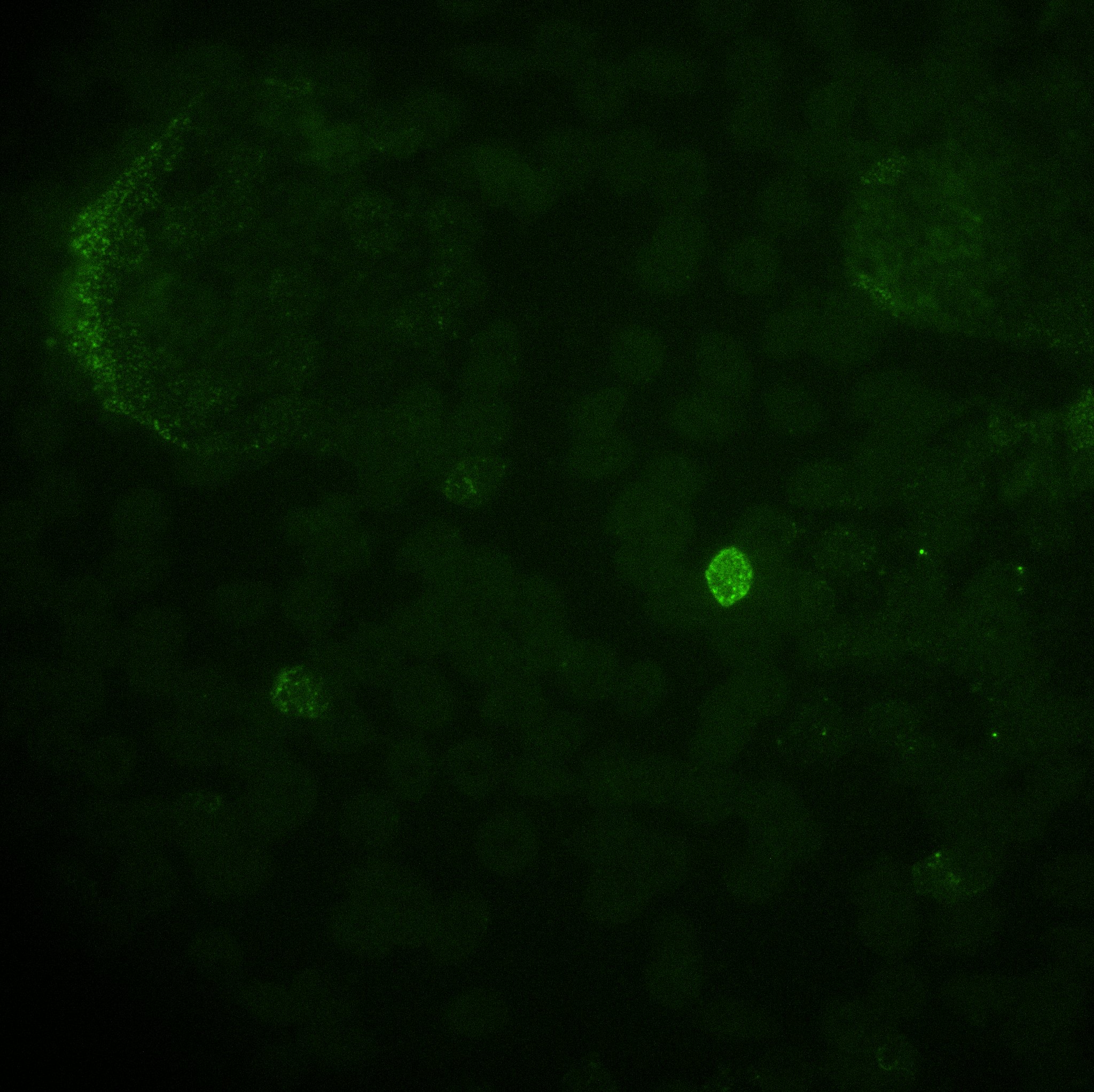

Supplement: Figure 3—figure supplement 4—source data 2. — Raw microscopy images of transgenic HSP70x-3xHA parasites probed with α-HA and α-KAHRP. [file elife-107860-fig3-figsupp4-data2.zip › Figure 3 - Supplement 4 - Source Data 2 Raw Images/DMSO_HA_KAHRP007-0002.jpg]

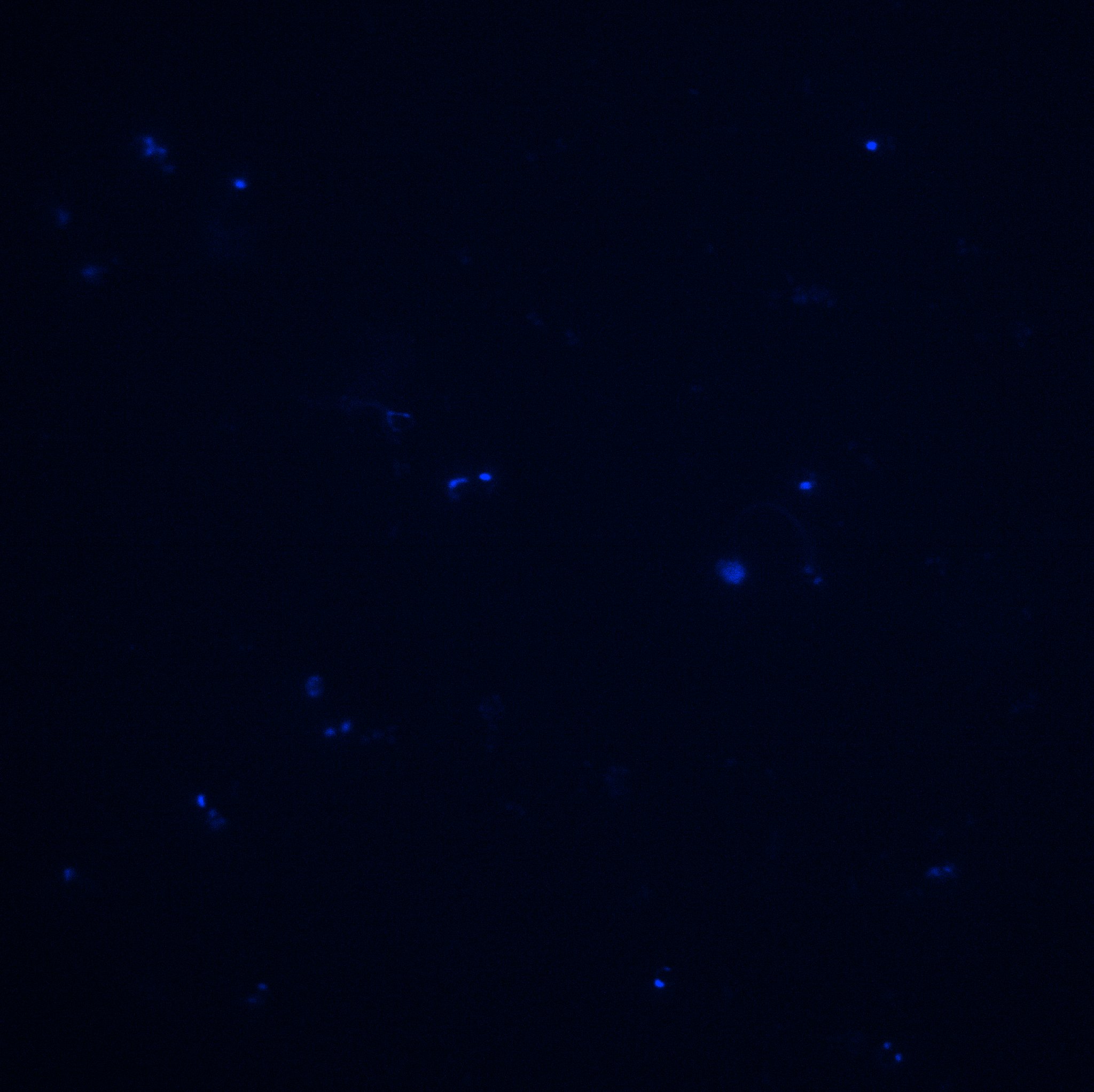

Supplement: Figure 3—figure supplement 4—source data 2. — Raw microscopy images of transgenic HSP70x-3xHA parasites probed with α-HA and α-KAHRP. [file elife-107860-fig3-figsupp4-data2.zip › Figure 3 - Supplement 4 - Source Data 2 Raw Images/DMSO_HA_KAHRP007-0003.jpg]

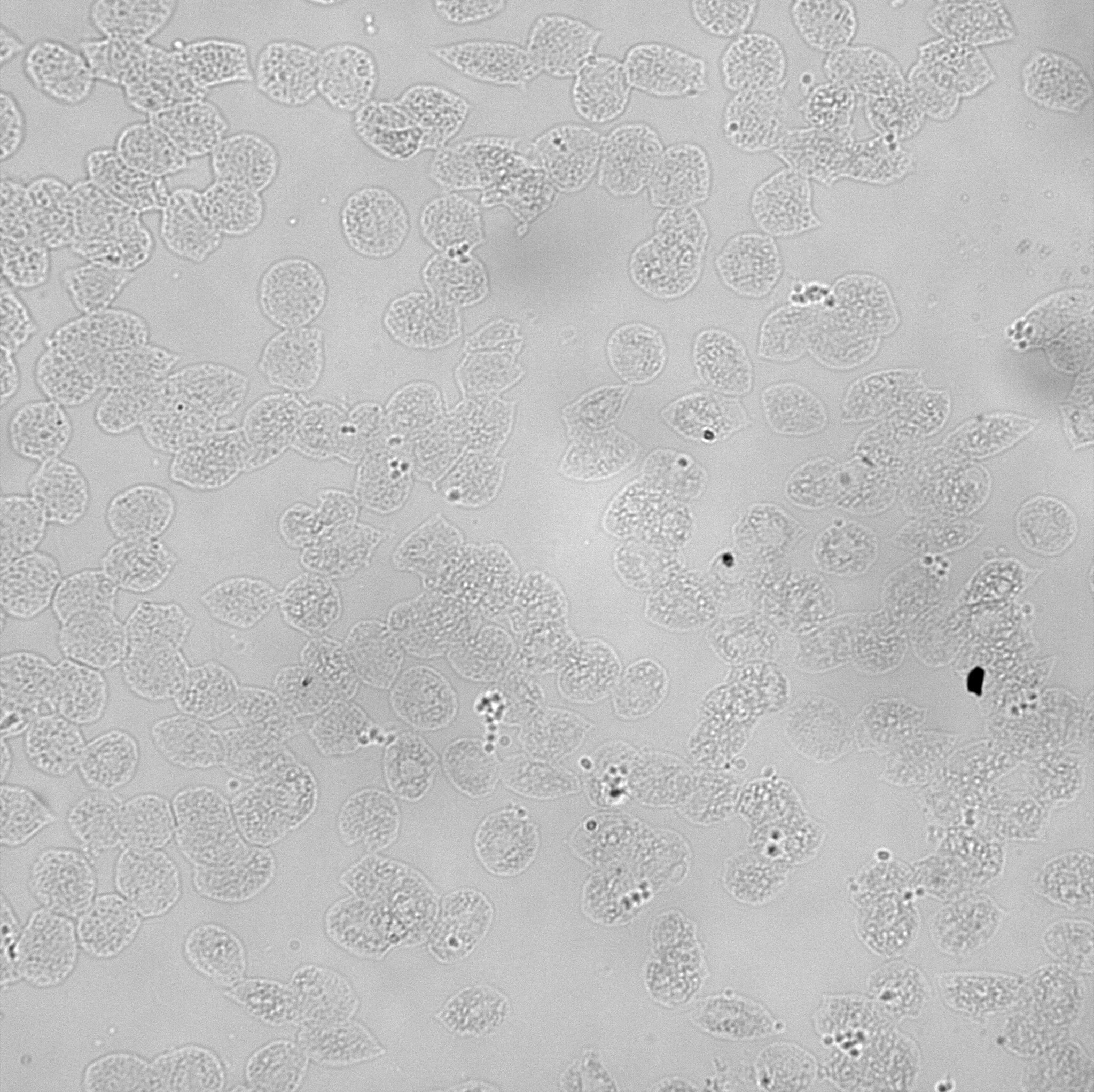

Supplement: Figure 3—figure supplement 4—source data 2. — Raw microscopy images of transgenic HSP70x-3xHA parasites probed with α-HA and α-KAHRP. [file elife-107860-fig3-figsupp4-data2.zip › Figure 3 - Supplement 4 - Source Data 2 Raw Images/DMSO_HA_KAHRP007-0004.jpg]

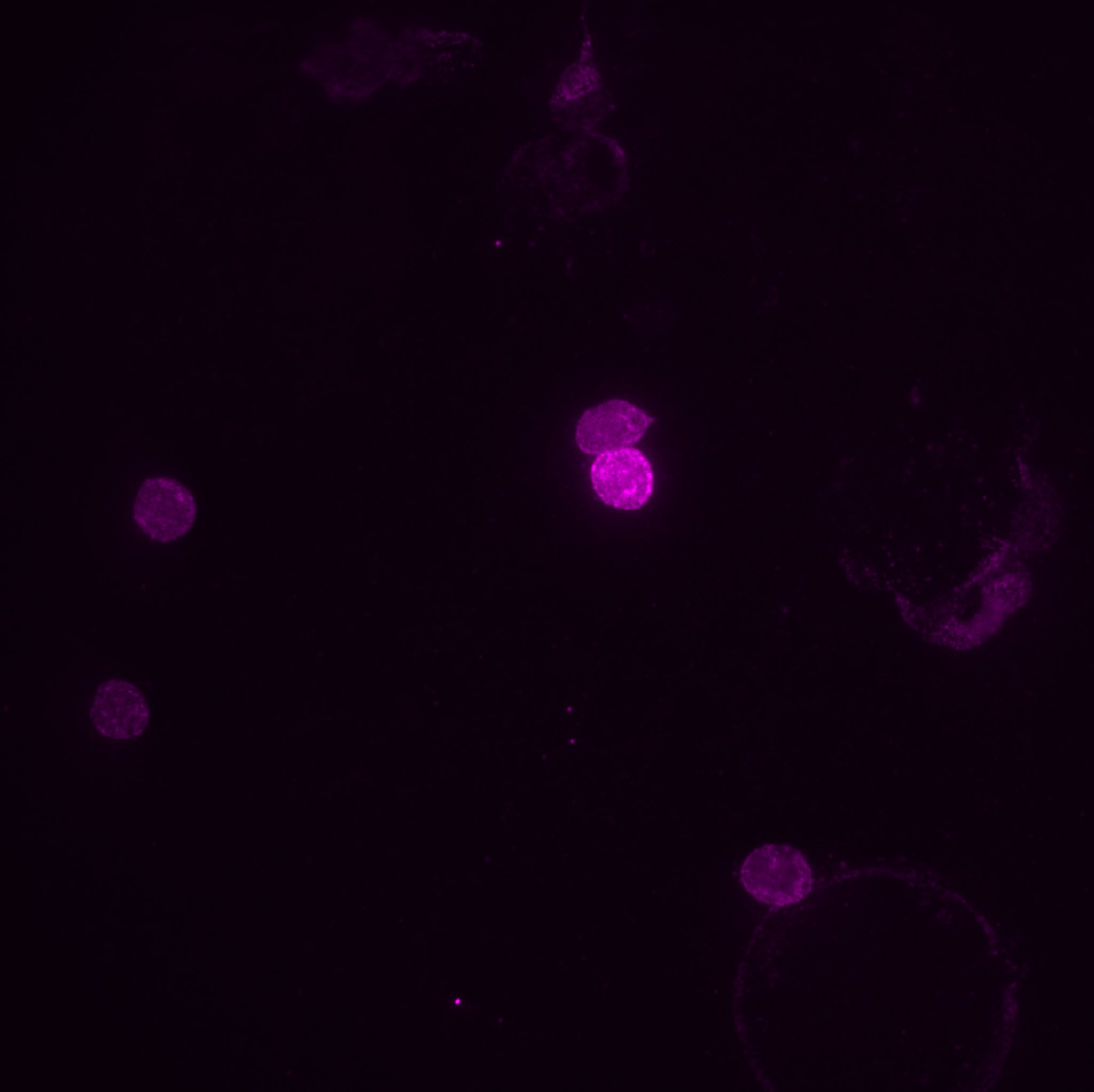

Supplement: Figure 3—figure supplement 4—source data 2. — Raw microscopy images of transgenic HSP70x-3xHA parasites probed with α-HA and α-KAHRP. [file elife-107860-fig3-figsupp4-data2.zip › Figure 3 - Supplement 4 - Source Data 2 Raw Images/DMSO_HA_KAHRP008-0001.jpg]

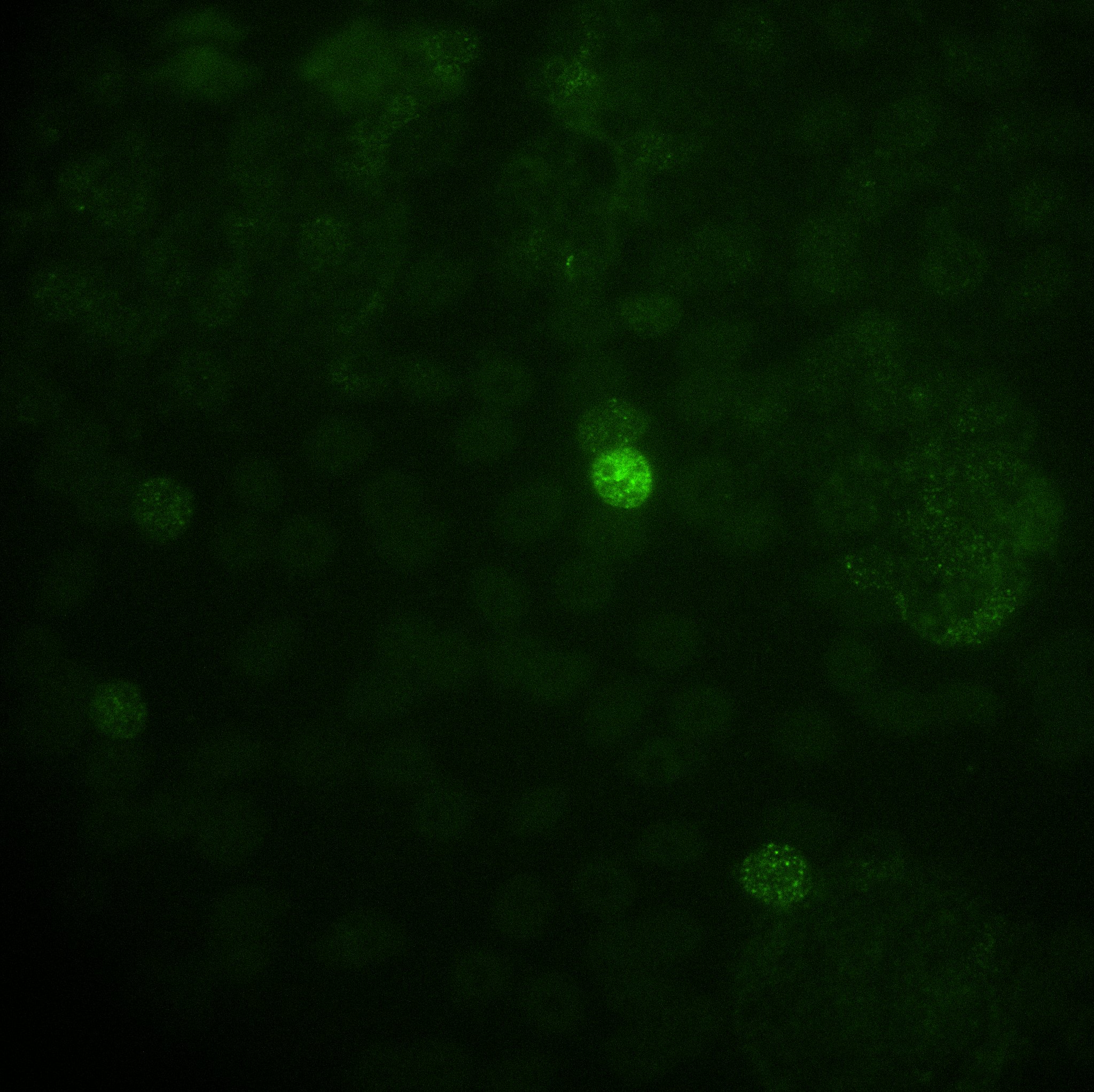

Supplement: Figure 3—figure supplement 4—source data 2. — Raw microscopy images of transgenic HSP70x-3xHA parasites probed with α-HA and α-KAHRP. [file elife-107860-fig3-figsupp4-data2.zip › Figure 3 - Supplement 4 - Source Data 2 Raw Images/DMSO_HA_KAHRP008-0002.jpg]

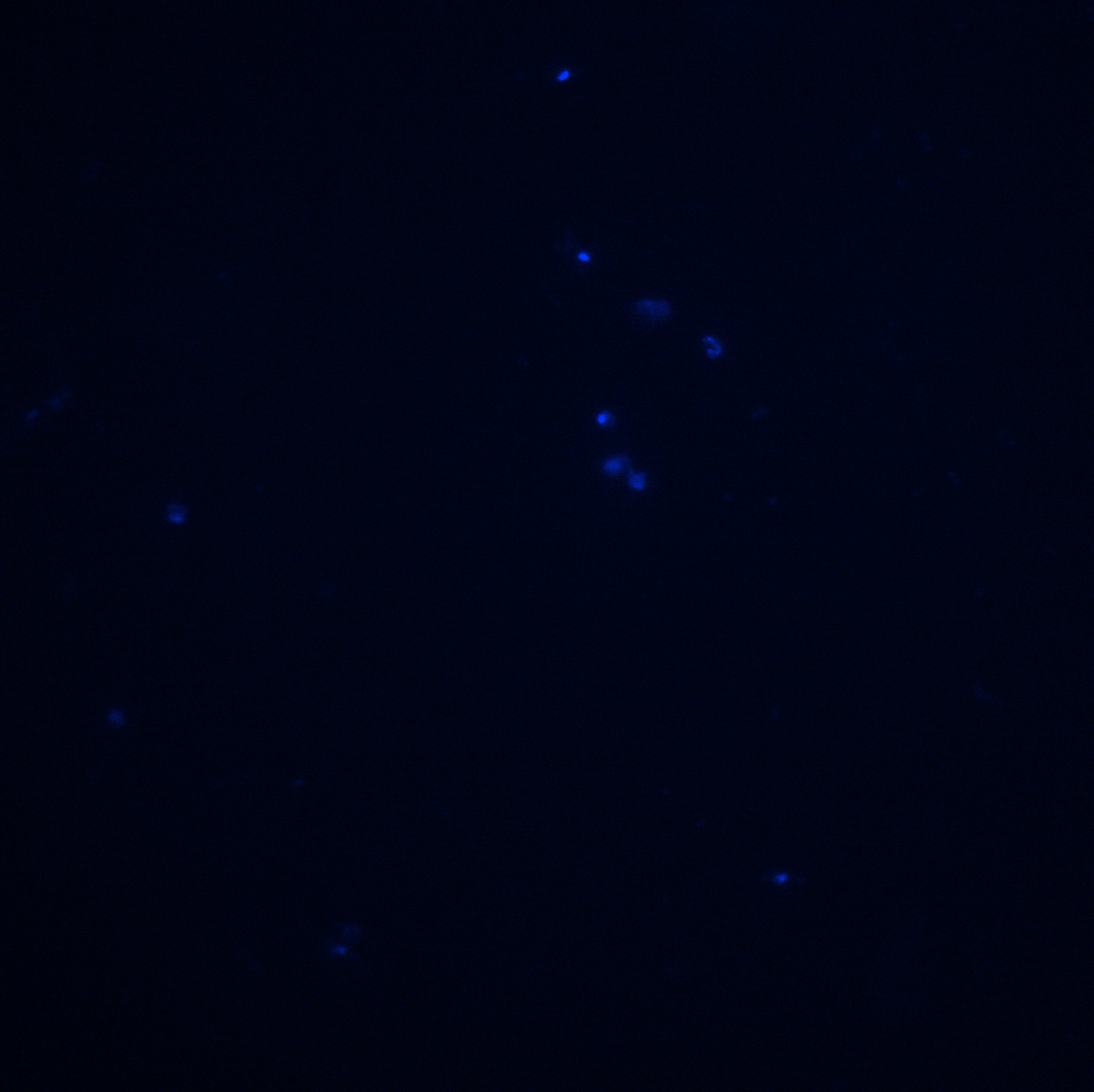

Supplement: Figure 3—figure supplement 4—source data 2. — Raw microscopy images of transgenic HSP70x-3xHA parasites probed with α-HA and α-KAHRP. [file elife-107860-fig3-figsupp4-data2.zip › Figure 3 - Supplement 4 - Source Data 2 Raw Images/DMSO_HA_KAHRP008-0003.jpg]

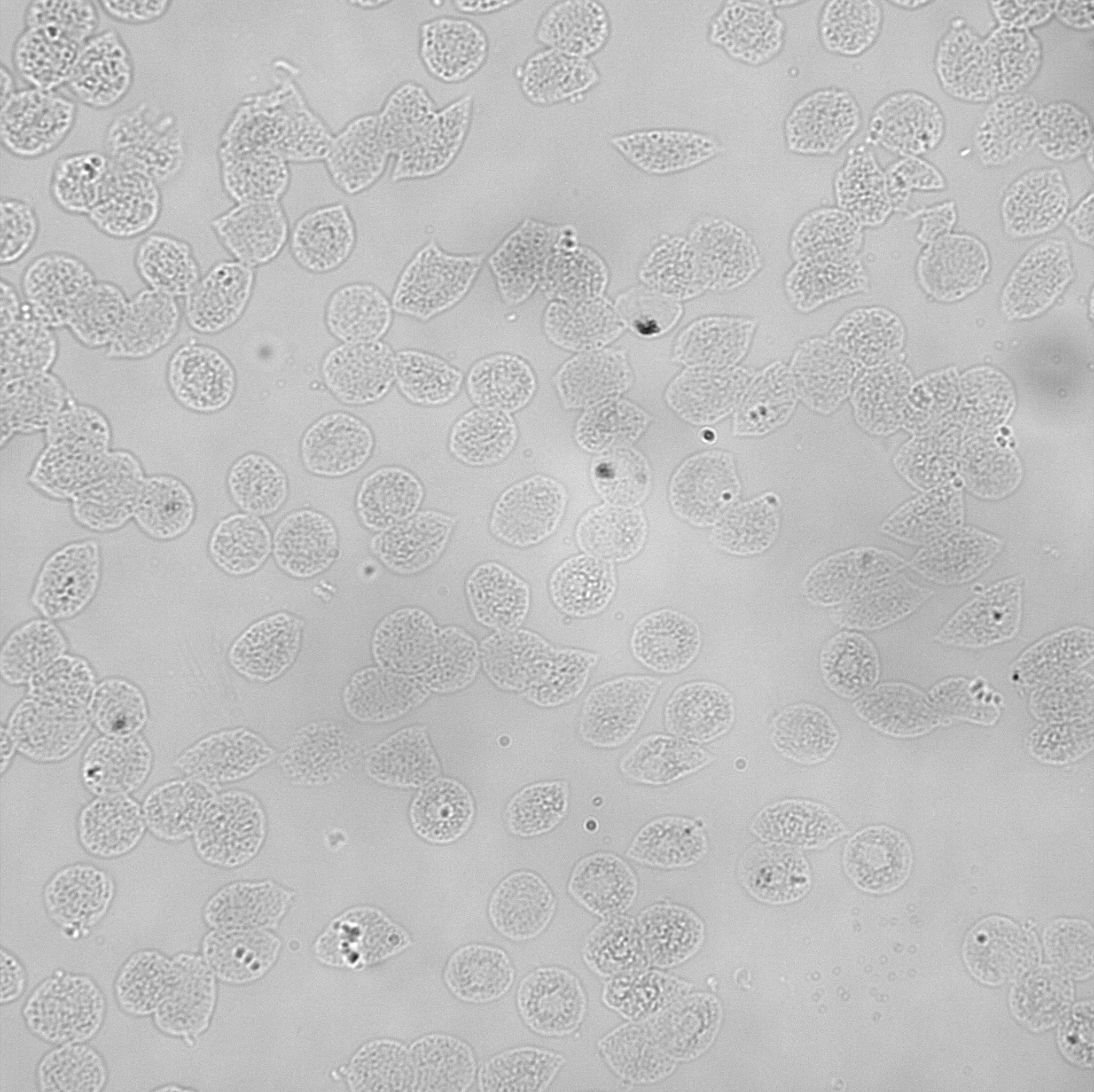

Supplement: Figure 3—figure supplement 4—source data 2. — Raw microscopy images of transgenic HSP70x-3xHA parasites probed with α-HA and α-KAHRP. [file elife-107860-fig3-figsupp4-data2.zip › Figure 3 - Supplement 4 - Source Data 2 Raw Images/DMSO_HA_KAHRP008-0004.jpg]

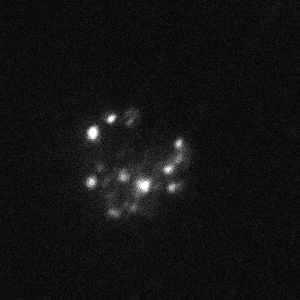

Supplement: Figure 3—figure supplement 6—source data 1. — Raw microscopy images of transgenic PF3D7_0702500-3xHA parasites probed with α-HA and α-SBP1. [file elife-107860-fig3-figsupp6-data1.zip › Figure 3 - Supplement 6 - Source Data 1 Raw Images/Cropped/HA_SBP1005.nd2 - T=0-1.tif]

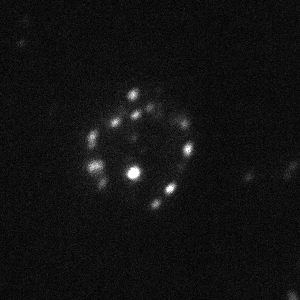

Supplement: Figure 3—figure supplement 6—source data 1. — Raw microscopy images of transgenic PF3D7_0702500-3xHA parasites probed with α-HA and α-SBP1. [file elife-107860-fig3-figsupp6-data1.zip › Figure 3 - Supplement 6 - Source Data 1 Raw Images/Cropped/HA_SBP1007.nd2 - T=0-1.tif]

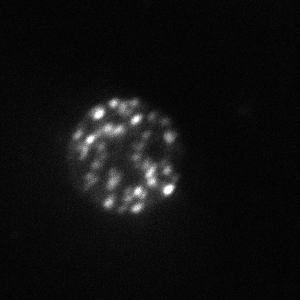

Supplement: Figure 3—figure supplement 6—source data 1. — Raw microscopy images of transgenic PF3D7_0702500-3xHA parasites probed with α-HA and α-SBP1. [file elife-107860-fig3-figsupp6-data1.zip › Figure 3 - Supplement 6 - Source Data 1 Raw Images/Cropped/HA_SBP1007_b.nd2 - T=0-1.tif]

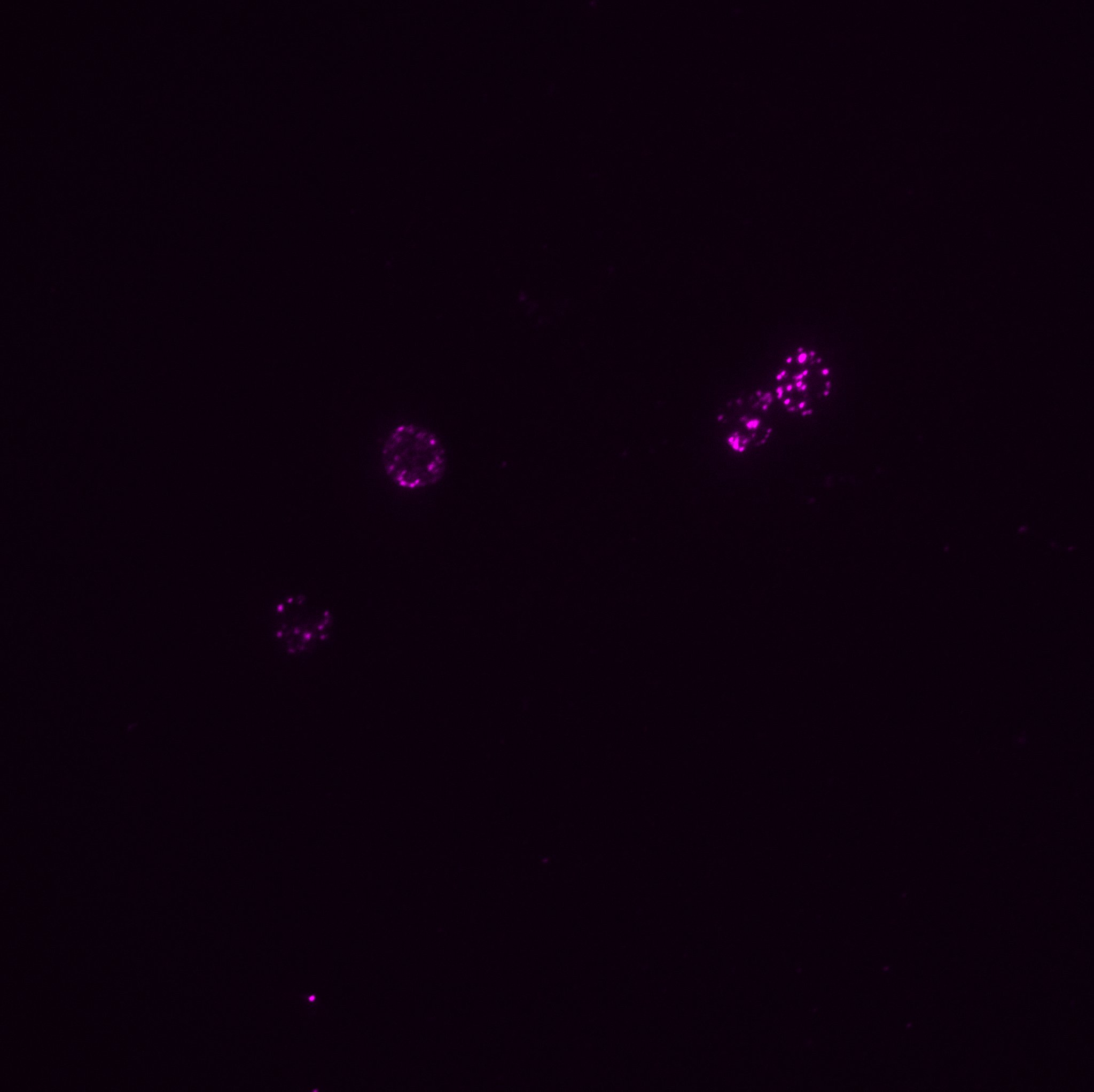

Supplement: Figure 3—figure supplement 6—source data 1. — Raw microscopy images of transgenic PF3D7_0702500-3xHA parasites probed with α-HA and α-SBP1. [file elife-107860-fig3-figsupp6-data1.zip › Figure 3 - Supplement 6 - Source Data 1 Raw Images/HA_SBP1005-0001.jpg]

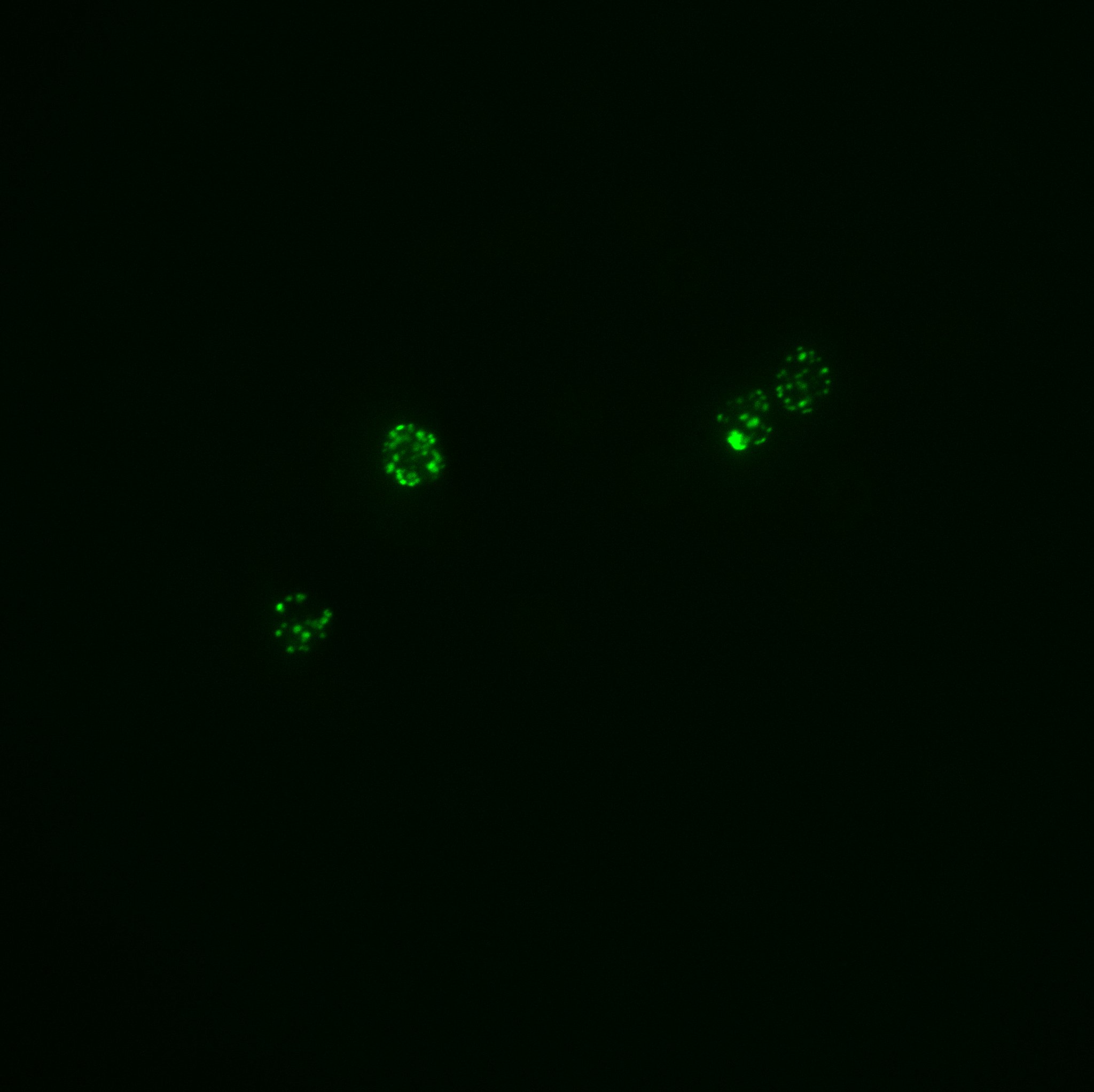

Supplement: Figure 3—figure supplement 6—source data 1. — Raw microscopy images of transgenic PF3D7_0702500-3xHA parasites probed with α-HA and α-SBP1. [file elife-107860-fig3-figsupp6-data1.zip › Figure 3 - Supplement 6 - Source Data 1 Raw Images/HA_SBP1005-0002.jpg]

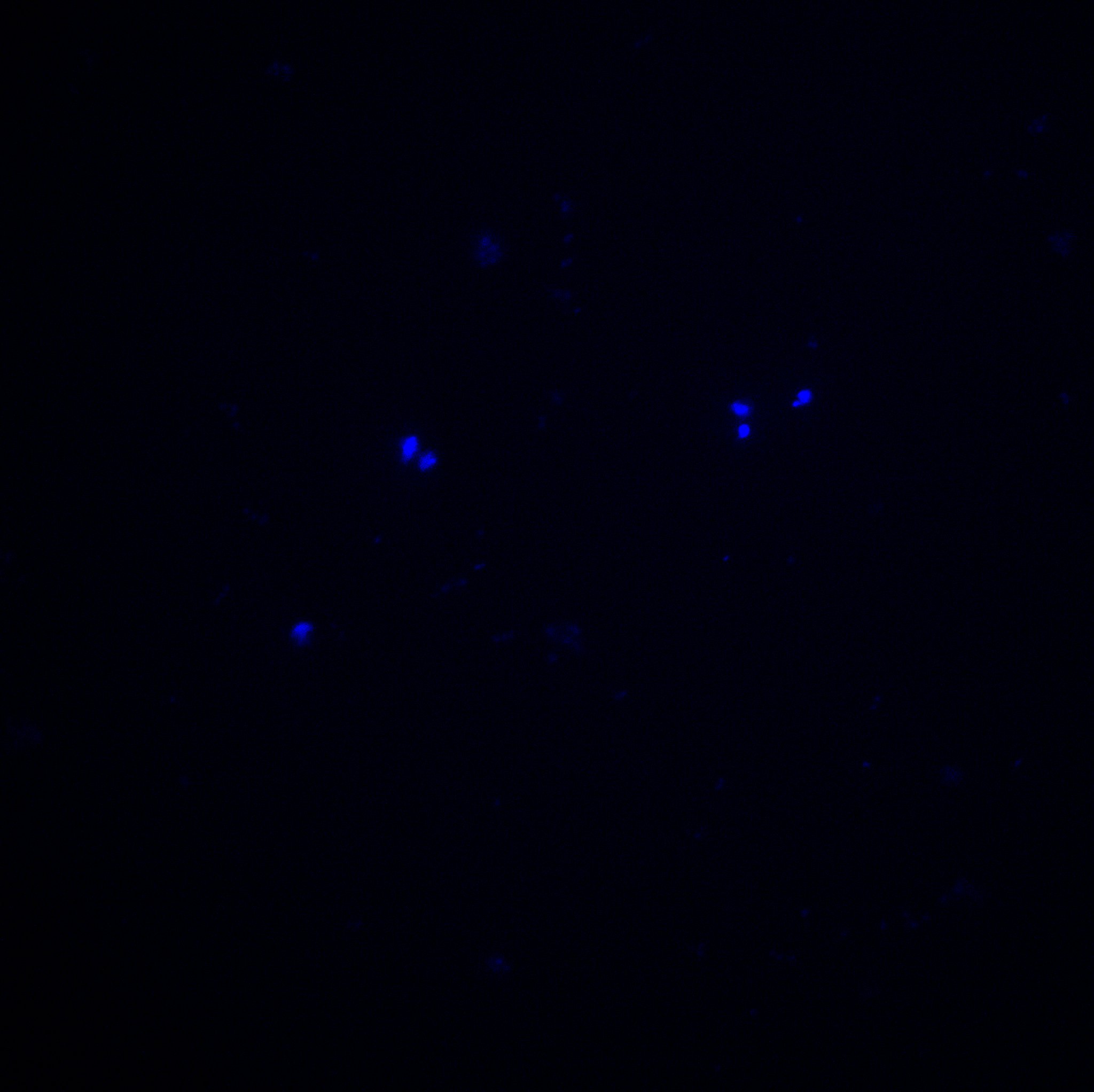

Supplement: Figure 3—figure supplement 6—source data 1. — Raw microscopy images of transgenic PF3D7_0702500-3xHA parasites probed with α-HA and α-SBP1. [file elife-107860-fig3-figsupp6-data1.zip › Figure 3 - Supplement 6 - Source Data 1 Raw Images/HA_SBP1005-0003.jpg]

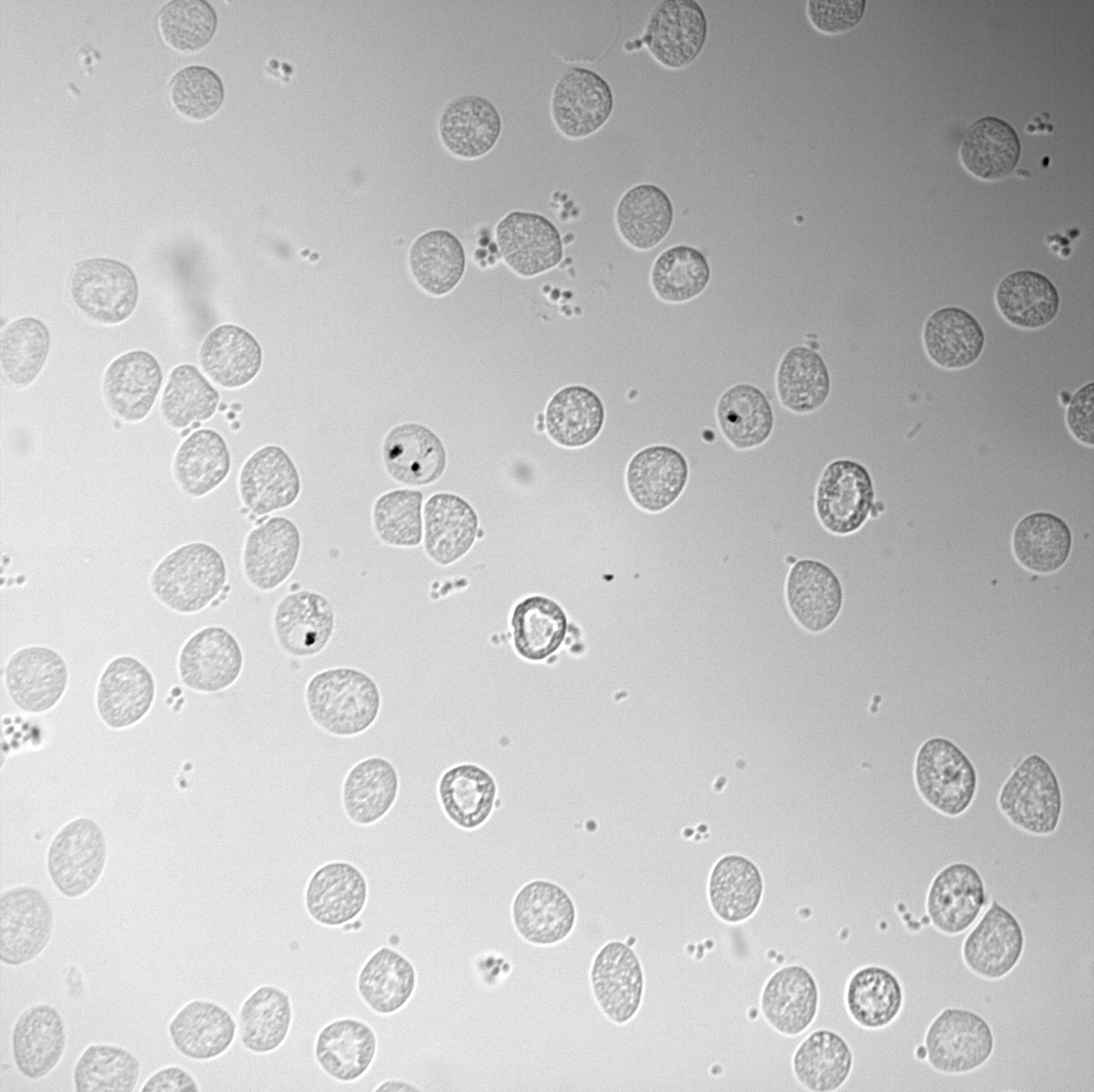

Supplement: Figure 3—figure supplement 6—source data 1. — Raw microscopy images of transgenic PF3D7_0702500-3xHA parasites probed with α-HA and α-SBP1. [file elife-107860-fig3-figsupp6-data1.zip › Figure 3 - Supplement 6 - Source Data 1 Raw Images/HA_SBP1005-0004.jpg]

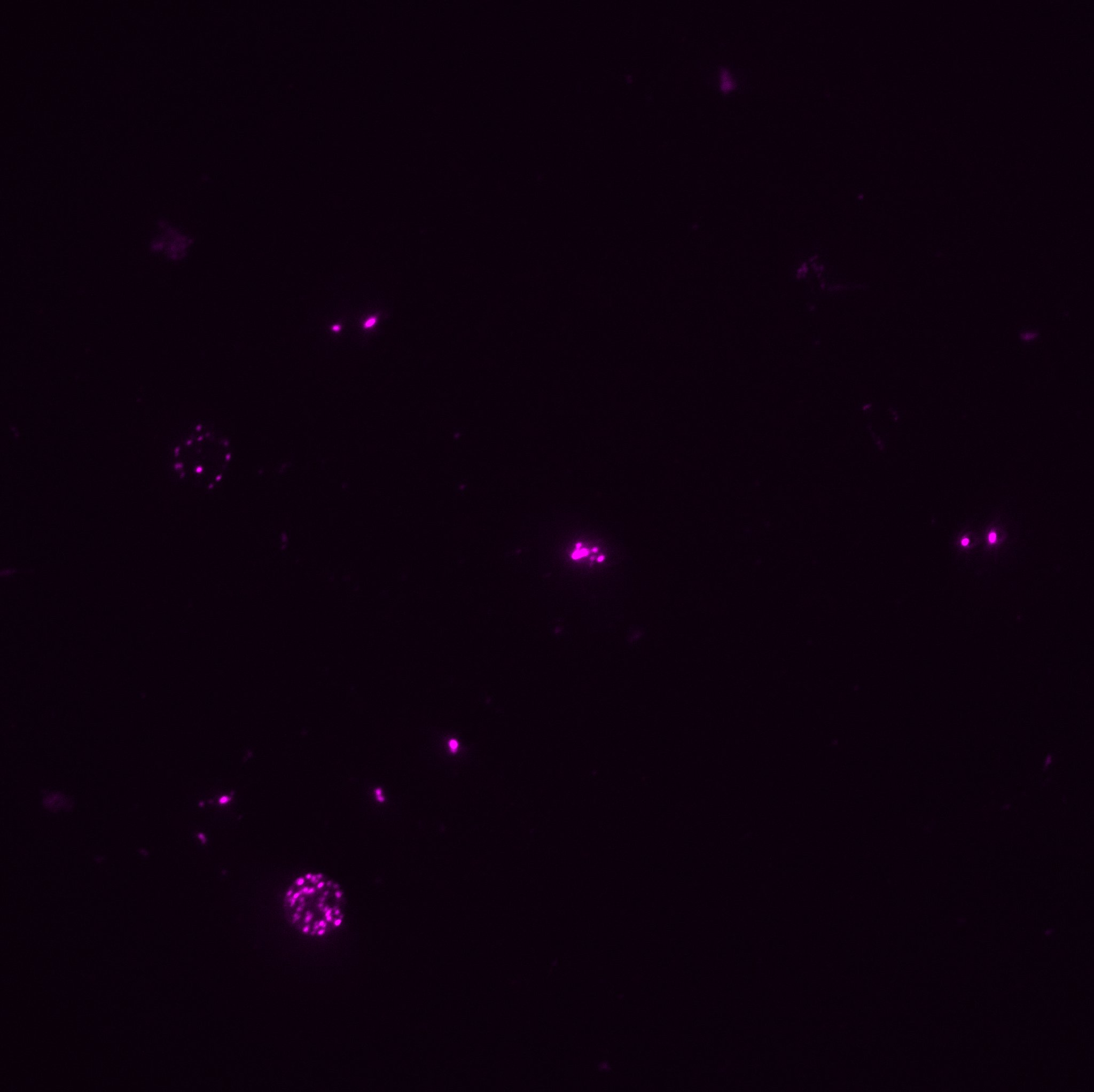

Supplement: Figure 3—figure supplement 6—source data 1. — Raw microscopy images of transgenic PF3D7_0702500-3xHA parasites probed with α-HA and α-SBP1. [file elife-107860-fig3-figsupp6-data1.zip › Figure 3 - Supplement 6 - Source Data 1 Raw Images/HA_SBP1007-0001.jpg]

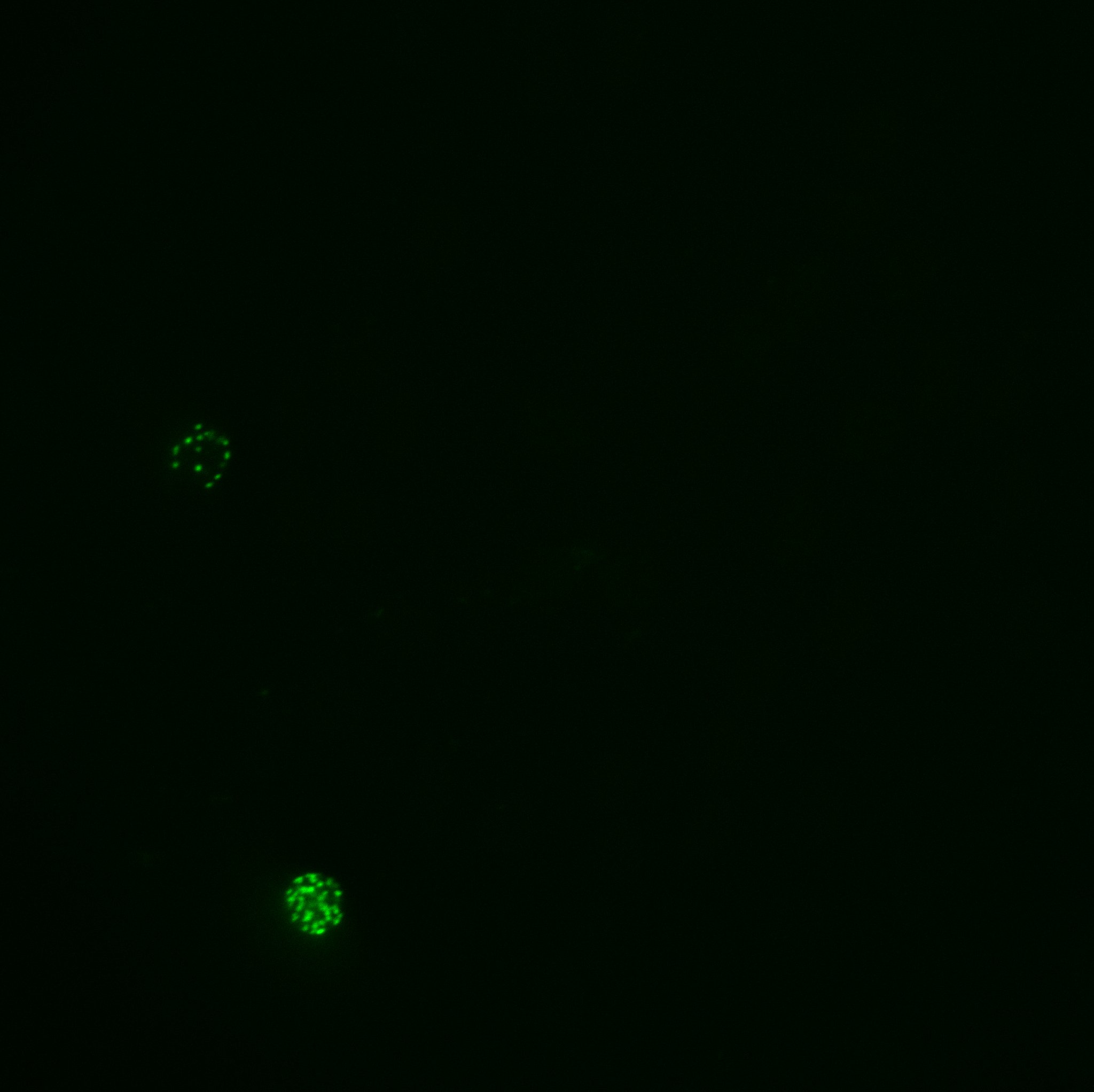

Supplement: Figure 3—figure supplement 6—source data 1. — Raw microscopy images of transgenic PF3D7_0702500-3xHA parasites probed with α-HA and α-SBP1. [file elife-107860-fig3-figsupp6-data1.zip › Figure 3 - Supplement 6 - Source Data 1 Raw Images/HA_SBP1007-0002.jpg]

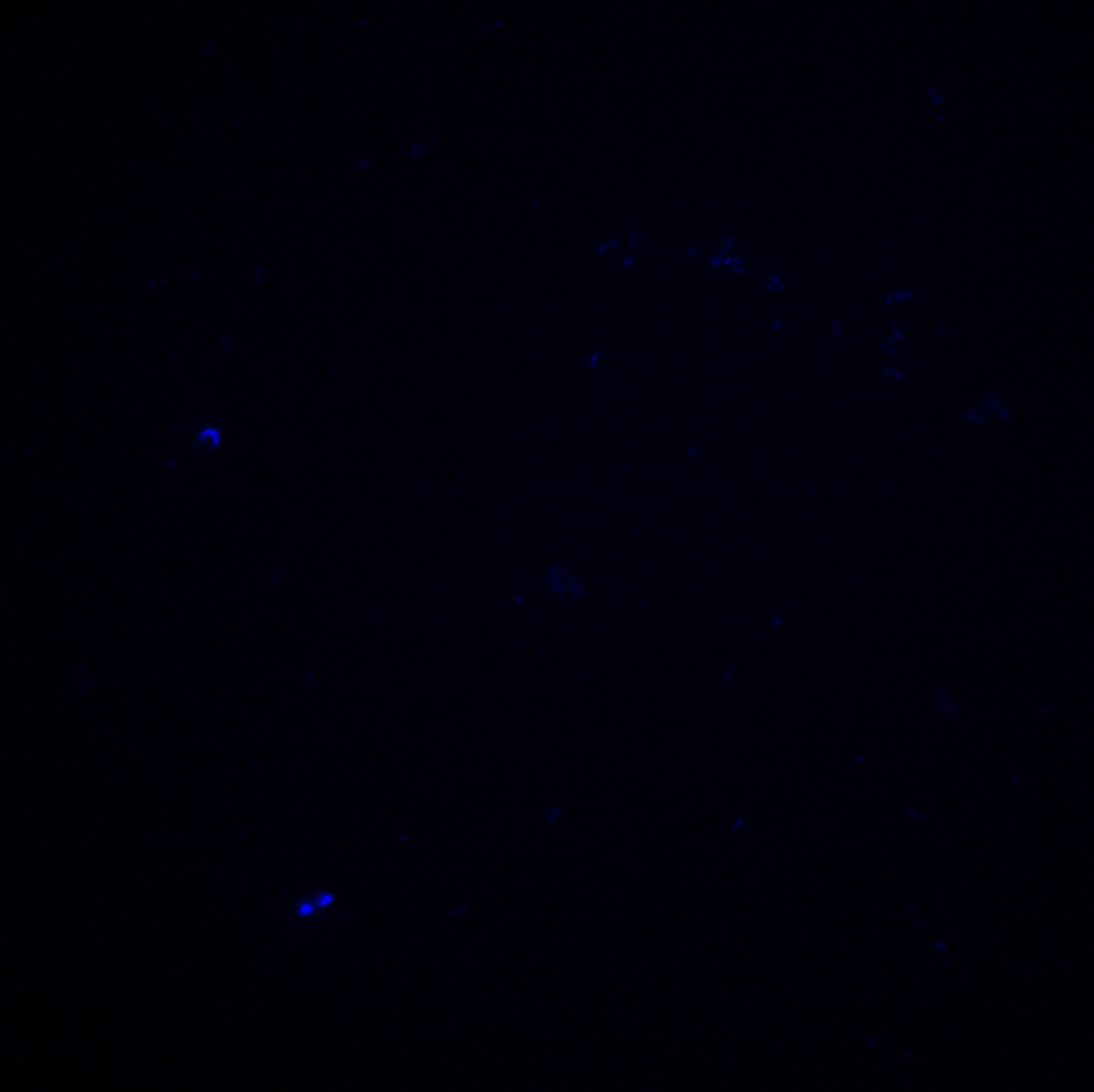

Supplement: Figure 3—figure supplement 6—source data 1. — Raw microscopy images of transgenic PF3D7_0702500-3xHA parasites probed with α-HA and α-SBP1. [file elife-107860-fig3-figsupp6-data1.zip › Figure 3 - Supplement 6 - Source Data 1 Raw Images/HA_SBP1007-0003.jpg]

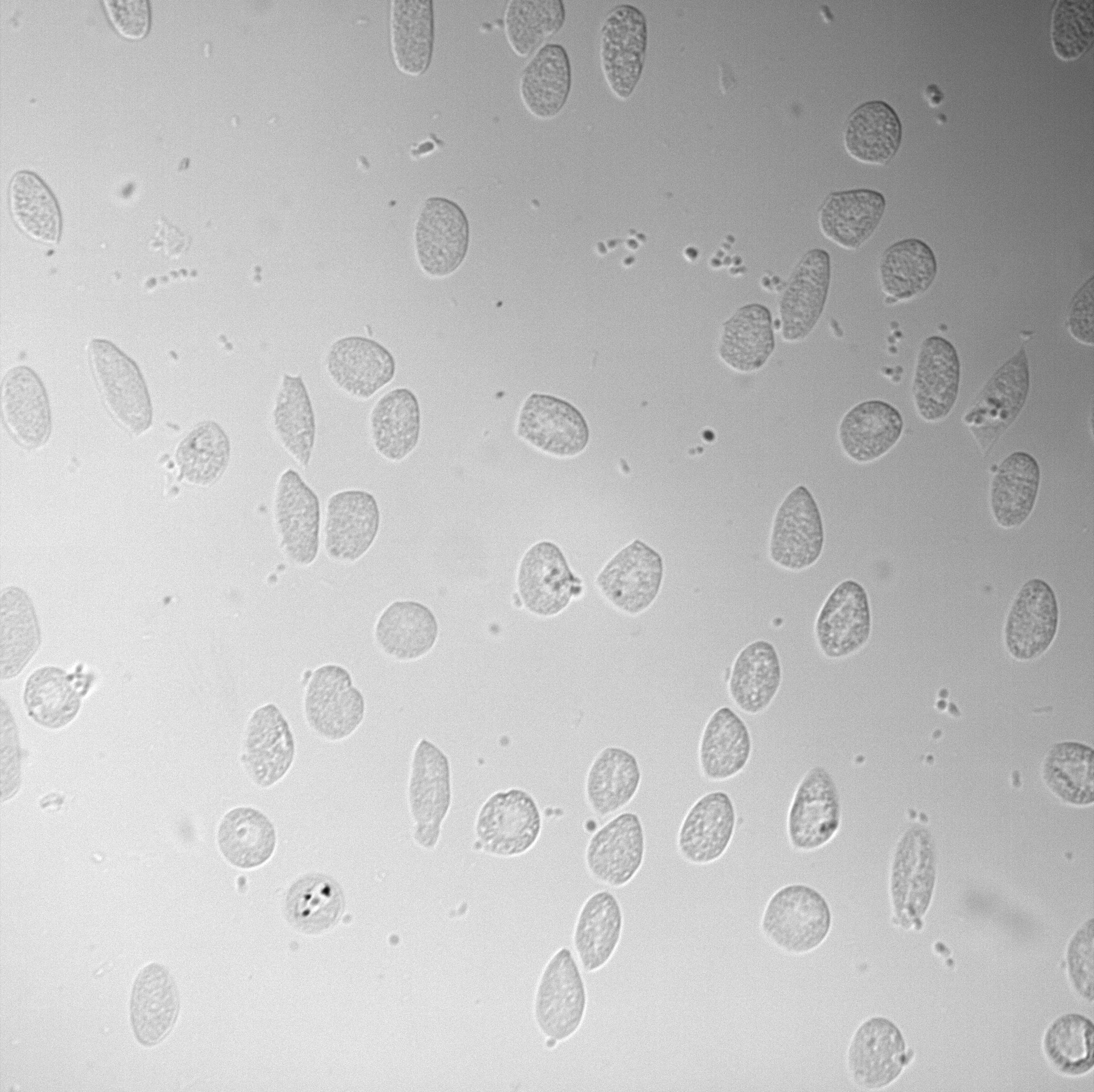

Supplement: Figure 3—figure supplement 6—source data 1. — Raw microscopy images of transgenic PF3D7_0702500-3xHA parasites probed with α-HA and α-SBP1. [file elife-107860-fig3-figsupp6-data1.zip › Figure 3 - Supplement 6 - Source Data 1 Raw Images/HA_SBP1007-0004.jpg]

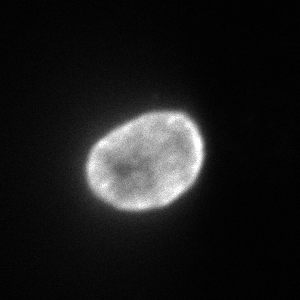

Supplement: Figure 3—figure supplement 6—source data 2. — Raw microscopy images of transgenic PF3D7_0702500-3xHA parasites probed with α-HA and α-KAHRP. [file elife-107860-fig3-figsupp6-data2.zip › Figure 3 - Supplement 6 - Source Data 2 Raw Images/Cropped/HA_KAHRP001.nd2 - T=0-1.tif]

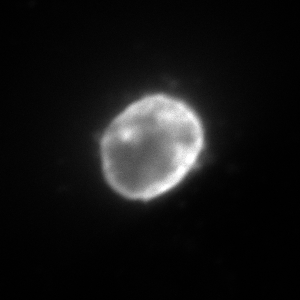

Supplement: Figure 3—figure supplement 6—source data 2. — Raw microscopy images of transgenic PF3D7_0702500-3xHA parasites probed with α-HA and α-KAHRP. [file elife-107860-fig3-figsupp6-data2.zip › Figure 3 - Supplement 6 - Source Data 2 Raw Images/Cropped/HA_KAHRP002.nd2 - T=0-1.tif]

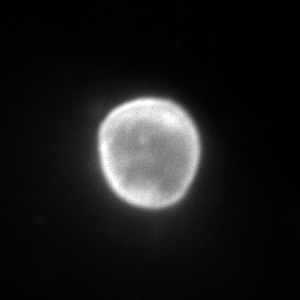

Supplement: Figure 3—figure supplement 6—source data 2. — Raw microscopy images of transgenic PF3D7_0702500-3xHA parasites probed with α-HA and α-KAHRP. [file elife-107860-fig3-figsupp6-data2.zip › Figure 3 - Supplement 6 - Source Data 2 Raw Images/Cropped/HA_KAHRP003.nd2 - T=0-1.tif]

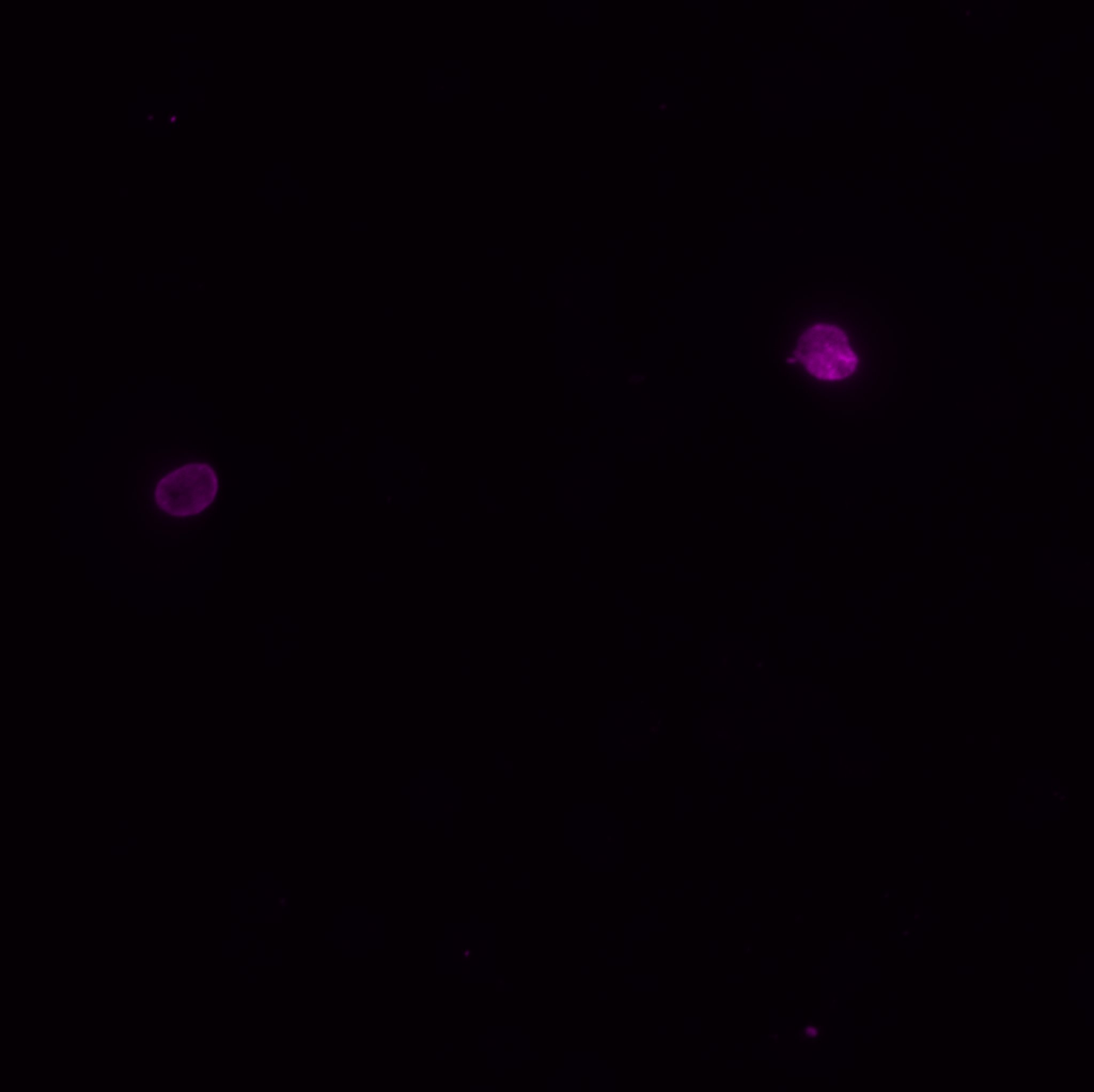

Supplement: Figure 3—figure supplement 6—source data 2. — Raw microscopy images of transgenic PF3D7_0702500-3xHA parasites probed with α-HA and α-KAHRP. [file elife-107860-fig3-figsupp6-data2.zip › Figure 3 - Supplement 6 - Source Data 2 Raw Images/HA_KAHRP001-0001.jpg]

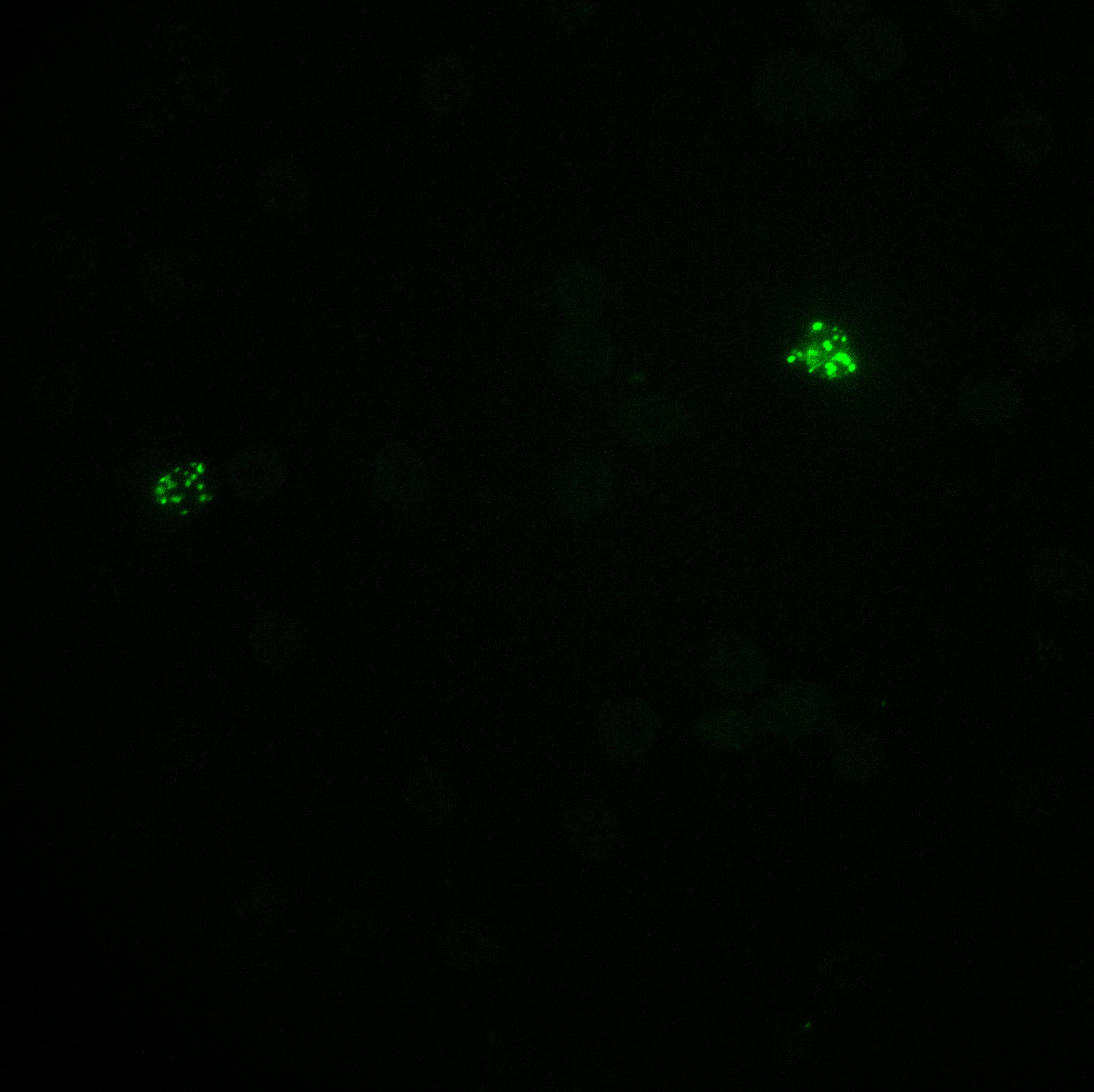

Supplement: Figure 3—figure supplement 6—source data 2. — Raw microscopy images of transgenic PF3D7_0702500-3xHA parasites probed with α-HA and α-KAHRP. [file elife-107860-fig3-figsupp6-data2.zip › Figure 3 - Supplement 6 - Source Data 2 Raw Images/HA_KAHRP001-0002.jpg]

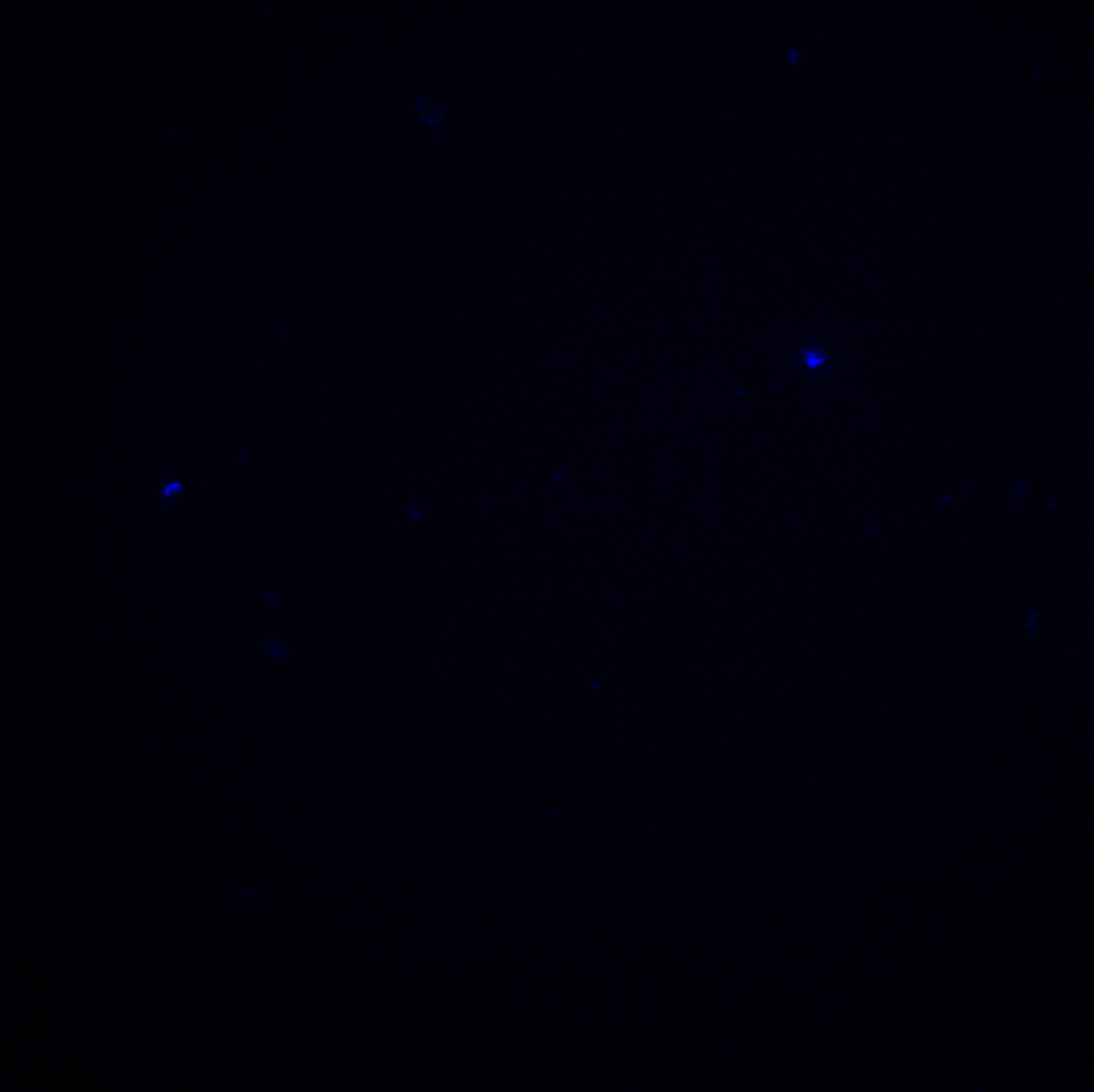

Supplement: Figure 3—figure supplement 6—source data 2. — Raw microscopy images of transgenic PF3D7_0702500-3xHA parasites probed with α-HA and α-KAHRP. [file elife-107860-fig3-figsupp6-data2.zip › Figure 3 - Supplement 6 - Source Data 2 Raw Images/HA_KAHRP001-0003.jpg]

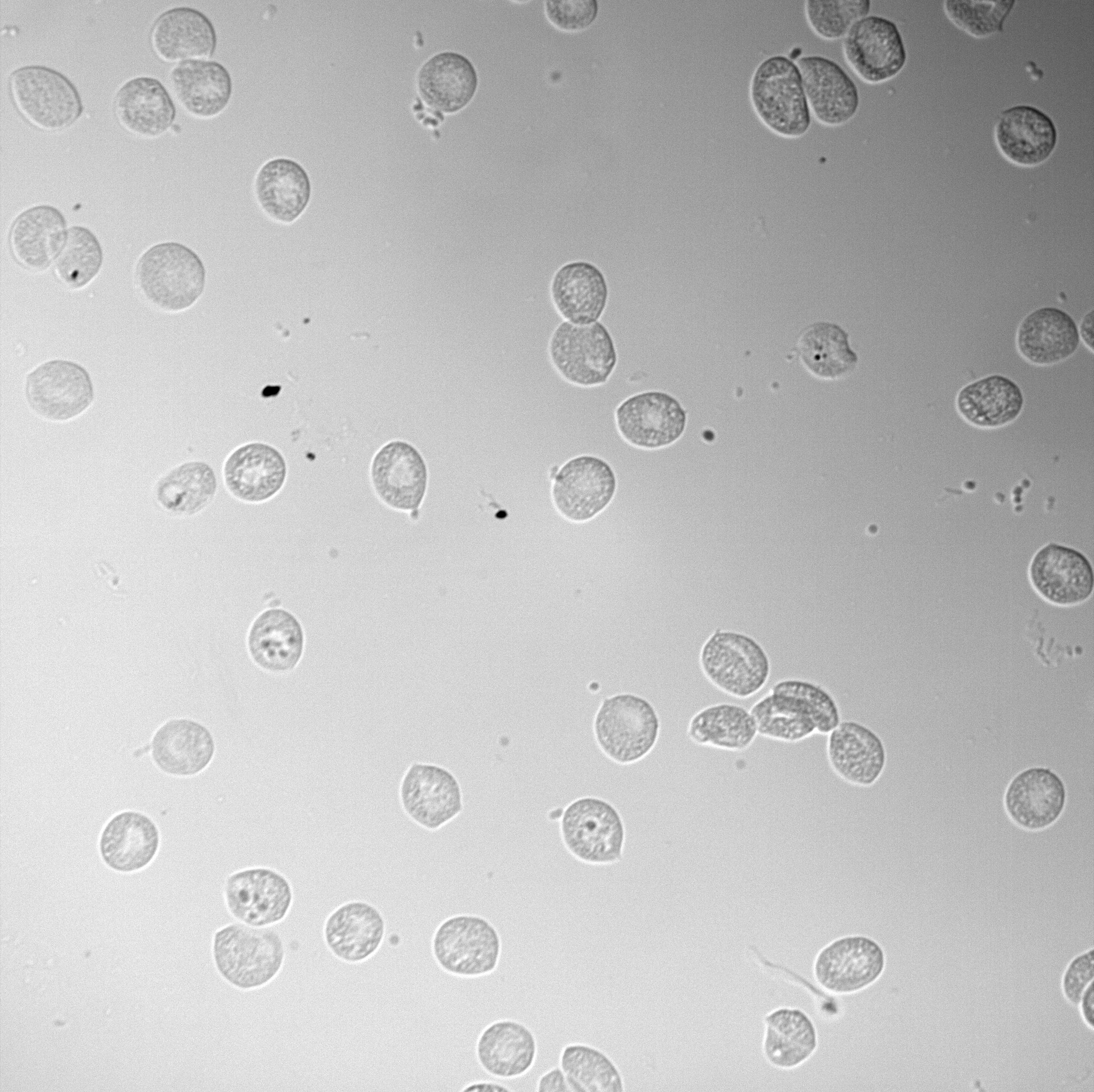

Supplement: Figure 3—figure supplement 6—source data 2. — Raw microscopy images of transgenic PF3D7_0702500-3xHA parasites probed with α-HA and α-KAHRP. [file elife-107860-fig3-figsupp6-data2.zip › Figure 3 - Supplement 6 - Source Data 2 Raw Images/HA_KAHRP001-0004.jpg]

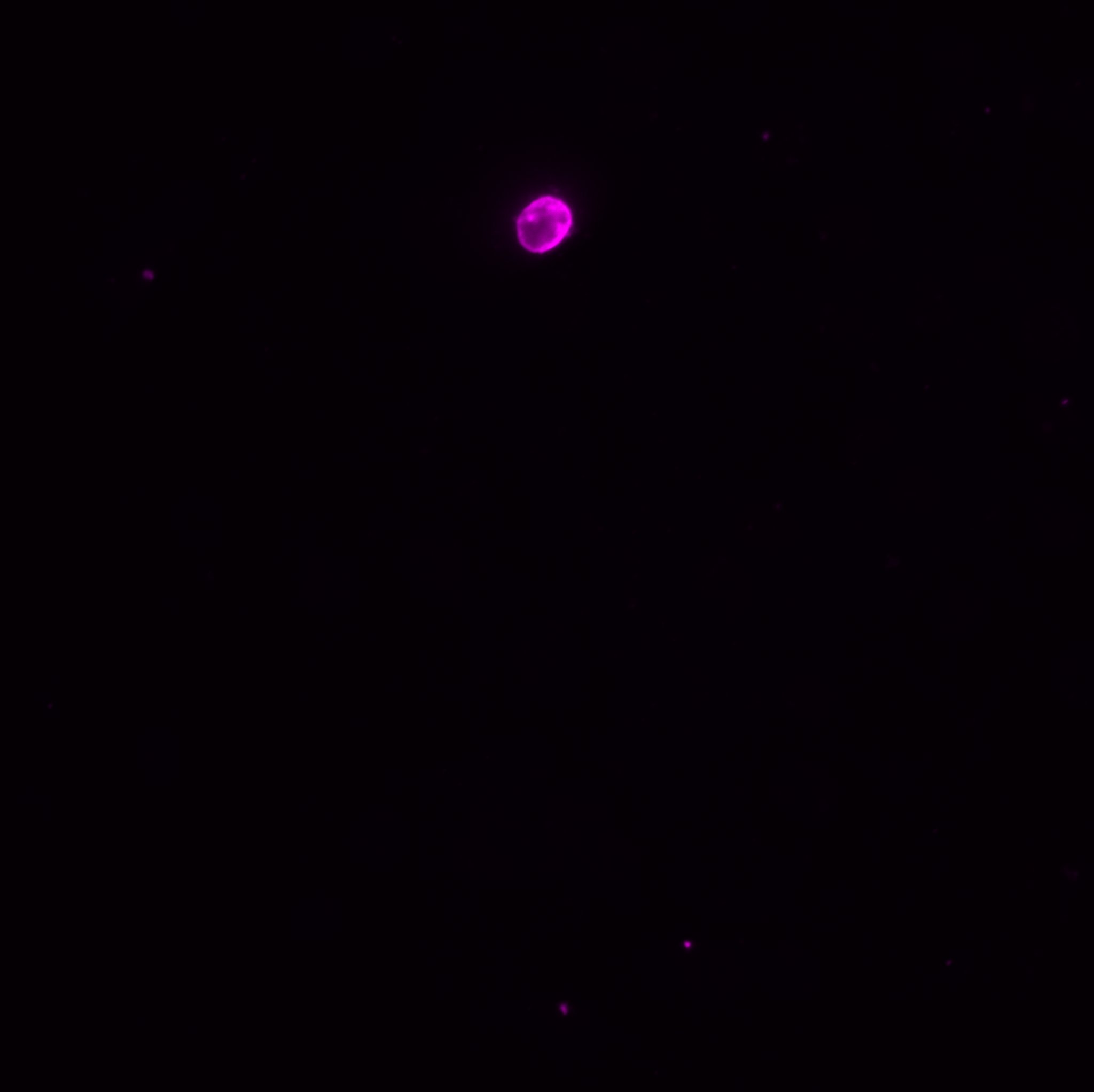

Supplement: Figure 3—figure supplement 6—source data 2. — Raw microscopy images of transgenic PF3D7_0702500-3xHA parasites probed with α-HA and α-KAHRP. [file elife-107860-fig3-figsupp6-data2.zip › Figure 3 - Supplement 6 - Source Data 2 Raw Images/HA_KAHRP002-0001.jpg]

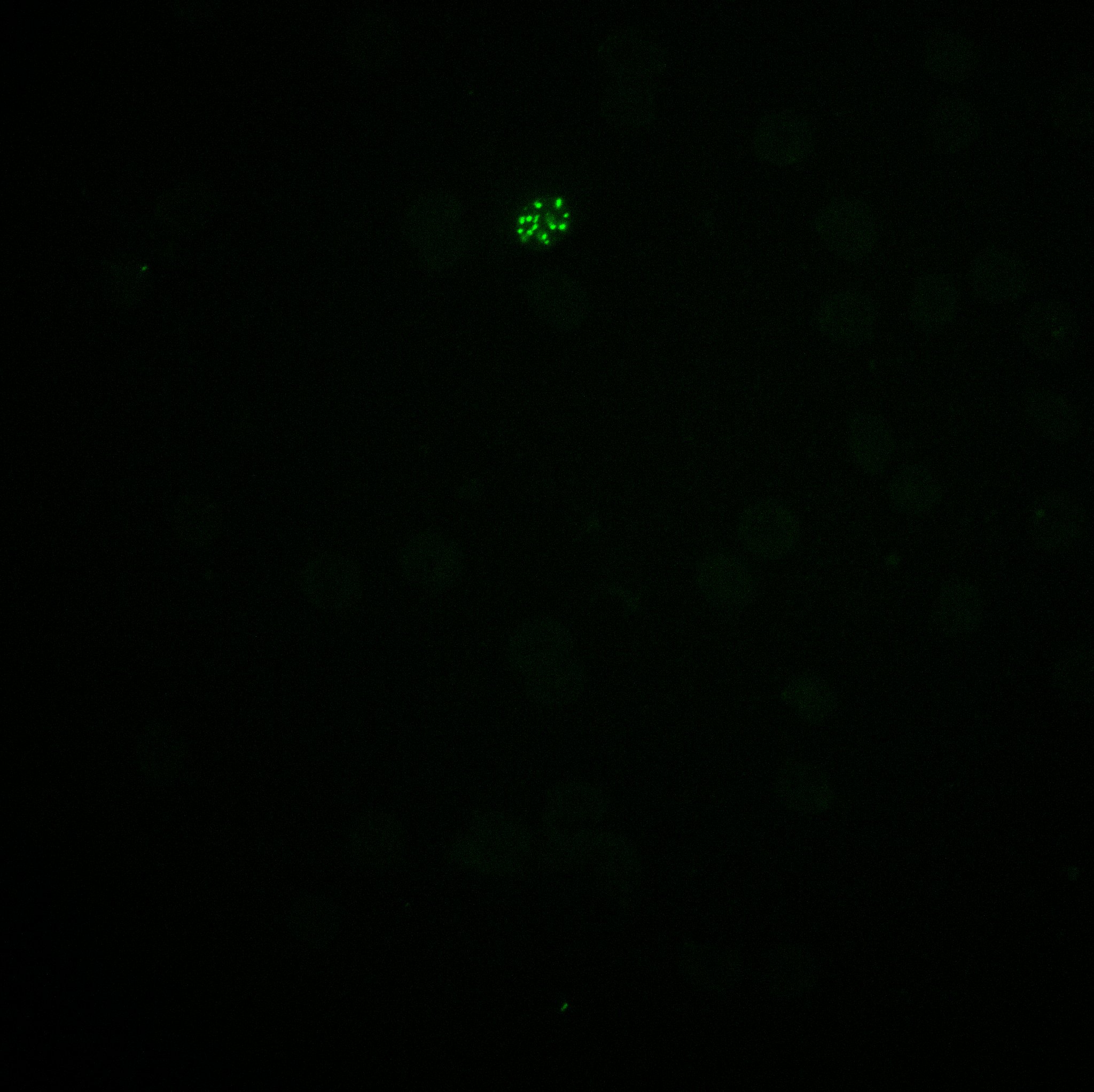

Supplement: Figure 3—figure supplement 6—source data 2. — Raw microscopy images of transgenic PF3D7_0702500-3xHA parasites probed with α-HA and α-KAHRP. [file elife-107860-fig3-figsupp6-data2.zip › Figure 3 - Supplement 6 - Source Data 2 Raw Images/HA_KAHRP002-0002.jpg]

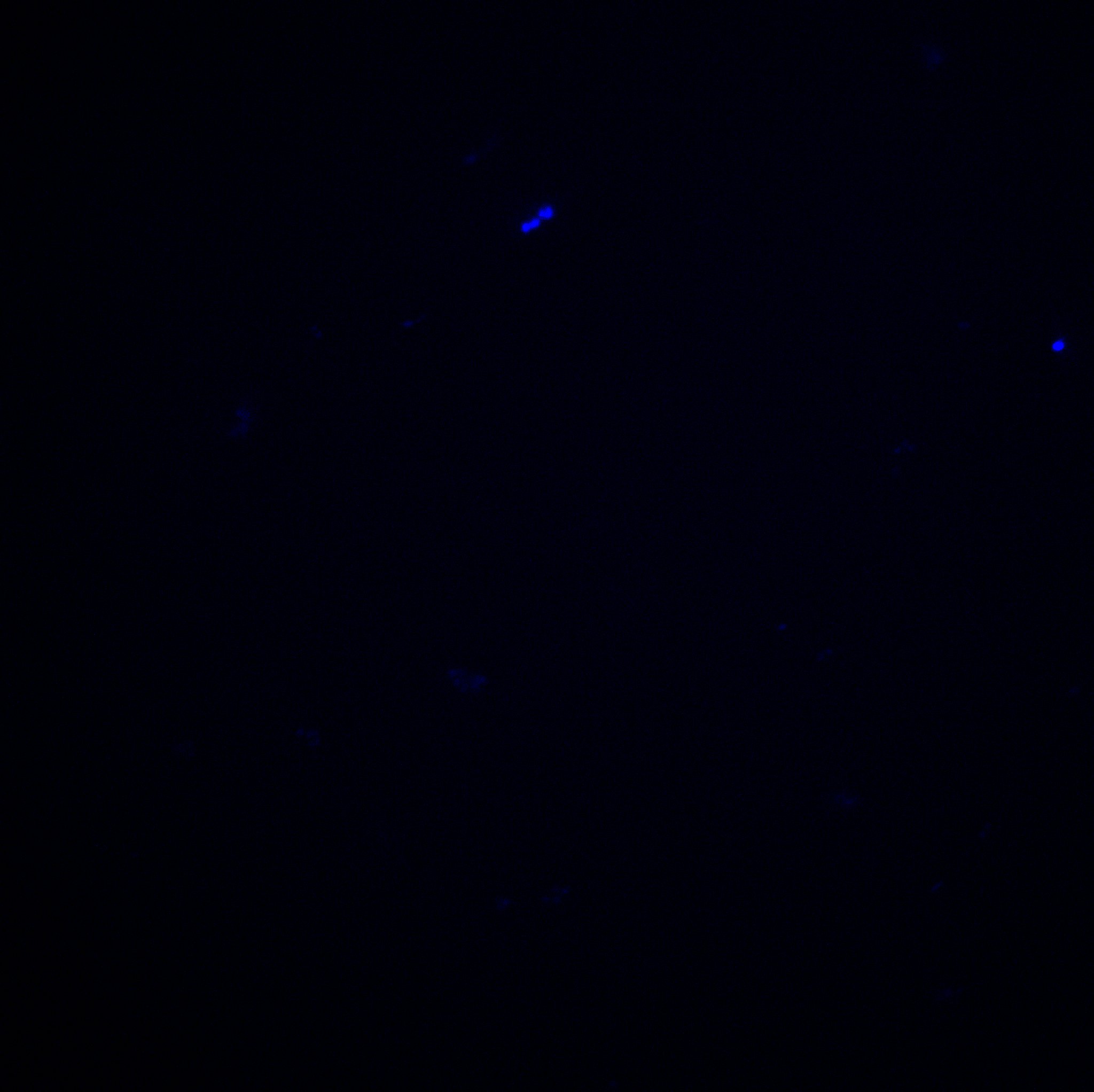

Supplement: Figure 3—figure supplement 6—source data 2. — Raw microscopy images of transgenic PF3D7_0702500-3xHA parasites probed with α-HA and α-KAHRP. [file elife-107860-fig3-figsupp6-data2.zip › Figure 3 - Supplement 6 - Source Data 2 Raw Images/HA_KAHRP002-0003.jpg]

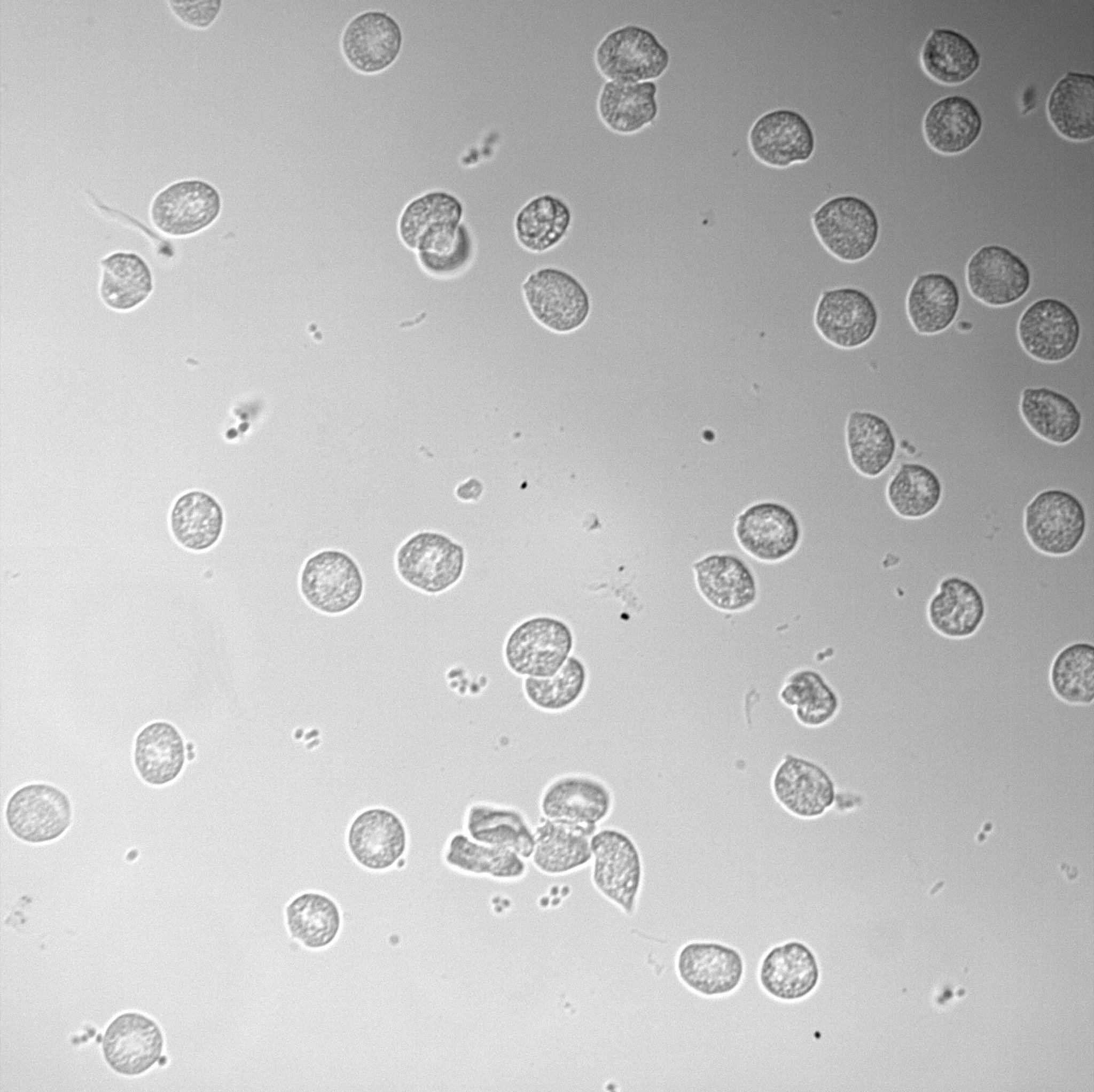

Supplement: Figure 3—figure supplement 6—source data 2. — Raw microscopy images of transgenic PF3D7_0702500-3xHA parasites probed with α-HA and α-KAHRP. [file elife-107860-fig3-figsupp6-data2.zip › Figure 3 - Supplement 6 - Source Data 2 Raw Images/HA_KAHRP002-0004.jpg]

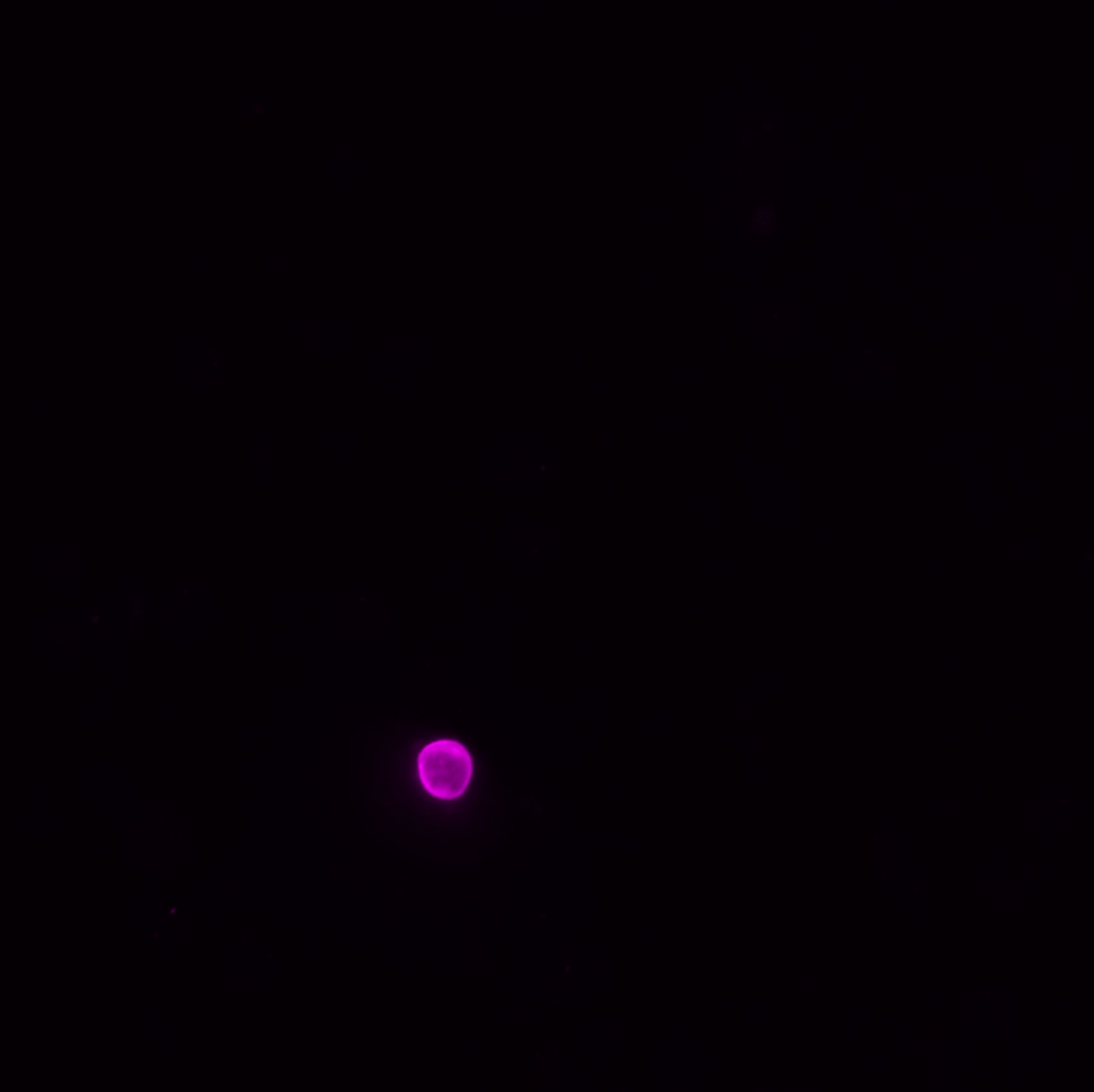

Supplement: Figure 3—figure supplement 6—source data 2. — Raw microscopy images of transgenic PF3D7_0702500-3xHA parasites probed with α-HA and α-KAHRP. [file elife-107860-fig3-figsupp6-data2.zip › Figure 3 - Supplement 6 - Source Data 2 Raw Images/HA_KAHRP003-0001.jpg]

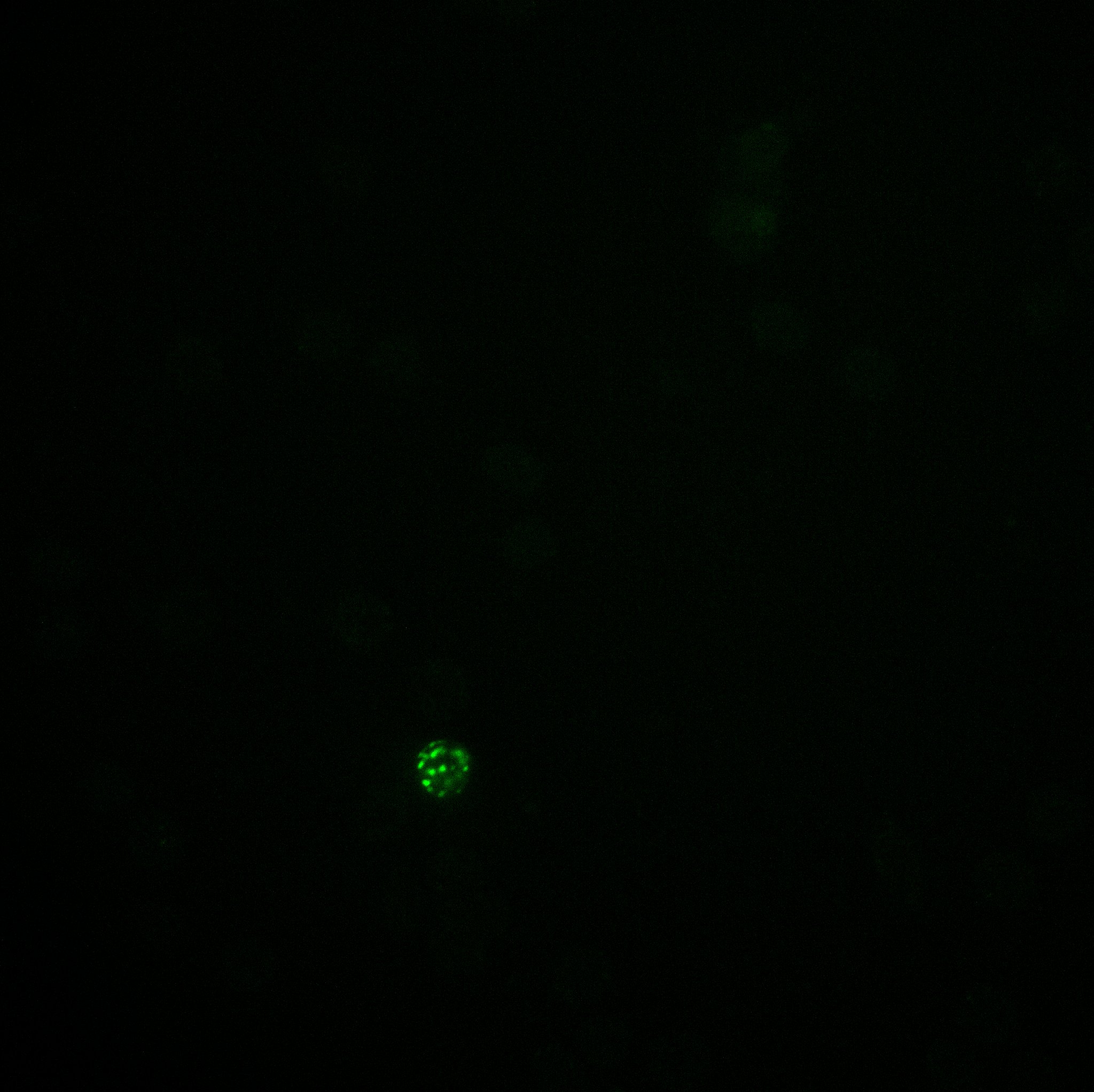

Supplement: Figure 3—figure supplement 6—source data 2. — Raw microscopy images of transgenic PF3D7_0702500-3xHA parasites probed with α-HA and α-KAHRP. [file elife-107860-fig3-figsupp6-data2.zip › Figure 3 - Supplement 6 - Source Data 2 Raw Images/HA_KAHRP003-0002.jpg]

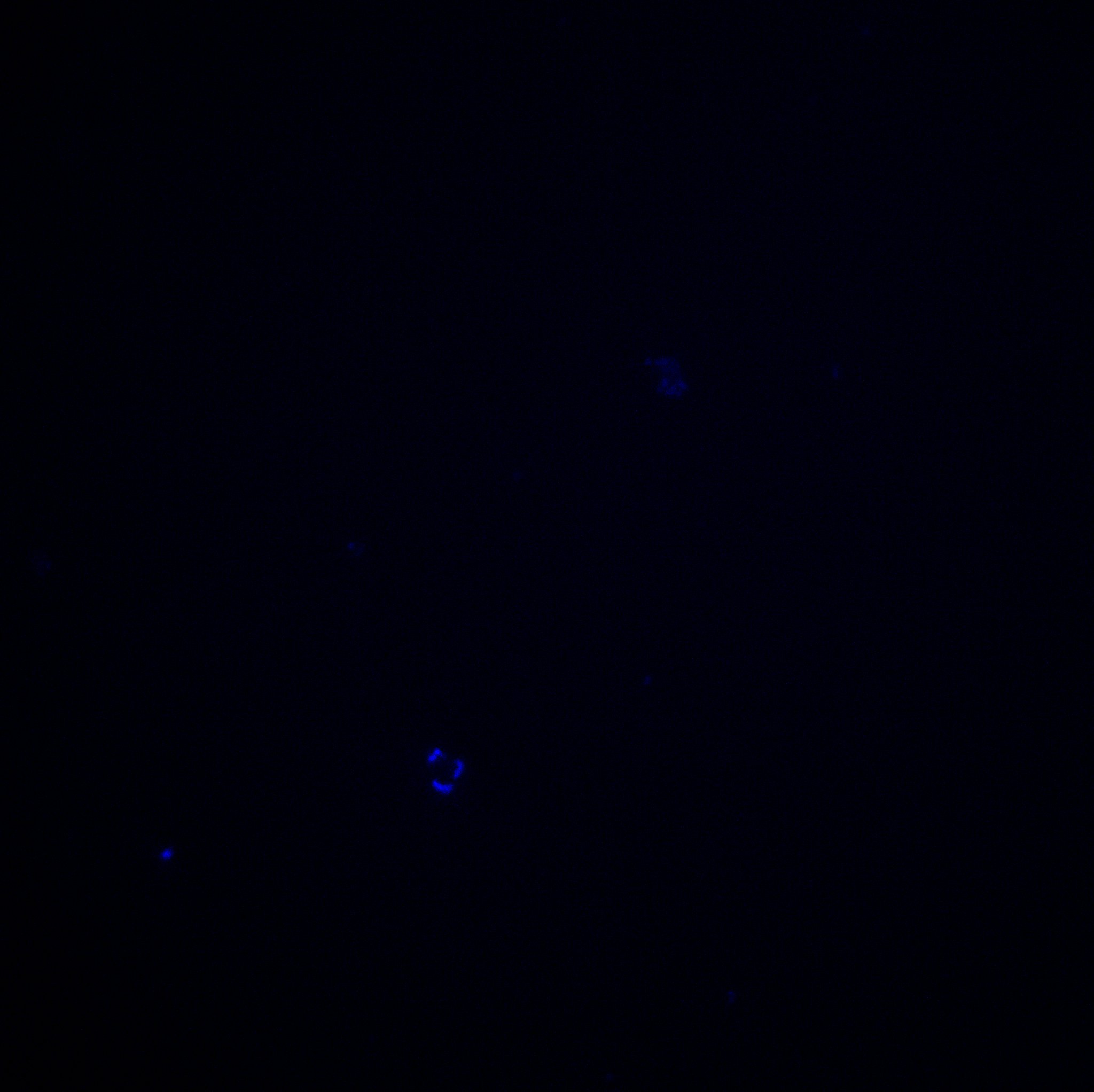

Supplement: Figure 3—figure supplement 6—source data 2. — Raw microscopy images of transgenic PF3D7_0702500-3xHA parasites probed with α-HA and α-KAHRP. [file elife-107860-fig3-figsupp6-data2.zip › Figure 3 - Supplement 6 - Source Data 2 Raw Images/HA_KAHRP003-0003.jpg]

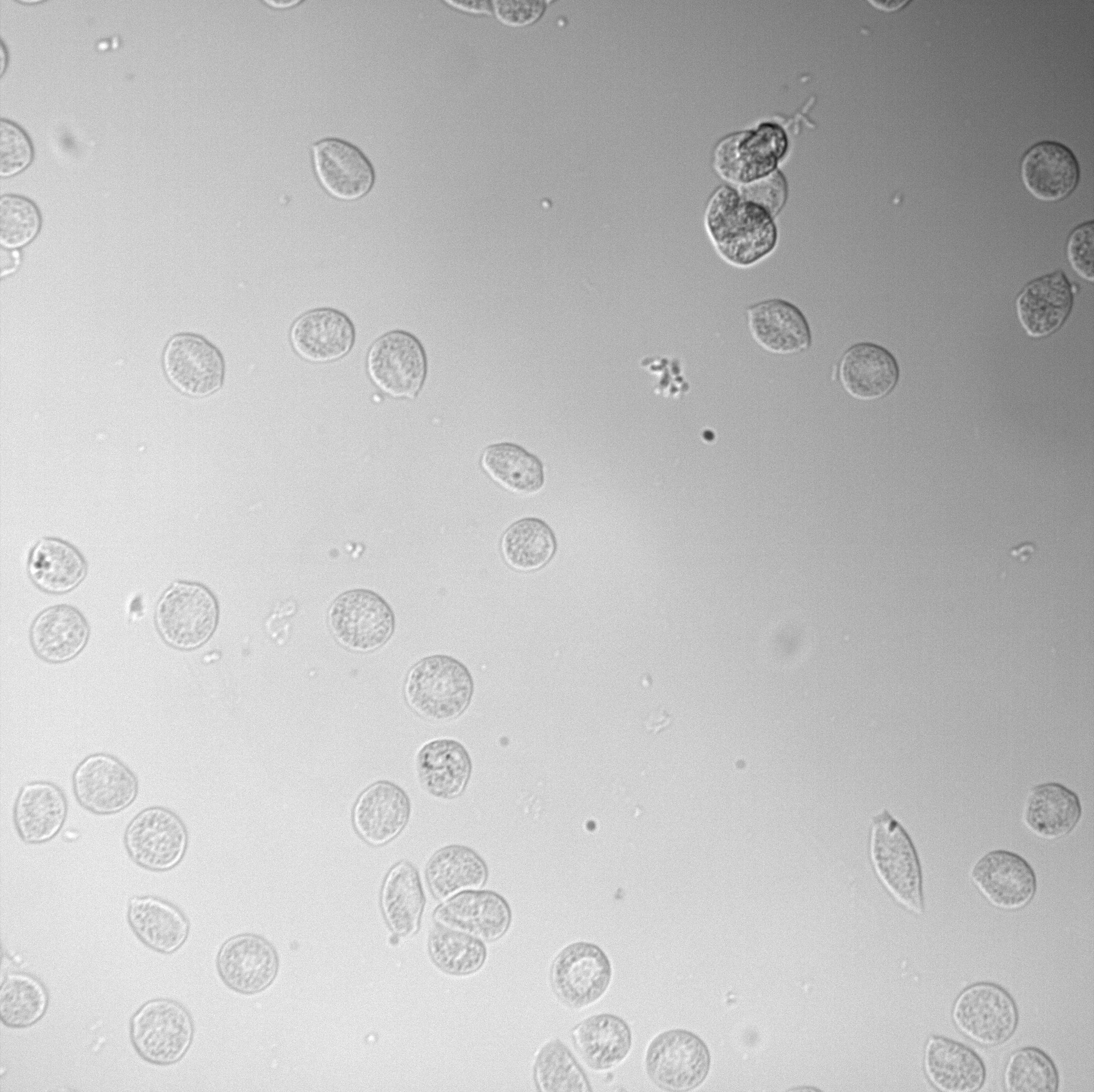

Supplement: Figure 3—figure supplement 6—source data 2. — Raw microscopy images of transgenic PF3D7_0702500-3xHA parasites probed with α-HA and α-KAHRP. [file elife-107860-fig3-figsupp6-data2.zip › Figure 3 - Supplement 6 - Source Data 2 Raw Images/HA_KAHRP003-0004.jpg]

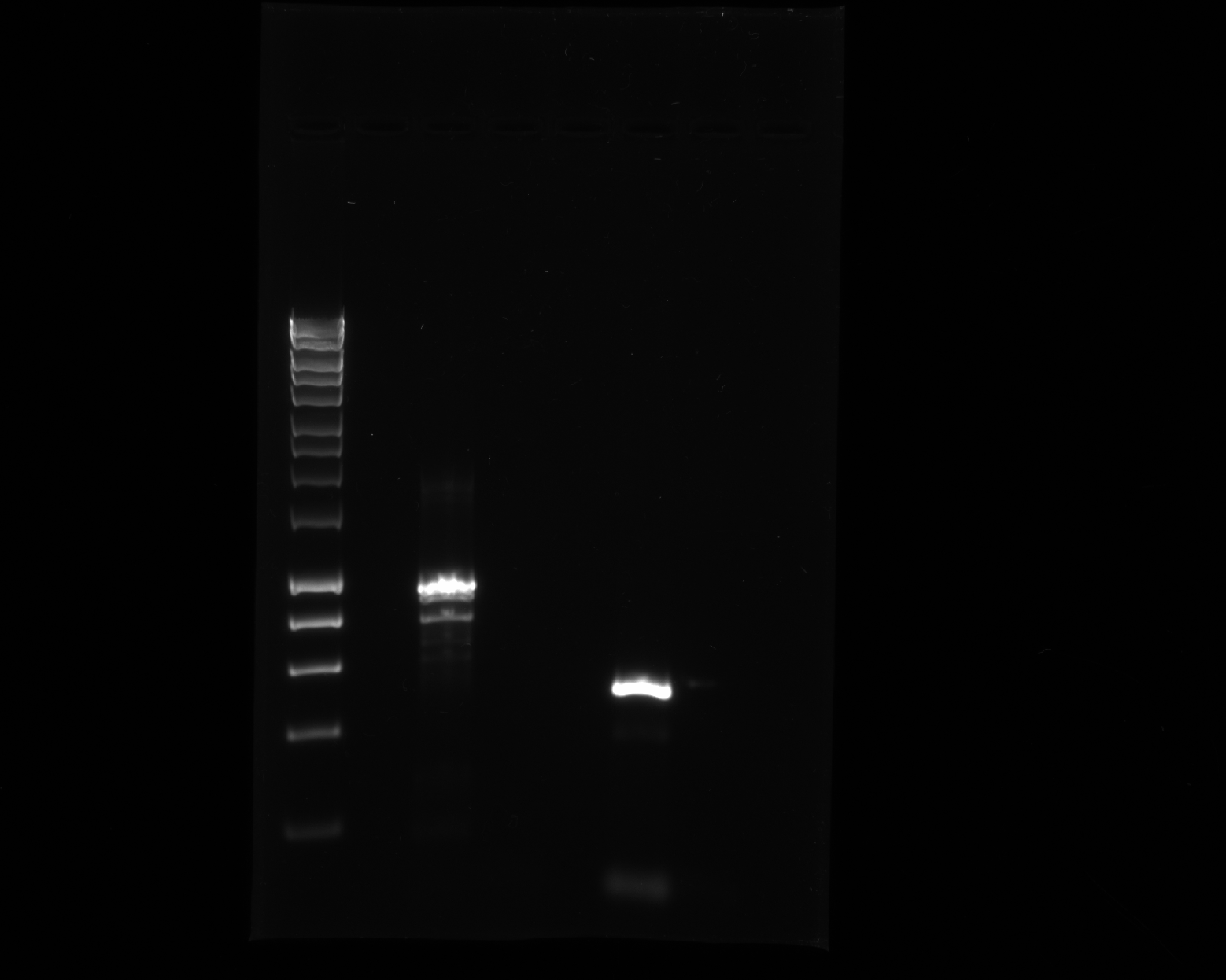

Supplement: Figure 4—figure supplement 1—source data 1. [file elife-107860-fig4-figsupp1-data1.zip › Figure 4 - Supplement 1 - Source Data 1 - Raw Image/Figure 4 - Supplement 1 - Source Data 1 - Raw Image.tif]

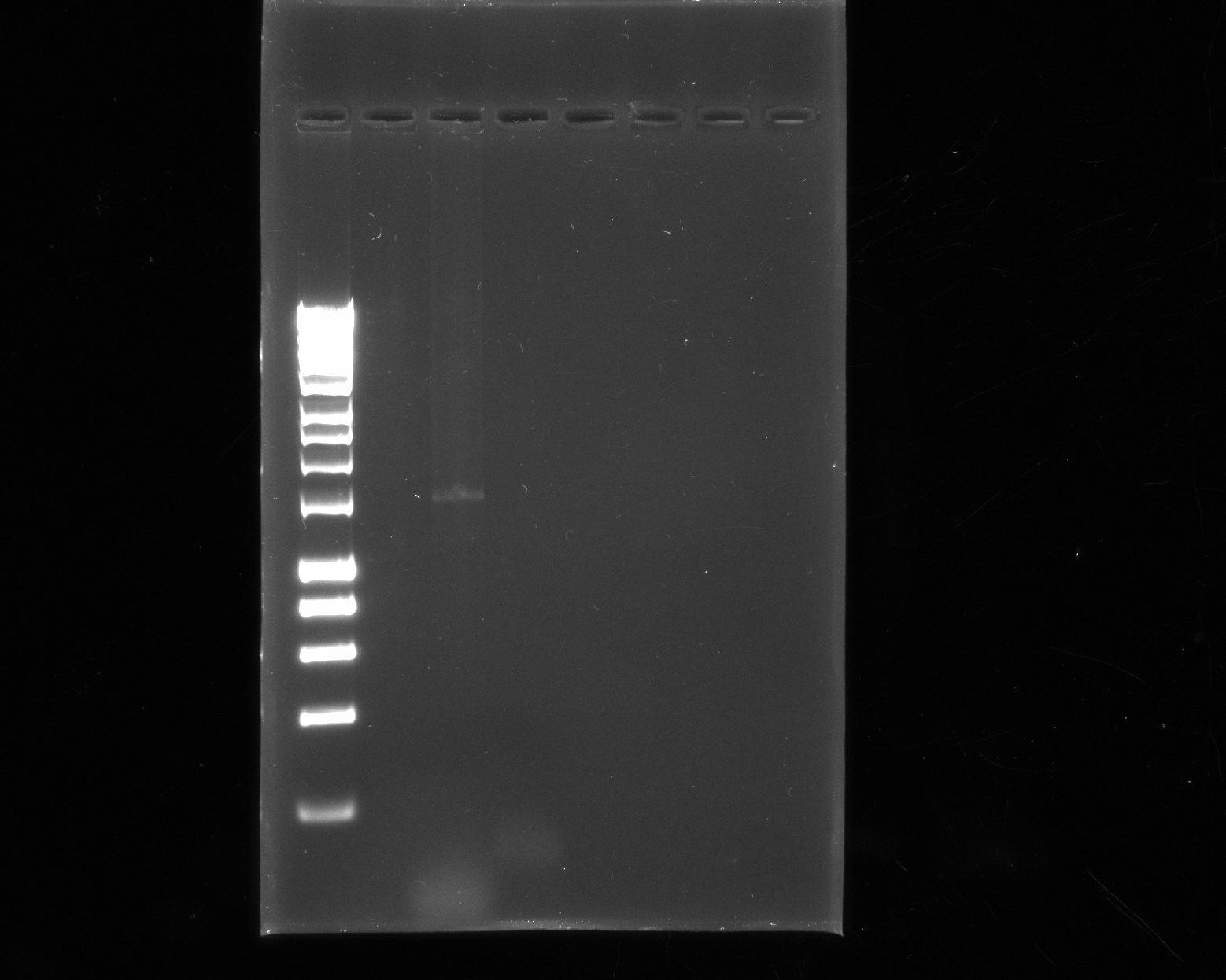

Supplement: Figure 4—figure supplement 1—source data 2. [file elife-107860-fig4-figsupp1-data2.zip › Figure 4 - Supplement 1 - Source Data 2 - Raw Images/Figure 4 - Supplement 1 - Source Data 2 - Raw Image.tif]

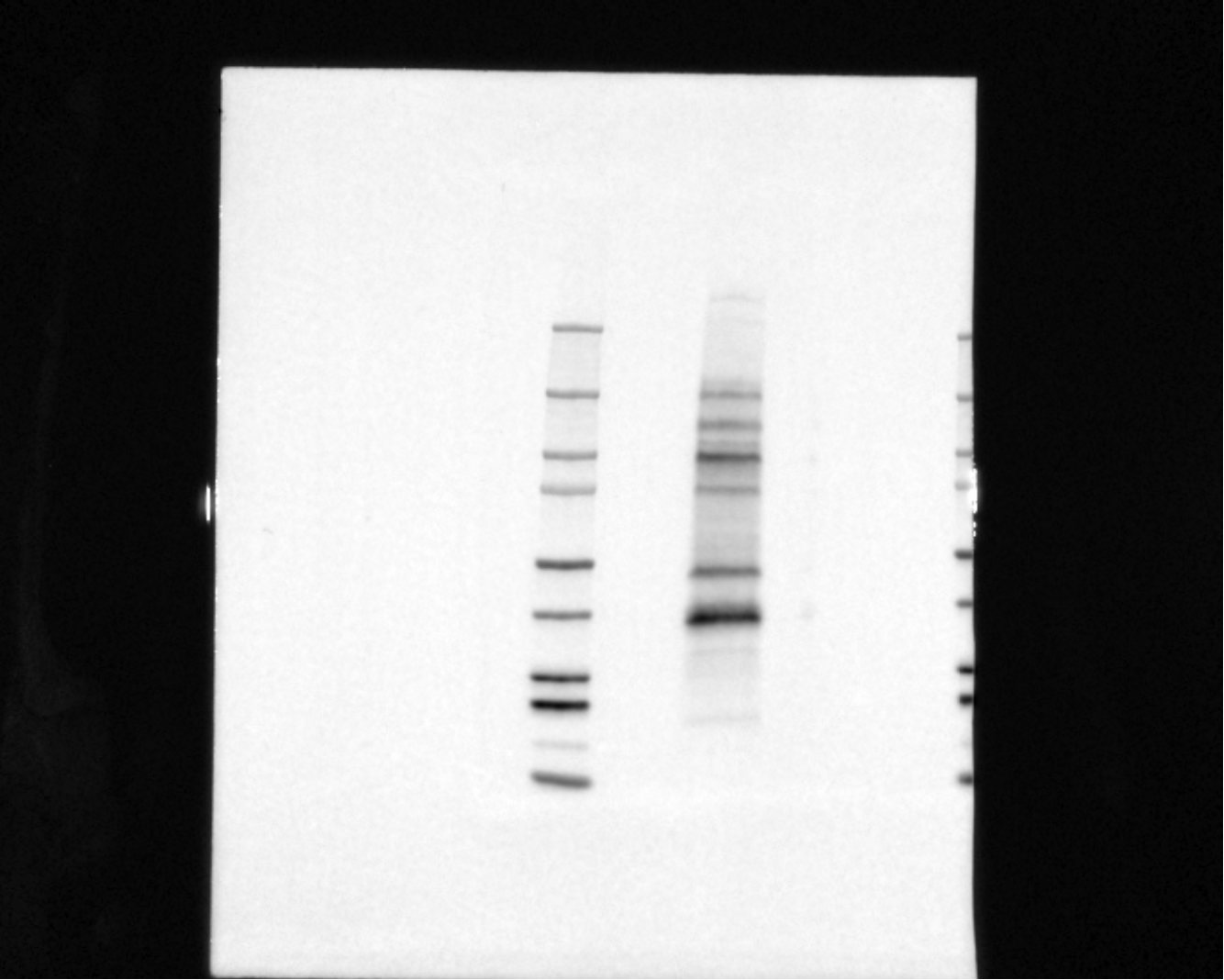

Supplement: Figure 4—figure supplement 1—source data 4. [file elife-107860-fig4-figsupp1-data4.zip › Figure 4 - Supplement 1 - Source Data 4 - Raw Image/Figure 4 - Supplement 1 - Source Data 4 - Raw Image.png]

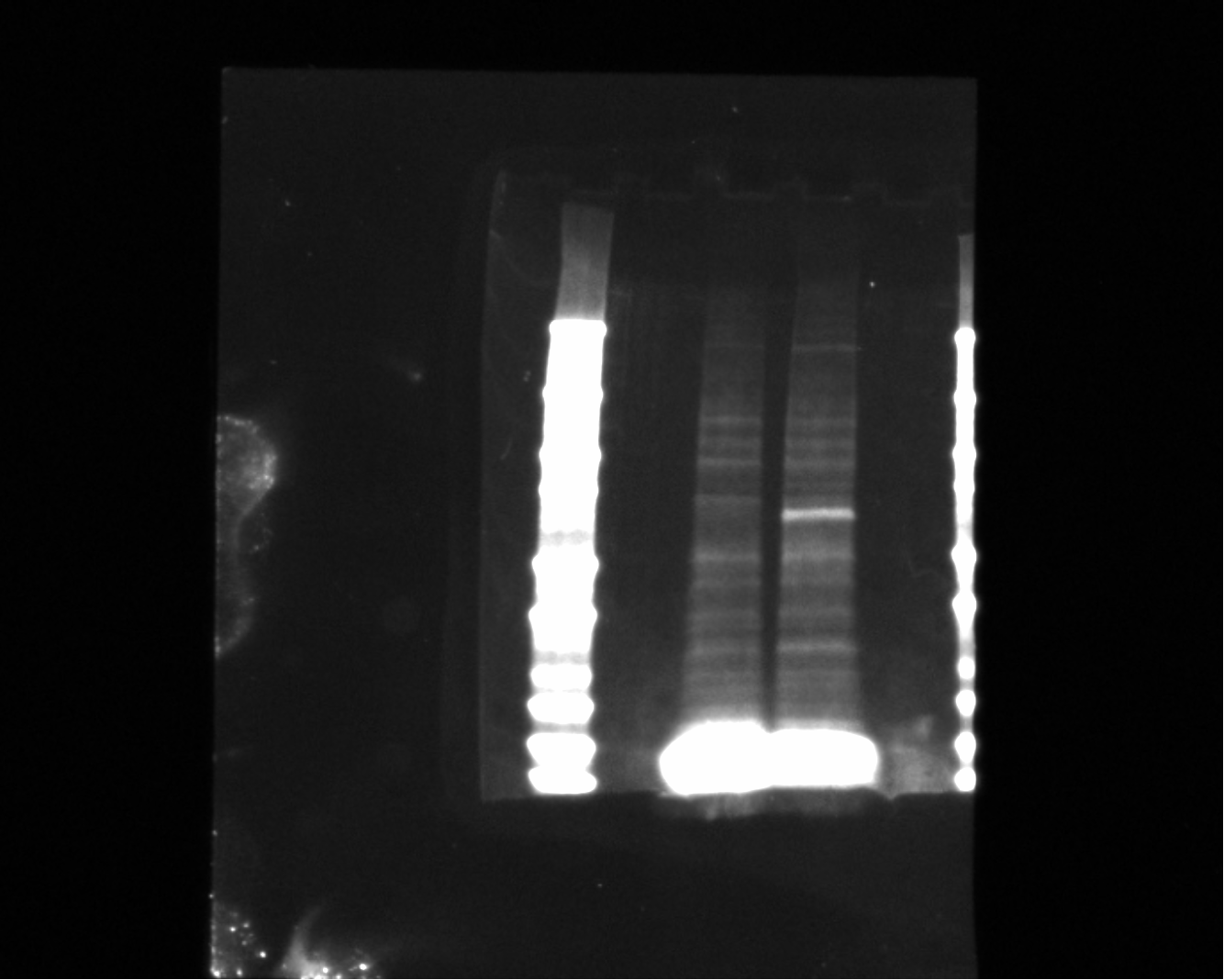

Supplement: Figure 4—figure supplement 1—source data 5. [file elife-107860-fig4-figsupp1-data5.zip › Figure 4 - Supplement 1 - Source Data 5 - Raw Images/Figure 4 - Supplement 1 - Source Data 5 - Raw Images - High Contrast.png]

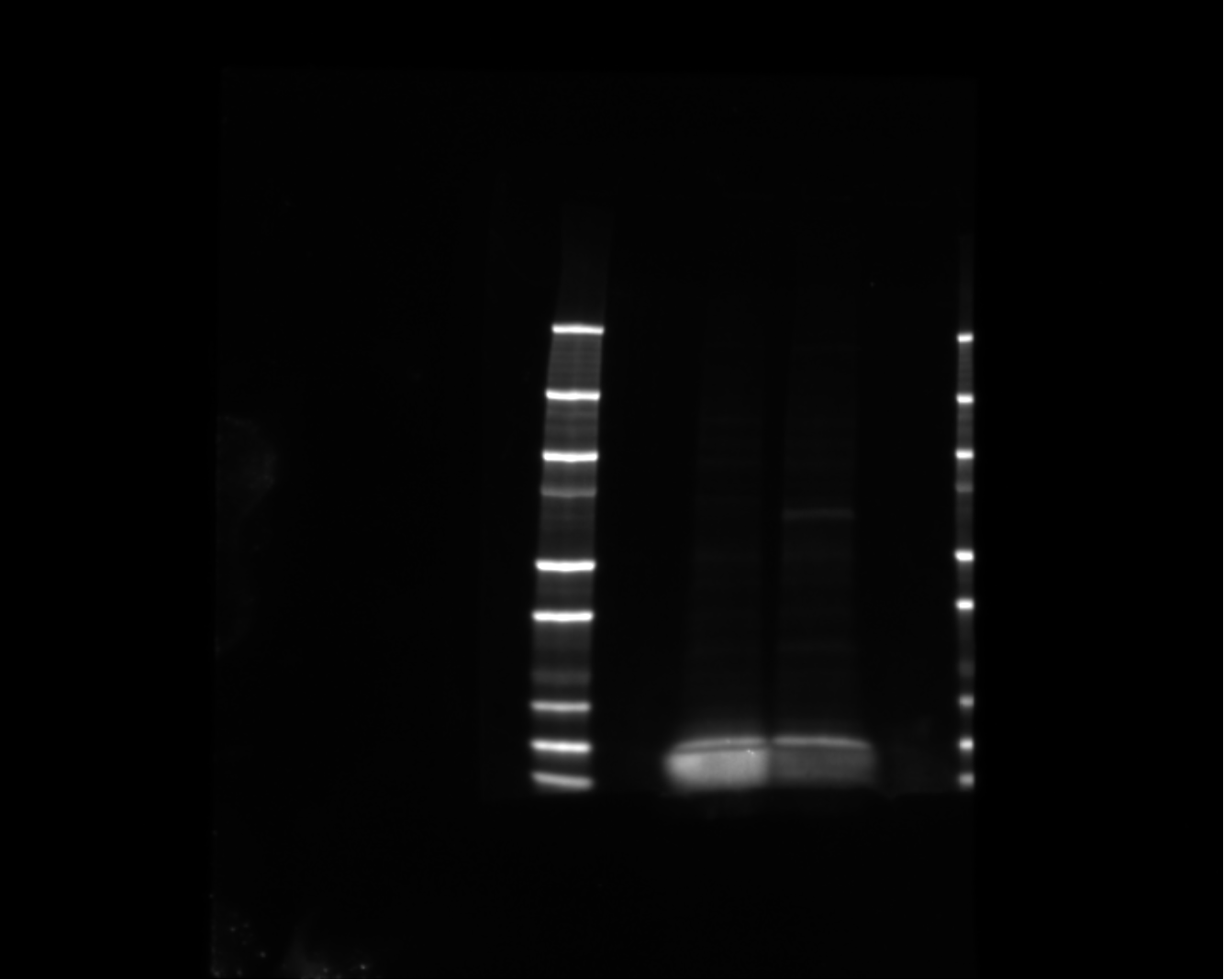

Supplement: Figure 4—figure supplement 1—source data 5. [file elife-107860-fig4-figsupp1-data5.zip › Figure 4 - Supplement 1 - Source Data 5 - Raw Images/Figure 4 - Supplement 1 - Source Data 5 - Raw Images - Low Contrast.png]

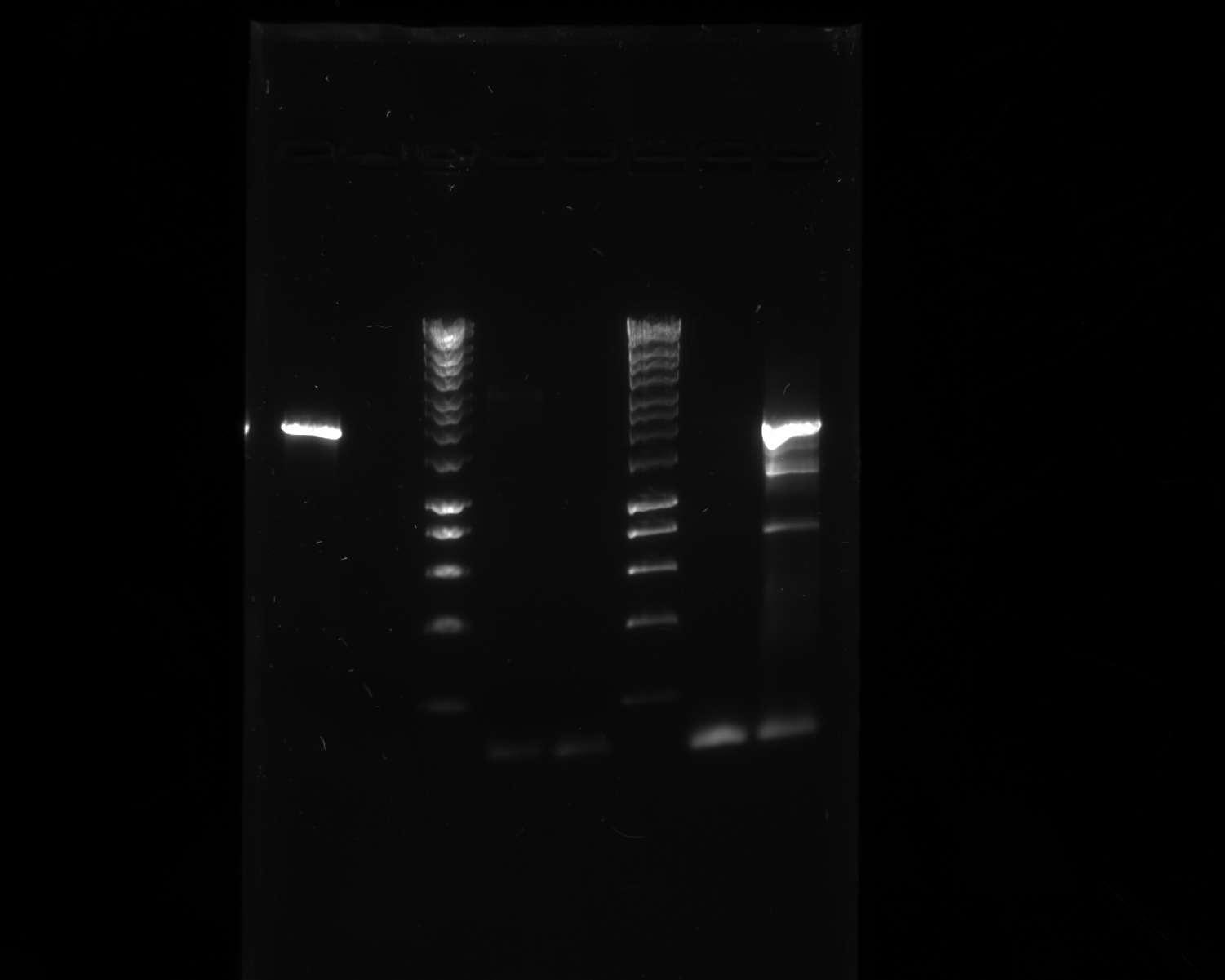

Supplement: Figure 5—figure supplement 1—source data 1. [file elife-107860-fig5-figsupp1-data1.zip › Figure 5 - Supplement 1 - Source Data 1 - Raw Images/Figure 5 - Supplement 1 - Source Data 1 - .Scn File.png]

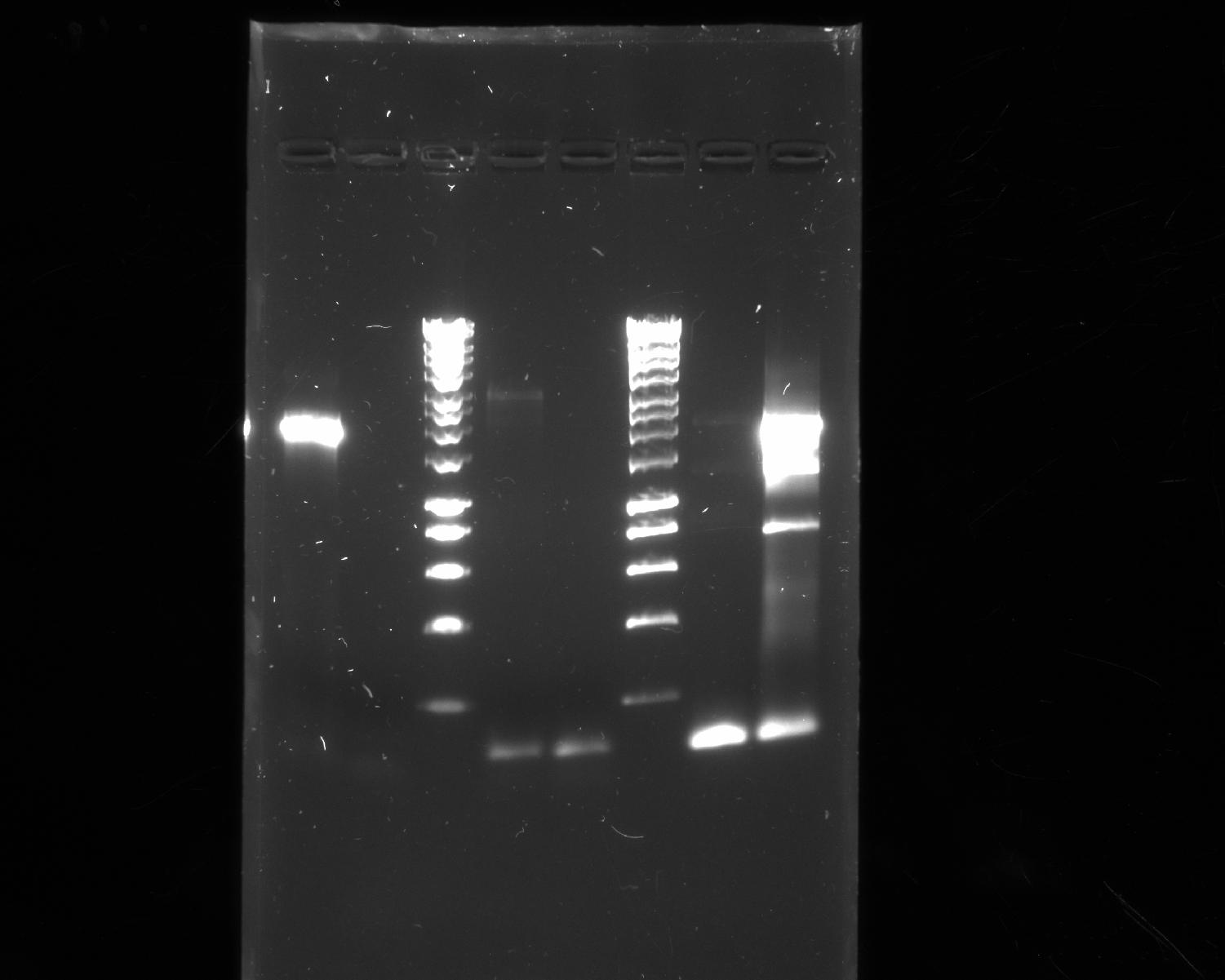

Supplement: Figure 5—figure supplement 1—source data 2. [file elife-107860-fig5-figsupp1-data2.zip › Figure 5 - Supplement 1 - Source Data 2 - Raw Images/Figure 5 - Supplement 1 - Source Data 2 - Raw Image.png]

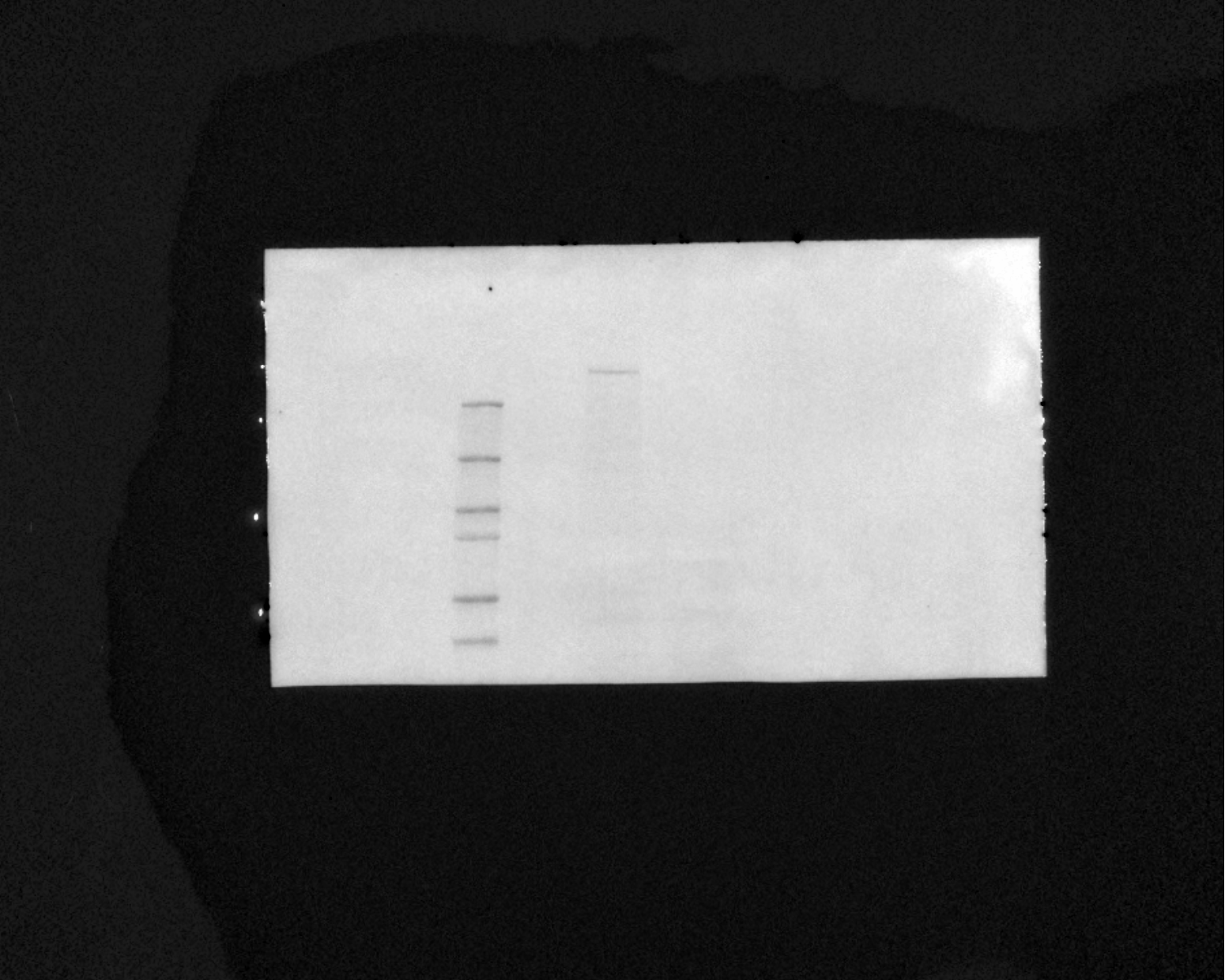

Supplement: Figure 5—figure supplement 1—source data 4. [file elife-107860-fig5-figsupp1-data4.zip › Figure 5 - Supplement 1 - Source Data 4 - Raw Images/Figure 5 - Supplement 1 - Source Data 4 - Raw Image.tif]

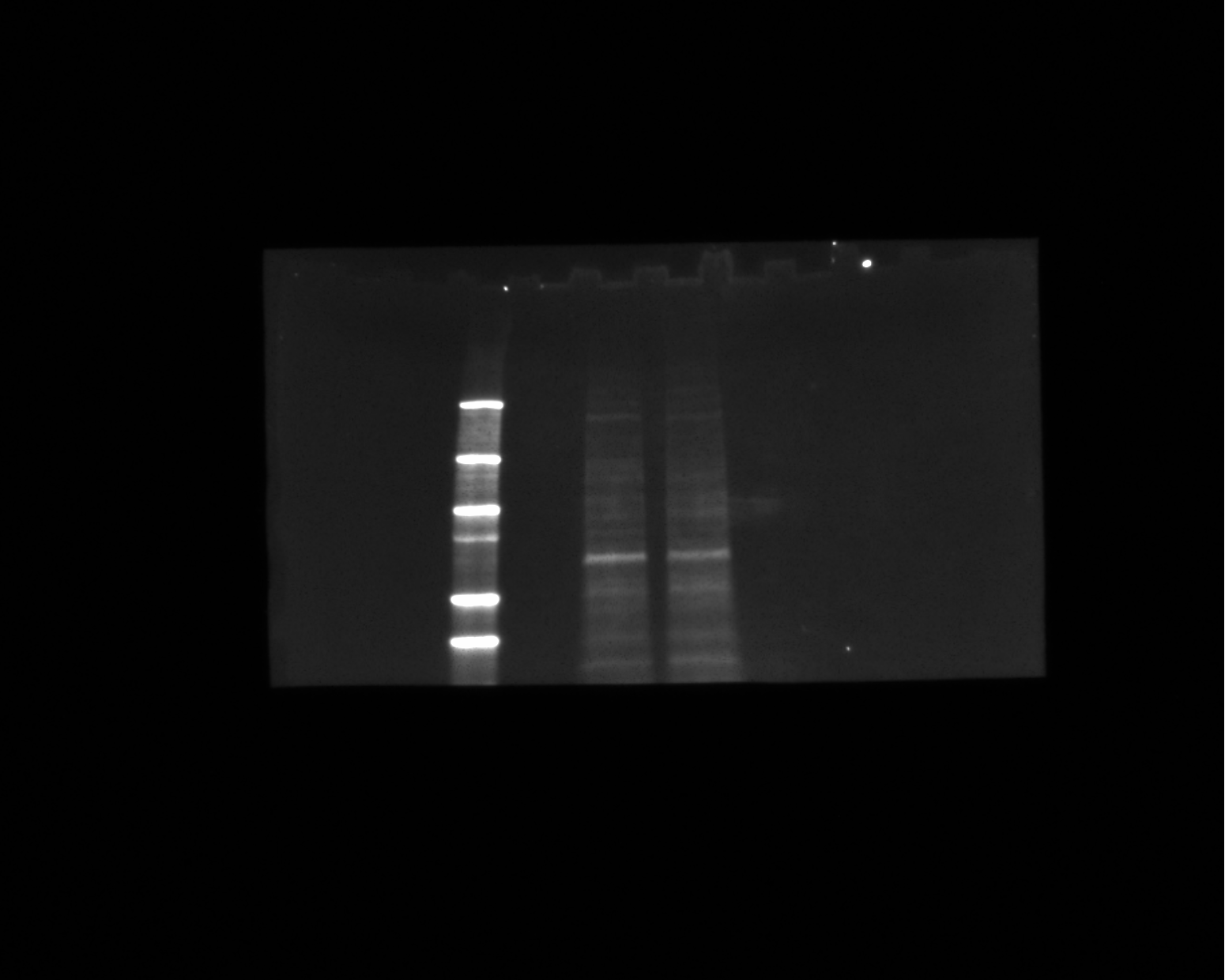

Supplement: Figure 5—figure supplement 1—source data 5. [file elife-107860-fig5-figsupp1-data5.zip › Figure 5 - Supplement 1 - Source Data 5 - Raw Images/Figure 5 - Supplement 1 - Source Data 5 - Raw Image.tif]

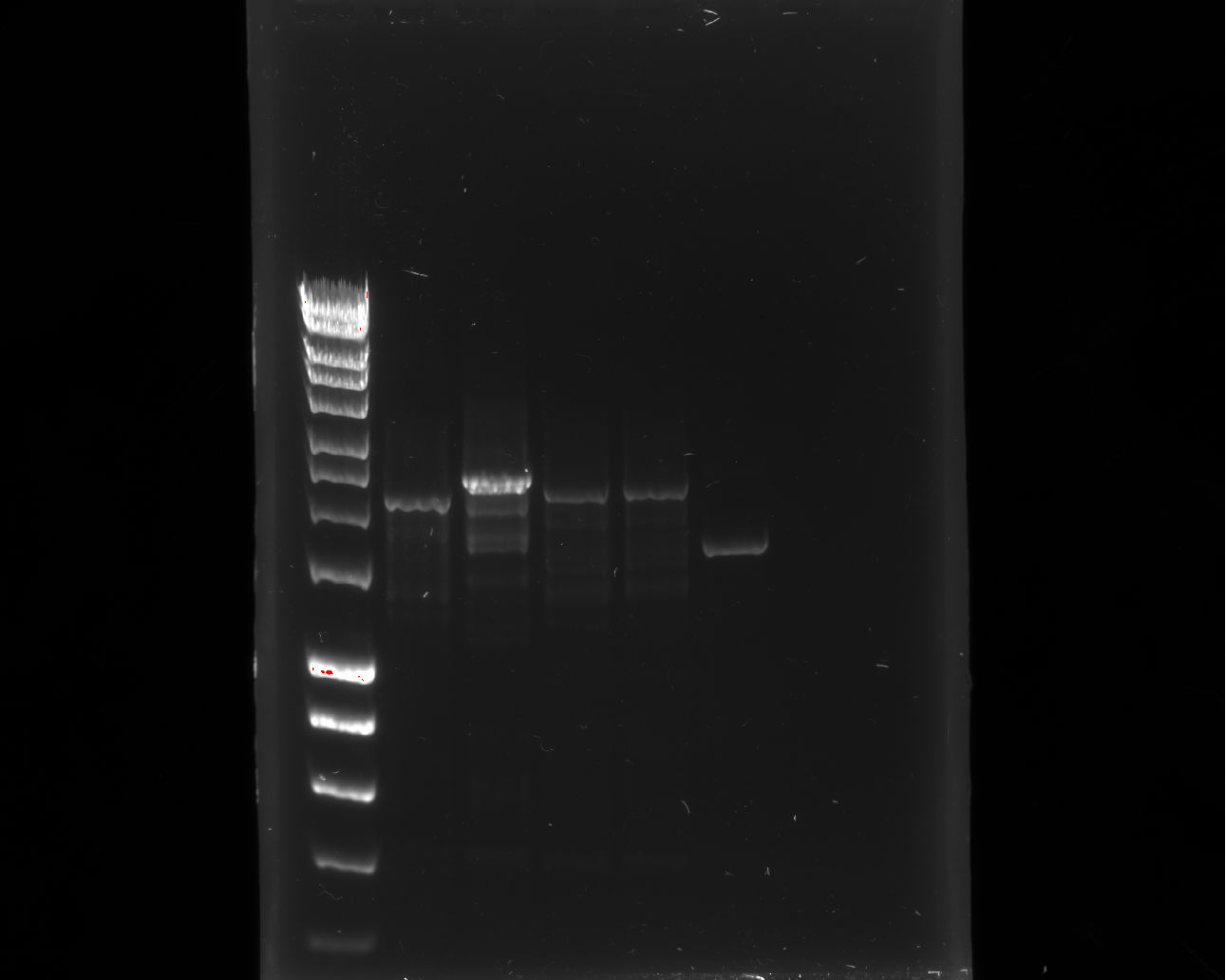

Supplement: Figure 6—figure supplement 1—source data 1. [file elife-107860-fig6-figsupp1-data1.zip › Figure 6 - Supplement 1 - Source Data 1 - Raw Images/Figure 6 - Supplement 1 - Source Data 1 - Raw Image.png]

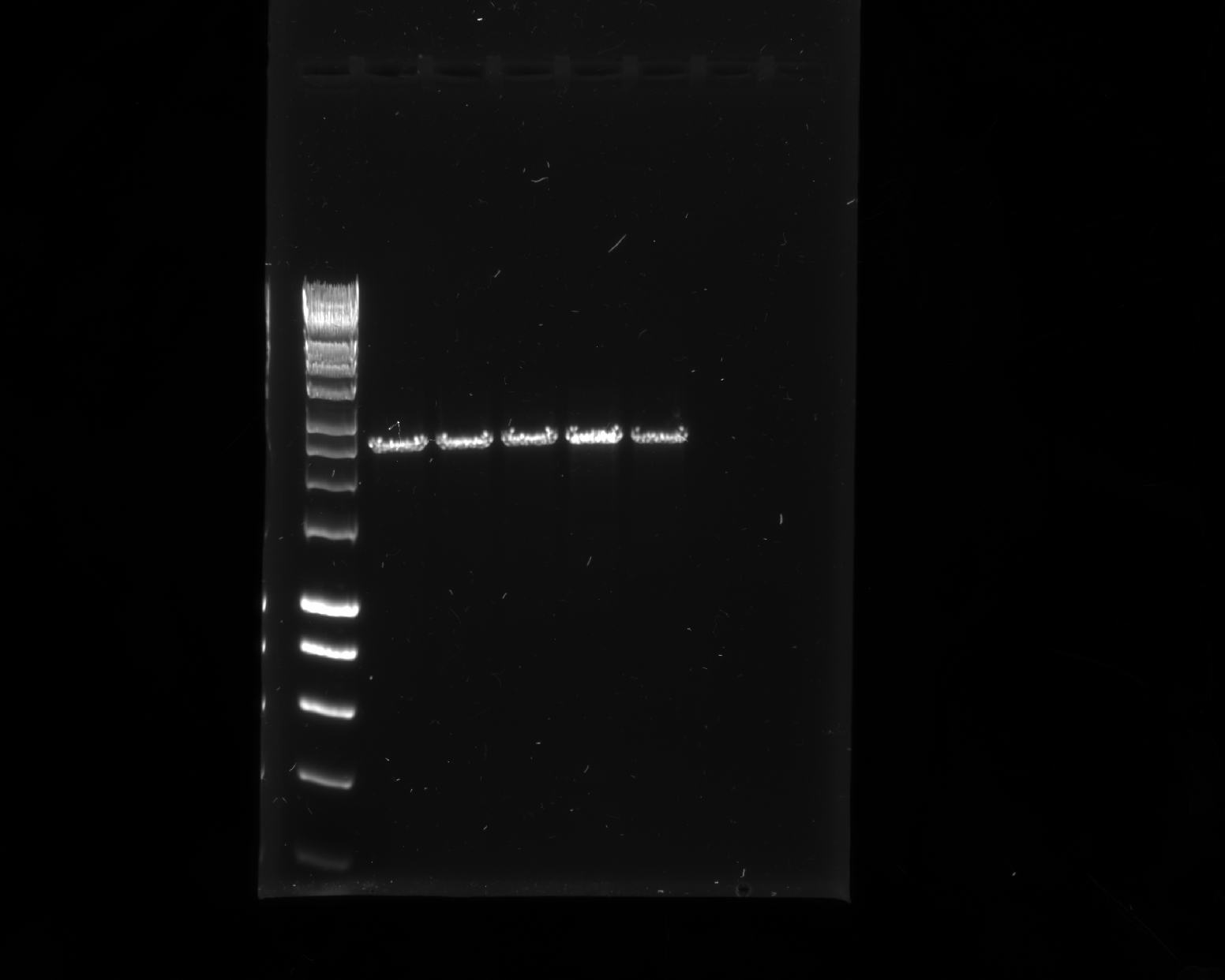

Supplement: Figure 6—figure supplement 1—source data 2. [file elife-107860-fig6-figsupp1-data2.zip › Figure 6 - Supplement 1 - Source Data 2 - Raw Images/Figure 6 - Supplement 1 - Source Data 2 - Raw Image.png]
