# Supplementary material for: Physiological febrile heat stress increases cytoadhesion through increased protein trafficking of Plasmodium falciparum surface proteins into the red blood cell
Source: eLife. 2026 May 13;14:RP107860. doi: 10.7554/eLife.107860 (PMC13171106; doi:10.7554/eLife.107860)

Figure 3 – Supplement 4 – Source Data 2

Uncropped immunofluorescence microscopy images showing HSP70x-3xHA detected with anti-HA (488 nm), KAHRP detected with anti-KAHRP (594 nm), parasite DNA stained with DAPI and the corresponding DIC images. Channels are shown separately.

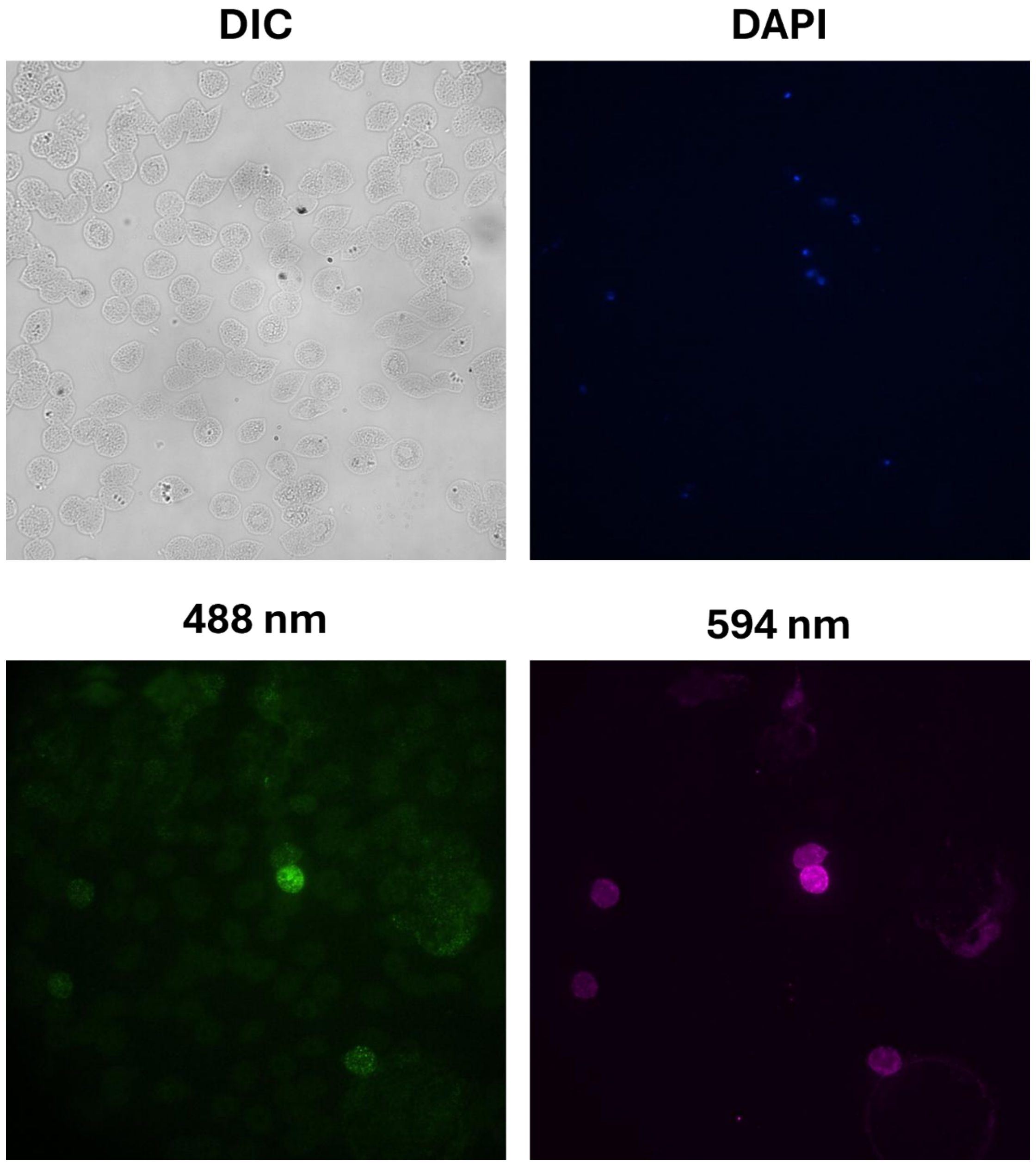

DIC

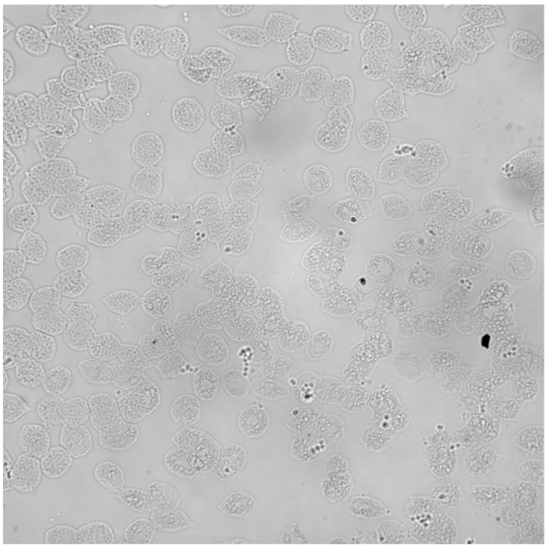

DAPI

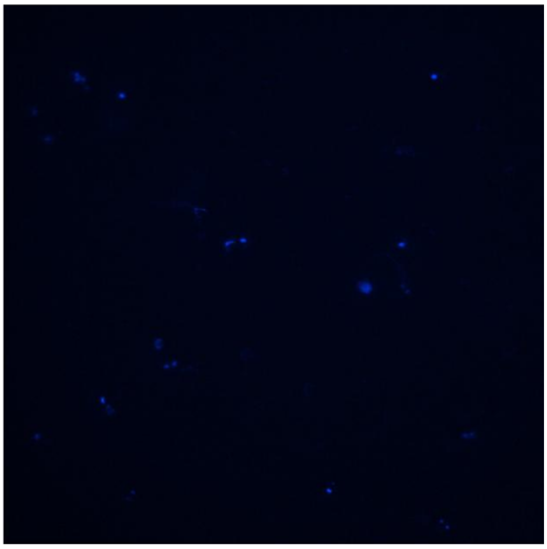

488 nm

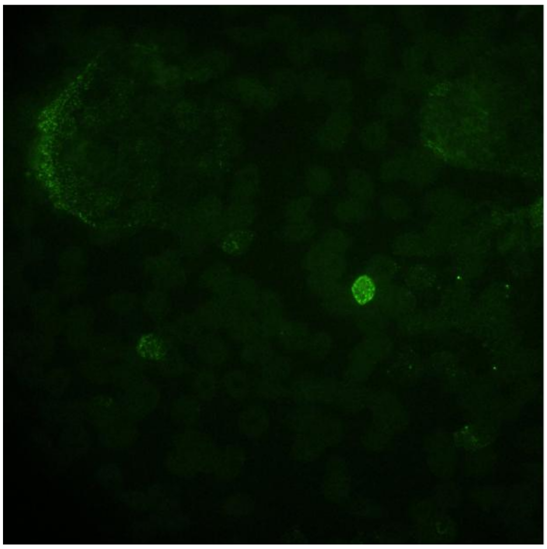

594 nm

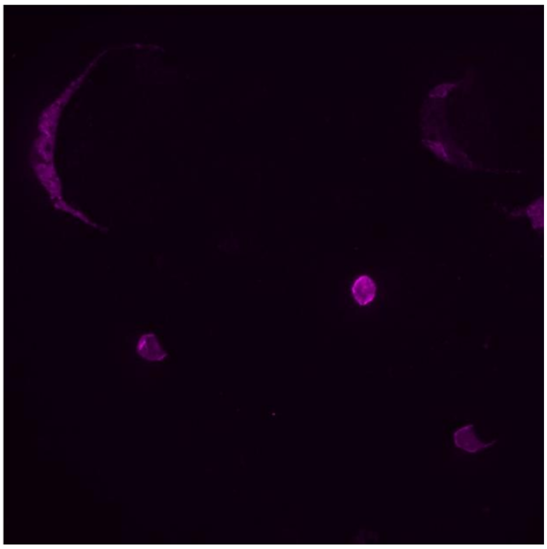

Supplement: Figure 3—figure supplement 4—source data 4. [file elife-107860-fig3-figsupp4-data4.pdf]
