# Supplementary material for: Physiological febrile heat stress increases cytoadhesion through increased protein trafficking of Plasmodium falciparum surface proteins into the red blood cell
Source: eLife. 2026 May 13;14:RP107860. doi: 10.7554/eLife.107860 (PMC13171106; doi:10.7554/eLife.107860)

Figure 3 – Supplement 5 – Source Data 1

Uncropped agarose DNA gel showing PCR products used to assess the integration of PF3D7_0702500-3xHA. The red boxed area indicates the region presented in the manuscript. For completeness, a higher-contrast image of the same gel is also provided below.

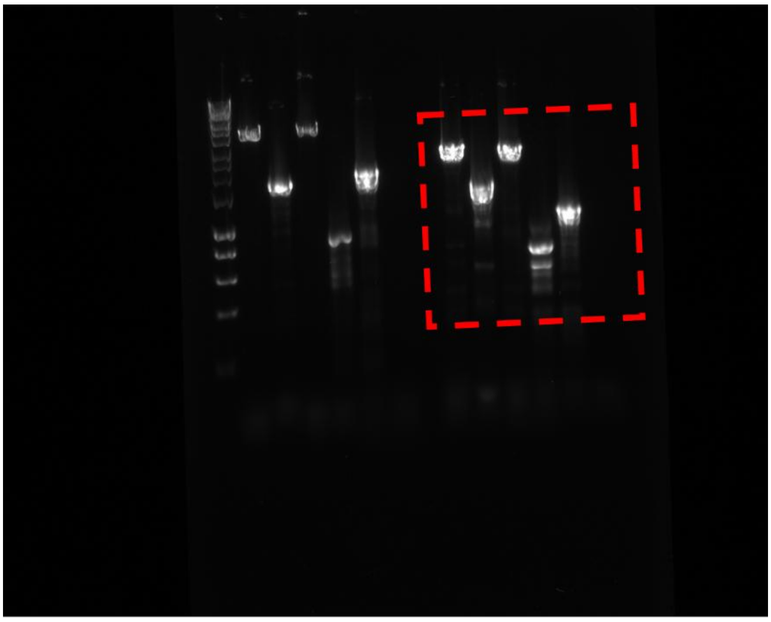

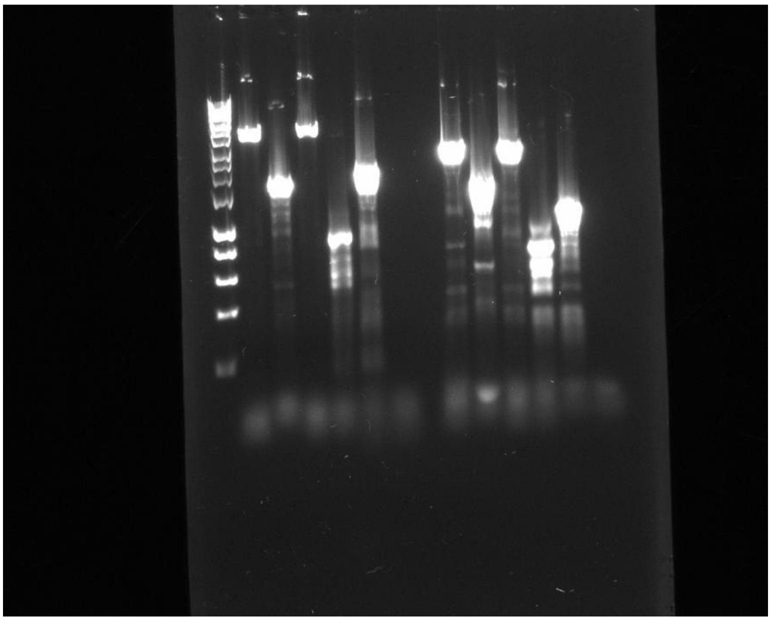

Supplement: Figure 3—figure supplement 5—source data 4. [file elife-107860-fig3-figsupp5-data4.pdf]
