# Supplementary material for: Physiological febrile heat stress increases cytoadhesion through increased protein trafficking of Plasmodium falciparum surface proteins into the red blood cell
Source: eLife. 2026 May 13;14:RP107860. doi: 10.7554/eLife.107860 (PMC13171106; doi:10.7554/eLife.107860)

Figure 3 - Supplement 5 - Source Data 2

Uncropped nitrocellulose membrane immunoblotted with anti-HA showing DMSO and RAP-treated HSP70x-3xHA parasite lysates alongside the wild-type parental strain (*Plasmodium falciparum* NF54 DiCre) lysate. The red boxed area indicates the region presented in the manuscript.

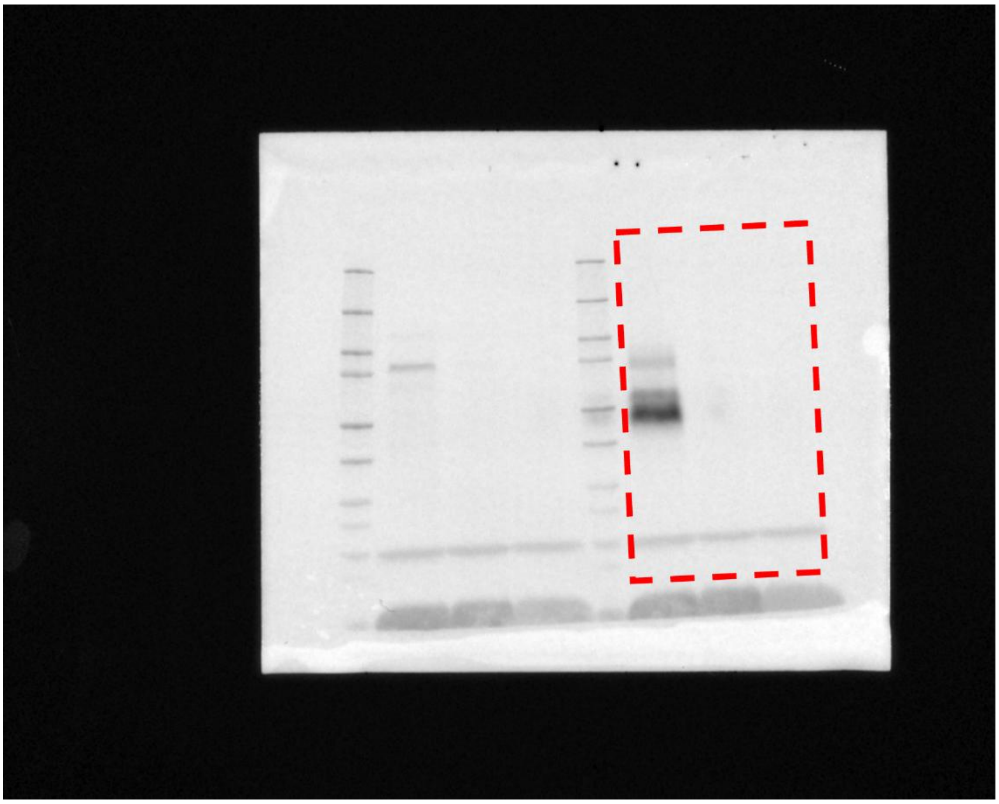

Supplement: Figure 3—figure supplement 5—source data 5. [file elife-107860-fig3-figsupp5-data5.pdf]
