# Supplementary material for: Physiological febrile heat stress increases cytoadhesion through increased protein trafficking of Plasmodium falciparum surface proteins into the red blood cell
Source: eLife. 2026 May 13;14:RP107860. doi: 10.7554/eLife.107860 (PMC13171106; doi:10.7554/eLife.107860)

Figure 4 – Supplement 1 – Source Data 3

Uncropped agarose DNA gel showing PCR products used to assess the correction integration of FIKK10.2-TurboID. The red boxed area indicates the region presented in the manuscript.

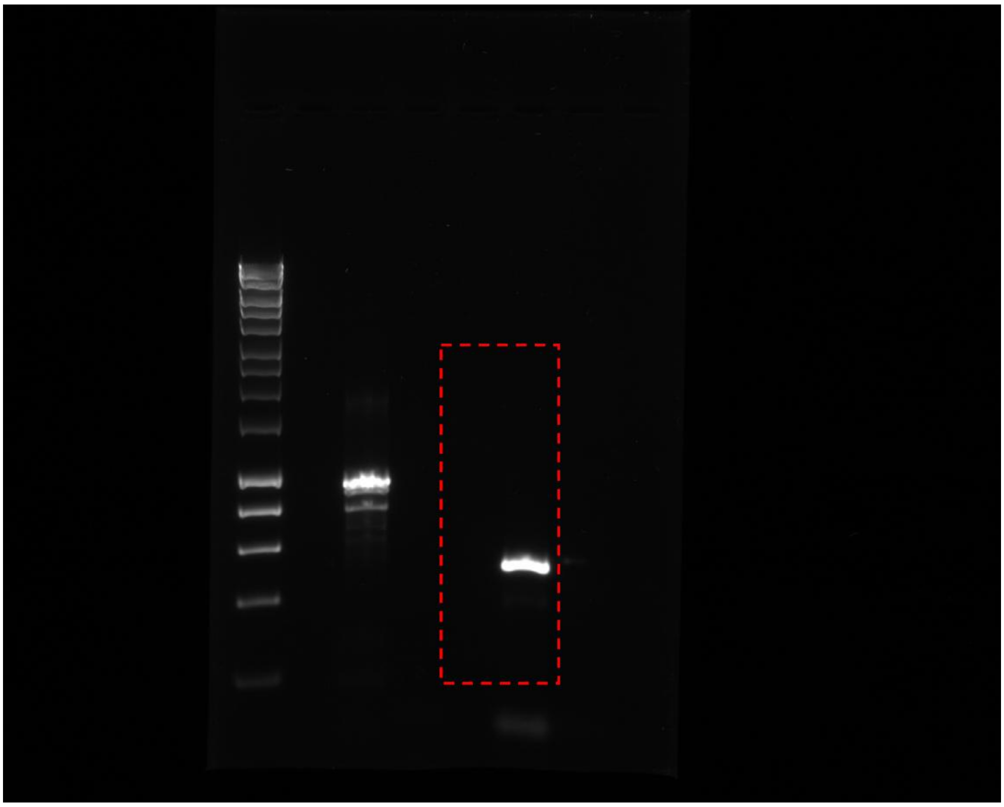

Supplement: Figure 4—figure supplement 1—source data 8. [file elife-107860-fig4-figsupp1-data8.pdf]
