# Supplementary material for: Physiological febrile heat stress increases cytoadhesion through increased protein trafficking of Plasmodium falciparum surface proteins into the red blood cell
Source: eLife. 2026 May 13;14:RP107860. doi: 10.7554/eLife.107860 (PMC13171106; doi:10.7554/eLife.107860)

Figure 4 – Supplement 1 – Source Data 4

Uncropped nitrocellulose membrane immunoblotted with anti-Biotin FIKK10.2-TuboID expressing parasite lysates alongside the wild-type parental strain (*Plasmodium falciparum* NF54 DiCre) lysate. The red boxed area indicates the region presented in the manuscript.

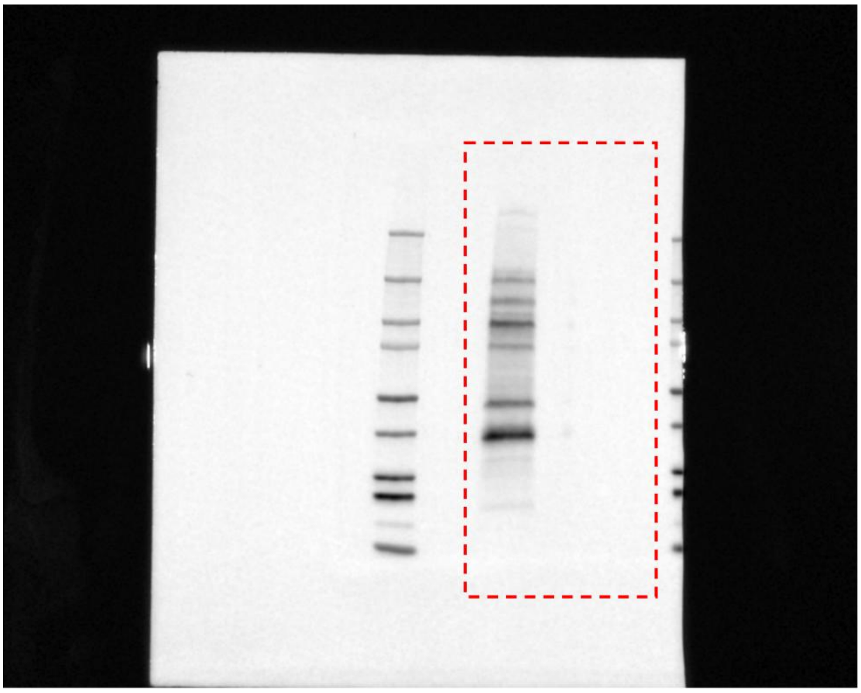

Supplement: Figure 4—figure supplement 1—source data 9. [file elife-107860-fig4-figsupp1-data9.pdf]
