# Supplementary material for: Physiological febrile heat stress increases cytoadhesion through increased protein trafficking of Plasmodium falciparum surface proteins into the red blood cell
Source: eLife. 2026 May 13;14:RP107860. doi: 10.7554/eLife.107860 (PMC13171106; doi:10.7554/eLife.107860)

Figure 4 - Supplement 1 - Source Data 5

Uncropped nitrocellulose membrane showing total protein content detected using the VersaBlot™ Total Protein Normalization Kit CF-680T, including FIKK10.2-TuboID expressing parasite lysates alongside the wild-type parental strain (Plasmodium falciparum NF54 DiCre) lysate. The red boxed area indicates the region presented in the manuscript. For completeness, a higher-contrast image of the same blot is also provided below.

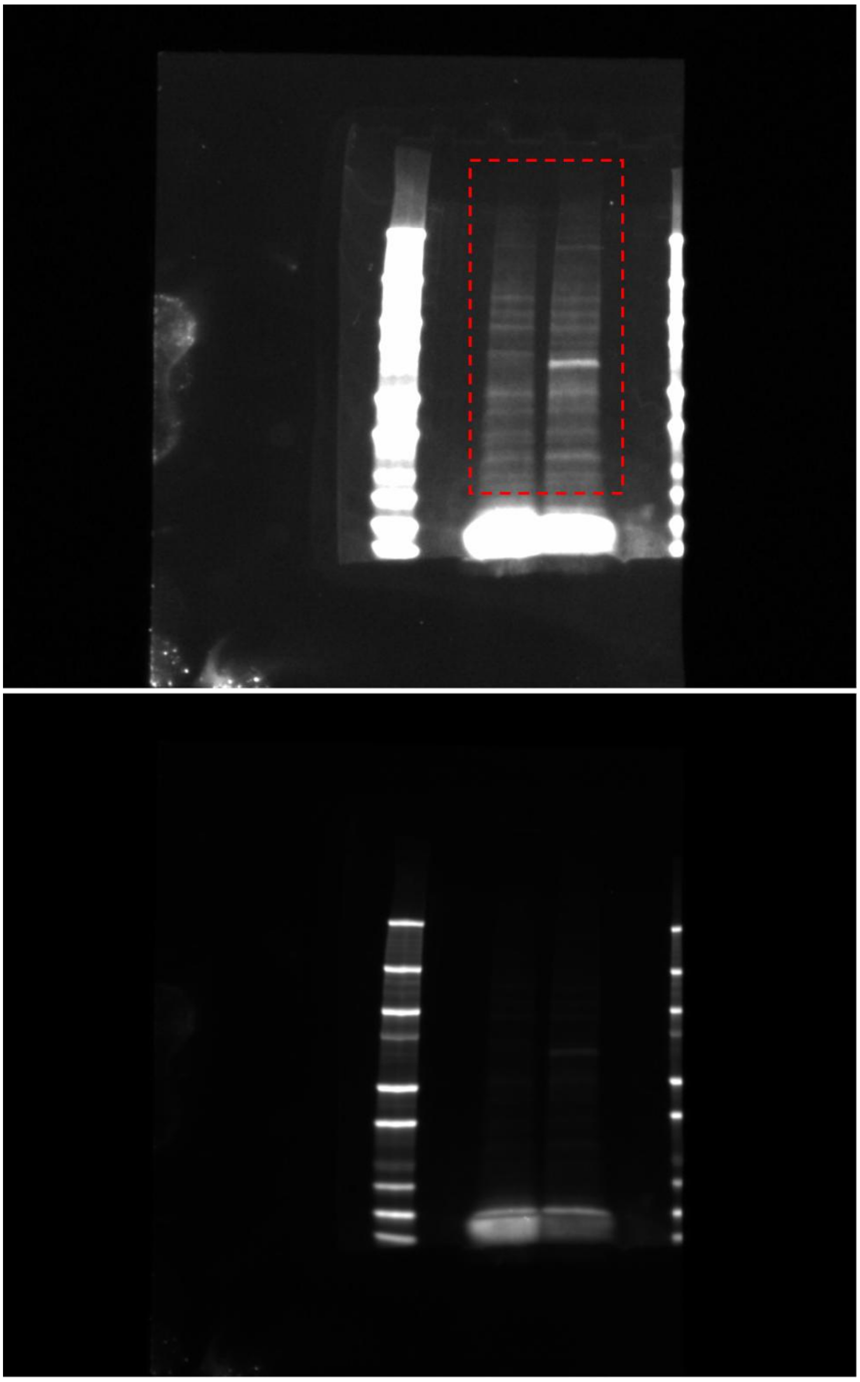

Supplement: Figure 4—figure supplement 1—source data 10. [file elife-107860-fig4-figsupp1-data10.pdf]
