# Supplementary material for: Physiological febrile heat stress increases cytoadhesion through increased protein trafficking of Plasmodium falciparum surface proteins into the red blood cell
Source: eLife. 2026 May 13;14:RP107860. doi: 10.7554/eLife.107860 (PMC13171106; doi:10.7554/eLife.107860)

Figure 5 – Supplement 1 – Source Data 1

Uncropped agarose DNA gel showing PCR products used to assess the 5’ integration of VAR2CSA-TurboID-3xHA. The red boxed area indicates the region presented in the manuscript.

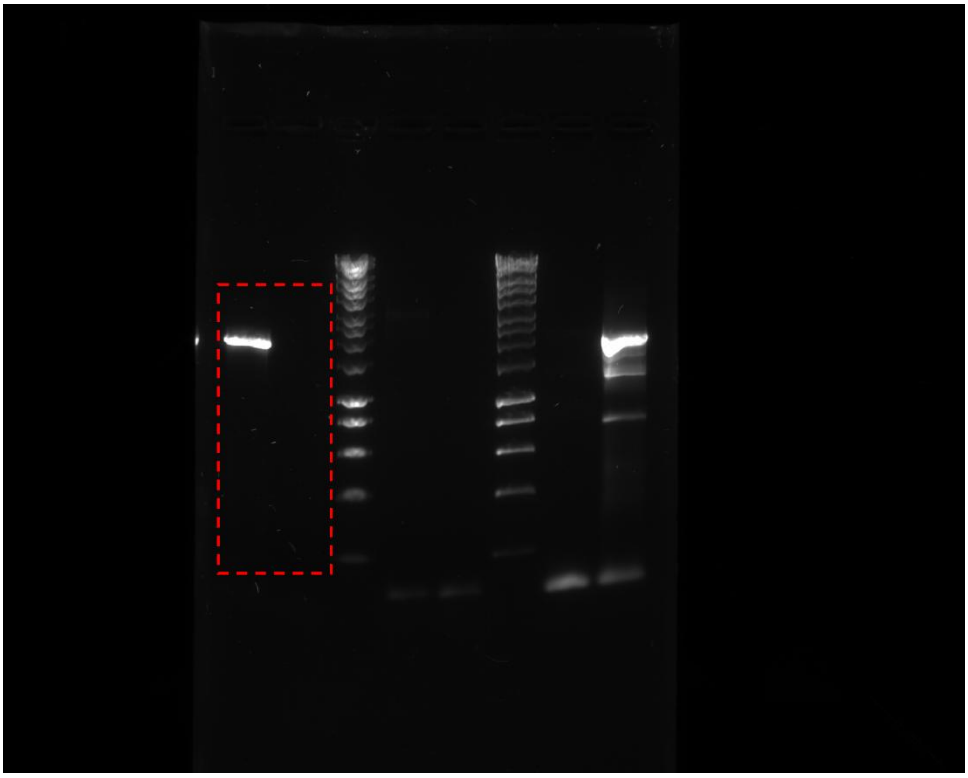

Supplement: Figure 5—figure supplement 1—source data 6. [file elife-107860-fig5-figsupp1-data6.pdf]
