# Supplementary material for: Physiological febrile heat stress increases cytoadhesion through increased protein trafficking of Plasmodium falciparum surface proteins into the red blood cell
Source: eLife. 2026 May 13;14:RP107860. doi: 10.7554/eLife.107860 (PMC13171106; doi:10.7554/eLife.107860)

Figure 5 – Supplement 1 – Source Data 5

Uncropped protein gel (pre-transfer to nitrocellulose membrane) showing total protein content detected using the VersaBlot™ Total Protein Normalization Kit CF-680T, including VAR2CSA-TurboID-3xHA expressing parasite lysates alongside the wild-type parental strain (*Plasmodium falciparum* NF54 DiCre) lysate. The red boxed area indicates the region presented in the manuscript.

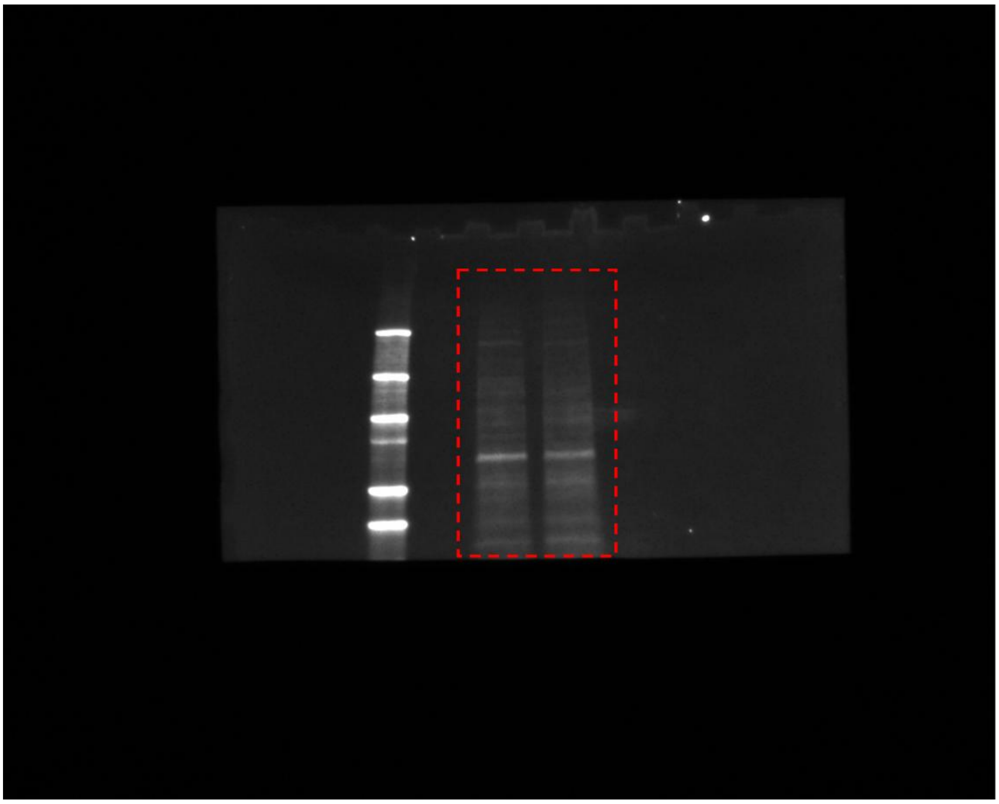

Supplement: Figure 5—figure supplement 1—source data 10. [file elife-107860-fig5-figsupp1-data10.pdf]
