# Supplementary material for: Physiological febrile heat stress increases cytoadhesion through increased protein trafficking of Plasmodium falciparum surface proteins into the red blood cell
Source: eLife. 2026 May 13;14:RP107860. doi: 10.7554/eLife.107860 (PMC13171106; doi:10.7554/eLife.107860)

Figure 6 – Supplement 1 – Source Data 2

Uncropped agarose DNA gel showing PCR products used to assess the 3′ integration of four constitutively expressed NanoLuciferase protein fusions: REX3-NanoLuc (REX3-NL), PF3D7_0702500-NanoLuc (PF3D7_0702500-NL), REX3(TMD)-NanoLuc (REX3(TMD)-NL) and NanoLuc alone (NL). The red boxed area indicates the region presented in the manuscript.

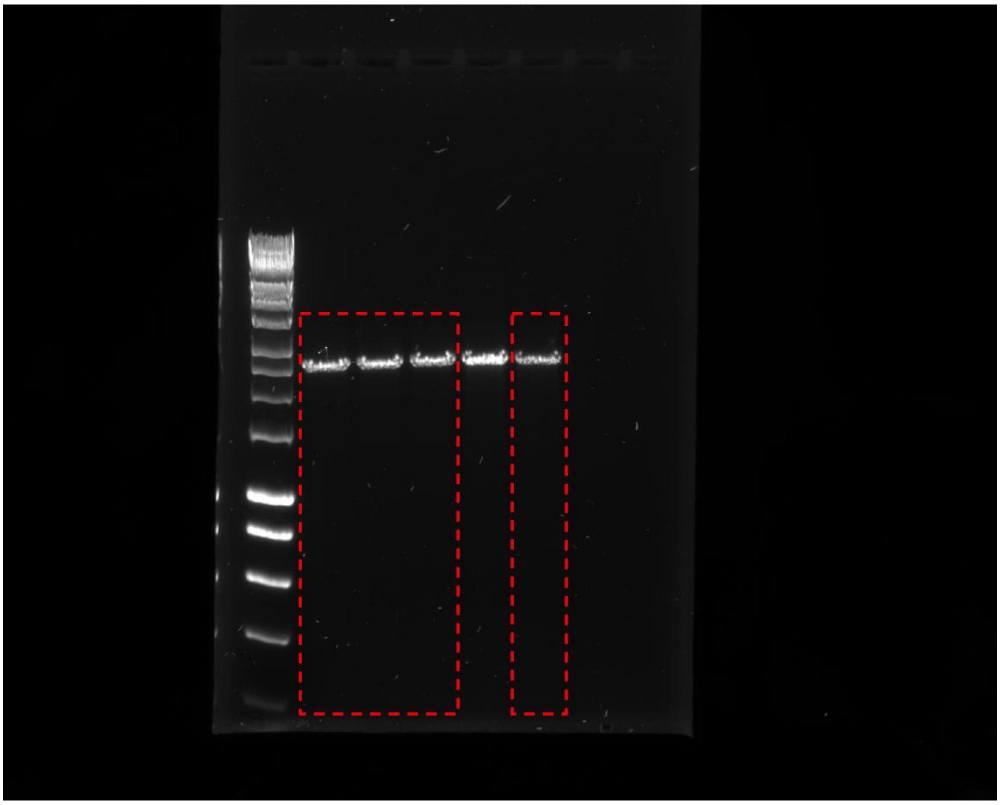

Supplement: Figure 6—figure supplement 1—source data 4. [file elife-107860-fig6-figsupp1-data4.pdf]
